# The Plant Communities of the Class *Isoëto-Nanojuncetea* in Sicily

**DOI:** 10.3390/plants11091214

**Published:** 2022-04-29

**Authors:** Salvatore Brullo, Cristian Brullo, Saverio Sciandrello, Gianmarco Tavilla, Salvatore Cambria, Valeria Tomaselli, Vincenzo Ilardi, Gianpietro Giusso del Galdo, Pietro Minissale

**Affiliations:** 1 Department of Biological, Geological and Environmental Sciences, University of Catania, Via A. Longo 19, 95125 Catania, Italy; salvo.brullo@gmail.com (S.B.); cbrullo@tiscali.it (C.B.); s.sciandrello@unict.it (S.S.); gianmarco.tavilla@phd.unict.it (G.T.); cambria_salvatore@yahoo.it (S.C.); g.giusso@unict.it (G.G.d.G.); 2 Department of Biology, University of Bari Aldo Moro, Via Orabona 4, 70125 Bari, Italy; valeria.tomaselli@uniba.it; 3 Department of Biological, Chemical and Pharmaceutical Sciences and Technologies (STEBICEF), University of Palermo, Viale Delle Scienze, ed. 17, 90128 Palermo, Italy; vincenzo.ilardi@unipa.it

**Keywords:** *Isoëto-Nanojuncetea* class, temporary ponds, Sicily, wetlands, Habitat Directive, phytosociology, plant ecology

## Abstract

A syntaxonomical revision of the *Isoëto-Nanojuncetea* class for the Sicilian territory is provided. This syntaxon gathers the ephemeral herbaceous hygrophilous plant communities linked to periodically submerged soils, widely distributed in the European, circum-Mediterranean and Macaronesian territories. Within this class, two orders are recognized, *lsoëtetalia*, with a prevalently Mediterranean distribution, and *Nanocyperetalia* chiefly occurring in the central-European and Atlantic territories, with scattered and marginal occurrence in the Mediterranean area. The order *Isoëtetalia* in Sicily is represented by four alliances, i.e., *Isoëtion*, *Preslion cervinae*, *Cicendio-Solenopsion laurentiae* and *Agrostion salmanticae*, while within *Nanocyperetalia* three alliances can be recognized, namely *Nanocyperion*, *Verbenion supinae* and *Lythrion tribracteati*. Overall, 32 plant communities are recognized, 11 of which are described for the first time. Each higher rank syntaxa and related associations are examined from a nomenclatural, floristic, ecological and chorological point of view. In particular, the associations were processed using cluster analysis in order to highlight the correlations between them. Regarding the floristic aspects, a checklist of the species occurring in the phytosociological relevés is provided, as well as a new combination concerning *Solenopsis gasparrinii*, a critical species of the Sicilian flora, is proposed.

## 1. Introduction

Wet temporary submerged habitats are generally featured by very specialized vegetation characterized by the dominance of therophytes, often with a short biological cycle, which is sometimes associated with dwarf geophytes and hemicryptophytes. It is ephemeral vegetation, often with quite high plant diversity, where rare or uncommon hygrophytes, mostly exclusive to these environments, find their optimal growing conditions. Such plant communities occur in the European and circum-Mediterranean territories, ranging from the shoreline to the high-mountain stands, especially in relation to the substrate and microclimatic conditions. From the phytosociological viewpoint, this hygrophilous vegetation is usually included in *Isoëto-Nanojuncetea* Br.-Bl. & R. Tx. ex Westhoff, Dijk & Passchier 1946, a rather tricky class whose classification is quite controversial, undergoing several nomenclatural and syntaxonomical changes over time. Besides, the floristic set of the plant communities belonging to this class shows considerable variability in relation to the substrate, bioclimatic conditions, elevation, period of submersion and extension of the surfaces.

These wetlands, due to their floristic peculiarities, have always aroused the interest of botanists and especially phytosociologists, who have tried to highlight the geobotanical relevance and the great naturalistic value of these habitats. Their significant ecological role is recognized by the Habitat Directive (43/92 EEC), identifying them as a priority conservation habitat with the cod. 3170* (Mediterranean temporary ponds) [1]. The first investigations regarding the *Isoëto-Nanojuncetea* communities were carried out by Allorge [2], Koch [3], Braun-Blanquet [4,5], Klika [6], Moor [7,8], Vlieger [9], Tuxen [10], Diemont et al. [11], Zohary & Orshansky [12], Molinier & Tallon [13], Ubriszy [14], who contributed understanding this rare and interesting hygrophilous vegetation occurring both in the Mediterranean and European territories. Later, several other authors studied this vegetation in the whole Euro-Mediterranean area, such as Braun-Blanquet et al. [15], Chevassut & Quezel [16,17], Braun-Blanquet [18], Rivas Goday et al. [19]; Rivas Goday [20,21,22], Horvatic [23], Barbero [24], Philippi [25], Wojcik [26], Bolòs et al. [27], Aubert & Loisel [28], Sunding [29], Pietsch [30], Tüxen & Zevaco [31], de Foucault [32,33,34], Traxel [35], Brullo & Furnari [36], Popiela [37,38], Brullo & Minissale [39], Täuber [40], Taran [41], Deil [42,43], Molina [44], Taran & Laktionov [45], Molina et al. [46], Gigante et al. [47], Šumberová & Hrivnák [48], Kovalenko [49], Korotka & Pashkevych [50], Tomaselli et al. [51], Kącki et al. [52], significantly improving the knowledge of this class. As concerns the Italian territory, the plant communities belonging to the *Isoëto-Nanojuncetea* class are quite investigated. In particular, among the several authors who dealt with this type of ephemeral hygrophilous vegetation in various localities of the Italian peninsula are worth to be mentioned: Pignatti [53,54,55], Anzalone & Caputo [56], Veri et al. [57], Filipello & Sartori [58], Pedrotti et al. [59], Pedrotti [60,61,62], Piccoli [63], Biondi et al. [64,65], Foggi & Grigioni [66], Brullo et al. [67]; Biondi & Vagge [68], Foggi et al. [69], Gigante et al. [47,70], Carta [71], Ceschin & Salerno [72], Ernandes et al. [73], Tomaselli et al. [51]. As concerns the Sardina island, this class was examined by De Marco & Mossa [74], Biondi & Bagella [75], Paradis & Finidori [76], Bagella et al. [77], etc., while from Sicily it was studied by Brullo & Di Martino [78], Brullo & Marcenò [79], Brullo et al. [80,81,82], Barbagallo et al. [83], Brullo & Grillo [84], Marcenò & Trapani [85], Raimondo [86], Minissale & Spampinato [87], Bartolo et al. [88], Brullo & Minissale [39], Brullo & Sciandrello [89], Sciandrello [90,91], Minissale et al. [92], Minissale & Sciandrello [93]. Based on these data, the *Isoëto-Nanojuncetea* class is represented throughout the Italian territory by two orders, such as *Isoëtalia* Br.-Bl. 1936 and *Nanocyperetalia* Klika 1935. The alliances belonging to the first one are *Isoëtion* Br.-Bl. 1936, *Preslion cervinae* Br.-Bl. ex Moor 1937, *Cicendio-Solenopsion laurentiae* Brullo & Minissale 1998 and *Agrostion salmanticae* Rivas Goday 1958, while those ones of the second order are *Nanocyperion* Koch 1926, *Verbenion supinae* Slavnic 1951, *Lythrion tribracteati* Rivas Goday & Rivas-Mart. ex Rivas Goday 1970, *Elatino-Eleocharition ovatae* Pietsch in Pietsch & Müller-Stoll 1968 and *Cicendion* (Rivas Goday in Rivas Goday & Borja 1961) Br.-Bl. 1967 (=*Radiolion linoidis* Pietsch 1973).

In order to increase the knowledge about the *Isoëto-Nanojuncetea* class in Sicily, a contribution concerning the whole territory, including the small surrounding islets, is provided. Based on literature and several unpublished phytosociological relevés, the results of a deep investigation are given, in which all the syntaxa detected are examined under the floristic, ecological, physiognomic-structural, chorological, and nomenclatural viewpoint.

### 1.1. Study Area

**Figure 1:**
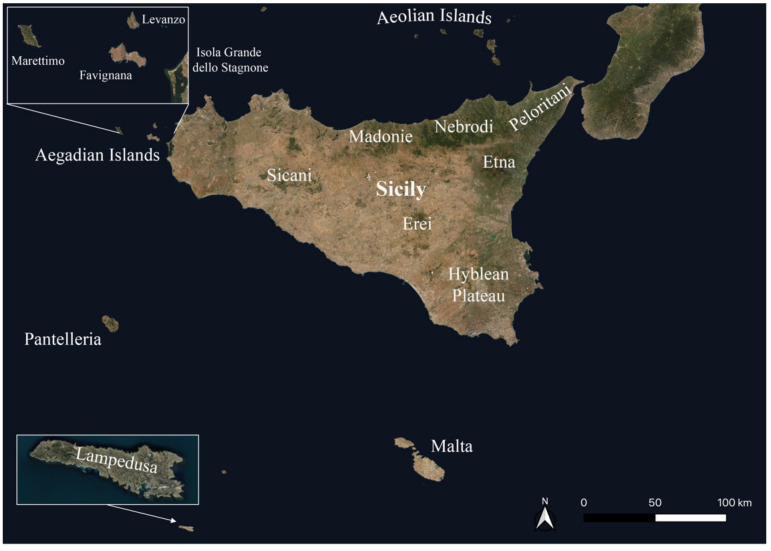
Sicily map from ESRI basemap imagery (modified).

The research covers the whole of Sicily, including some neighboring Sicilian islands (Favignana, Isola Grande dello Stagnone, Pantelleria, Lampedusa). This island is situated in the central Mediterranean and represents the southernmost part of the Italian territory (Figure 1). It is the largest island among those present in the Mediterranean with an area of 25,832.4 km^2^ (including the smaller islands) and with a coastline 1637 km long, of which 1152 km regard the main island. It is surrounded to the north by the Tyrrhenian Sea, to the south-west by the Sicilian Channel, to the east by the Ionian Sea, and is separated to the north-east by the Italian peninsula by the Strait of Messina.

The territory is mainly hilly-mountainous with about 15% of the flat surface represented for the most part by the plain of Catania and the plain of Gela. The most extensive mountain ranges are located along the northern part of the island and are represented by the Peloritani (highest peak, Montagna Grande 1374 m), Nebrodi chain (highest peak, Monte Soro 1847 m) and Madonie massif (highest peak, Pizzo Carbonara 1979 m). Another particularly significant mountainous area is Mt. Etna on the north-eastern side, with its 3357 m elevation it is the highest active volcano in Europe. Other mountainous reliefs are Mt. Lauro (987 m) in the Hyblean plateau, the Sicani mountains with several peaks (i.e., Rocca Busambra, 1613 m; Mt. Cammarata, 1578 m; Mt. delle Rose, 1436 m; La Pizzuta 1333 m; Mt. Kumeta, 1.233 m; etc.), and the Erean mountains (highest peak, Mt. Altesina 1192 m).

Furthermore, of great importance for the investigations concerning this study, are the wetlands, that in Sicily are represented by various natural lakes, as well as by artificial basins and perennial watercourses. In particular, the most important lakes are the Biviere of Cesarò, the ponds of Nebrodi mountains, Biviere of Gela, Lentini lake, Preola and Gorghi Tondi, Gurrida, Pergusa, Pantano Gurna, etc. In addition, there are many artificial basins created in the last century, such as Ogliastro, Pozzillo, Arancio, Scanzano, Piana degli Albanesi, Disueri, Ancipa, etc. Among the several rivers that flow on the island are the Salso, Simeto, Belice, Platani, Verdura, Irminio, Dirillo, Alcantara, Anapo, San Leonardo, Cassibile, Fiumefreddo, Ippari, Ciane, etc.

The Sicilian territory shows a very complex geological history with rocky outcrops dated between the Paleozoic and Quaternary ages [94]. In particular, the oldest substrata belonging to Calabride chain units occurring in the Peloritani area (North-East Sicily) are represented by Paleozoic metamorphic rocks. The Nebrodi chain is characterized mainly by siliceous rocks from the late Mesozoic to Oligocene belonging to Numidian Flysch. In the Madonie massif, the substrata are mainly represented by carbonatic, dolomitic and quarzarenitic rocks from the Mesozoic age mainly belonging to Panormide units. The north-western sector of Sicily and Aegadian Islands are prevalently constituted by Mesozoic carbonatic rocks, as well as the Sicani mountains. The Hyblean territory is characterized by Miocene limestones deeply carved by river valleys (caves), which are covered in the upstate by a layer of Plio-Pleistocenic lava. Most of southern and central Sicily is constituted by Plio-Pleistocenic rocks belonging to the Messinian evaporitic series (clays, sandstones, limestones, marls, gypsums, etc.). Finally, Mt. Etna is an active volcanic cone covered by basalt rocks from the Quaternary age, as well as Pantelleria island.

According to Rivas-Martínez [95] classification, two bioclimates can be identified in the Sicilian territory as reported by Bazan et al. [96], such as Mediterranean pluviseasonal oceanic occurring in almost the whole island, including the Aegadian Islands and Aeolian archipelago and Mediterranean xeric oceanic circumscribed exclusively to the coastal belt near Gela, Pantelleria and Pelagie islands. Besides, based on the investigations carried out by Brullo et al. [97] and Bazan et al. [96], 23–25 bioclimatic units can be recognized on the island. Each bioclimatic unit is closely linked to well defined climatophilous associations, that highlight the close correlations between the vegetation and the climatic conditions of a territory. In particular, it is possible to identify several thermotypes and ombrotypes based on the climatic trend regarding the monthly and annual monitoring of the available thermo-pluviometric stations. Among them, along the coastal belt, the lower Thermomediterranean lower dry type is predominant, while in the hilly areas the upper Thermomediterranean lower dry type is very spread out. Another type, quite frequent in Sicily, is the lower Mediterranean upper-lower dry type linked to sub-mountainous places, which in mountainous places is replaced mainly by upper Mesomediterranean at lower altitudes and lower Supramediterranean at upper ones, with upper dry to lower subhumid ombrotypes.

**Figure 2:**
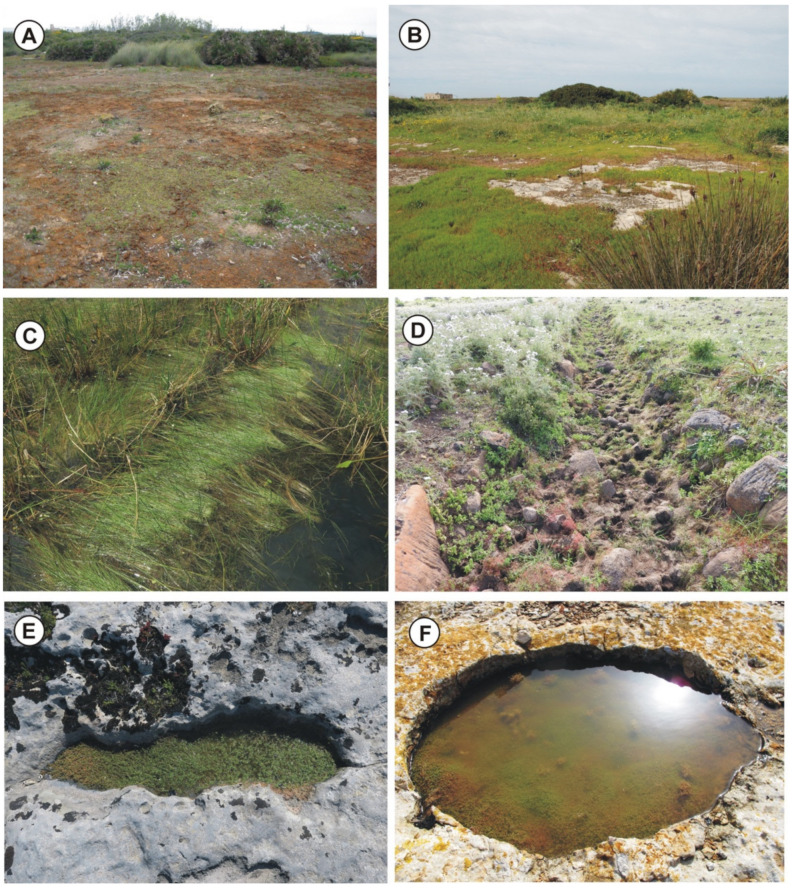
Sicilian habitats colonized by plant communities belonging to *Isoëto-Nanojuncetea*: (**A**) Waterlogged soils from Isola Lunga dello Stagnone with *Solenopsietum mothianae*; (**B**) Cupular pools from Isola Lunga dello Stagnone with vegetation of *Isoëtion*; (**C**) Temporary streams from Anguillara (Catalafimi) with *Isoetes longissima* population; (**D**) Drainage ditches from Cozzo Ogliastri (Sortino) with *Junco pygmaei-Pilularietum minutae*; (**E**) Cupular pools from Hyblean Plateau (Modica) with *Lythro hyssopifoliae-Elatinetum macropodae*; (**F**) Cupular pools from Lampedusa Island with *Crassulo vaillantii-Elatinetum gussonei*. (Photos of the Authors).

**Figure 3:**
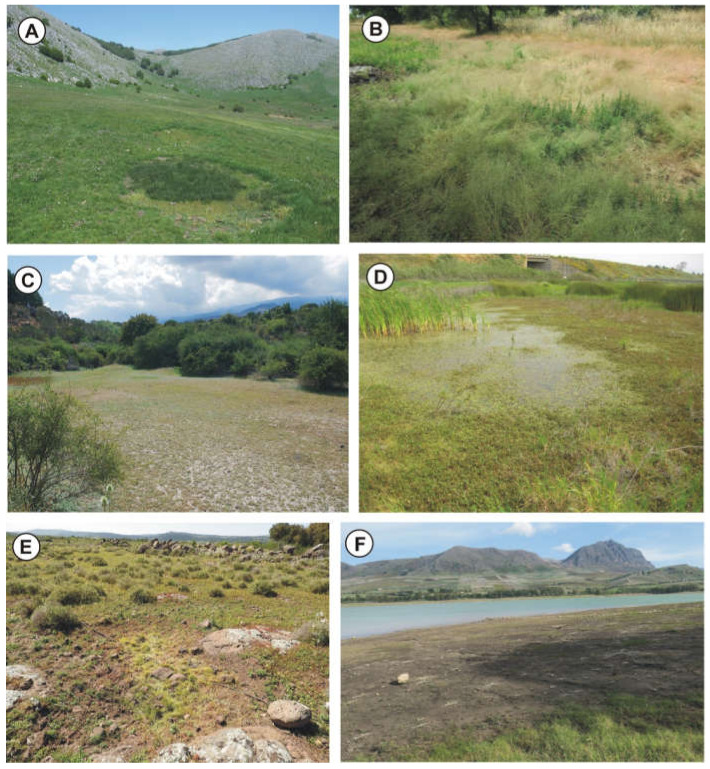
Sicilian habitats colonized by plant communities belonging to *Isoëto-Nanojuncetea*: (**A**) Doline from Piano Battaglia (Madonie) with *Myosuro minimi-Ranunculetum lateriflori*; (**B**) Large wetlands from Ficuzza with *Trifolio micheliani-Agrostidetum pourretii*; (**C**) Temporary streams from Gurrida Lake (Randazzo) with *Coronopo squamati-Sisymbrelletum dentatae*; (**D**) Temporary streams from Piana del Signore (Gela) with *Damasonio bourgaei-Crypsietum aculeatae*; (**E**) Cupular pools from Cozzo Ogliastri (Sortino) with *Archidio phascoidis-Isoetetum velatae*; (**F**) Artificial basin from Piana degli Albanesi (Palermo) with *Glino lotoidis-Verbenetum supinae*. (Photos of the Authors).

**Figure 4:**
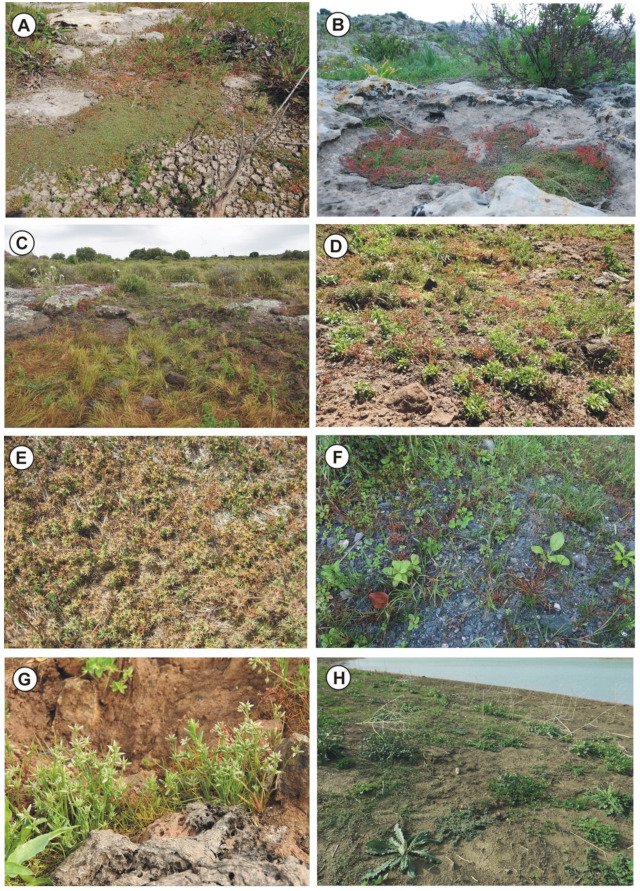
Sicilian habitats colonized by plant communities belonging to *Isoëto-Nanojuncetea*: (**A**) Calcarenitic rocky pools from Isola Lunga dello Stagnone with *Buillardio vaillantii-Elatinetum campylospermae*; (**B**) Calcareous rocky pools from Syracuse with *Lythro hyssopifoliae-Elatinetum macropodae*; (**C**,**D**) Basaltic rocky pools from Cozzo Ogliastri (Sortino) with *Archidio phascoidis-Isoetetum velatae*; (**E**) Temporary streams from Piana del Signore (Gela) with *Damasonio bourgaei-Crypsietum aculeatae*; (**F**) Siliceous temporary streams from Fiumedinisi with *Plantago intermediae-Cyperetum fusci*; (**G**) Drainage ditches from Cozzo Ogliastri (Sortino) with *Junco pygmaei-Pilularietum minutae*; (**H**) Shore of the artificial basin from Poma Lake (Partinico) with *Heliotropio supini-Heleochloetum schoenoidis*. (Photos of the Authors).

In the context of the Sicilian territory, the wetlands colonized by the plant communities object of this study occur from the coastal belt to the high mountains of the northern ranges, up to 1500–1600 m of elevation. The habitats that usually host this vegetation are represented by surfaces periodically flooded during the winter-spring period, gradually drying up in the summer, as well as the shores of lakes, swamps, reservoirs, and waterways. On the basis of the geological origin, edaphic characteristics, the length of submersion period and physicochemical properties of the waters, these habitats have been classified by Ernandes & Marchiori [98] and Ernandes et al. [73] into four main types, such as:

(a) Cupular pools—Better known as rocky pools, which are usually small catchment depressions on flat outcrops arising from limestone dissolution (Figure 2B,E,F, Figure 3E and Figure 4A–D). The bottom of these ponds is covered by a thin layer of soil, submerged by shallow water, characterized by associations regarding the *Isoëtion* or *Preslion cervinae*.

(b) Dolines—They are broad depressed surfaces periodically flooded by rainwater with deep and poorly permeable soils, created by karst phenomena or by subsidence (Figure 3A). Usually, in these habitats, there are communities of the *Preslion crevinae*, *Agrostion salmanticae* and *Verbenion supinae*.

(c) Waterlogged soils—They correspond to more or less large hollows with impermeable surfaces often localized in the wood clearing and covered by a thick layer of clay-silt soil often abundant in the sandy component (Figure 2A and Figure 4G). These stands are submerged by shallow rainwater for short periods, and the plant communities belong mainly to *Cicendio-Solenopsion laurentiae*.

(d) Temporary streams—They are tiny and shallow watercourses, already dried up in late spring but with soils that remain moist for a long time (Figure 2C, Figure 3B–F and Figure 4E,F,H). In this category, the shores of lakes and artificial basins that dry up during the summer can be included. These habitats are colonized by sub-nitrophilous vegetation of *Verbenion supinae* or *Lythrion tribracteati*.

### 1.2. Floristic Considerations

**Figure 5:**
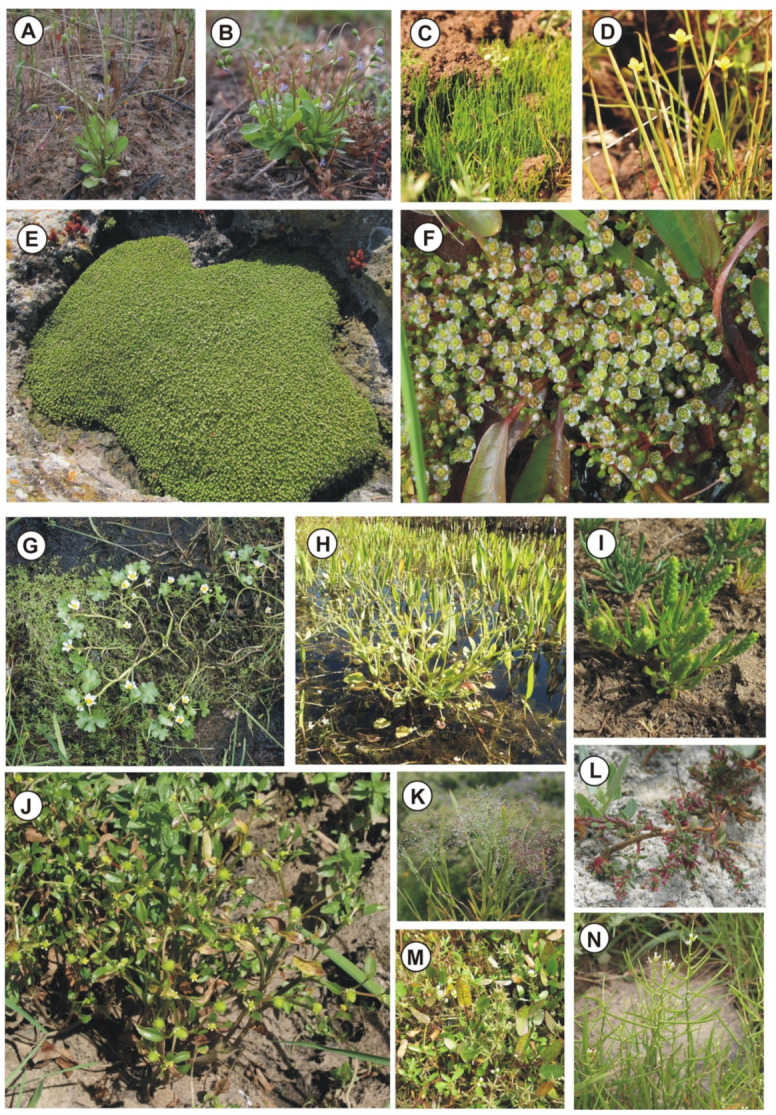
Hygrophilous species occurring in the plant communities of *Isoëto-Nanojuncetea*: (**A**) *Solenopsis gasparrinii* from Anguillara (Catalafimi); (**B**) *Solenopsis laurentia* subsp. *hyblaea* from Cozzo Ogliastri (Sortino); (**C**) *Pilularia minuta* from Cozzo Ogliastri (Sortino); (**D**) *Cicendia filiformis* from Isola Lunga dello Stagnone; (**E**) *Elatine campylosperma* from Castelvetrano; (**F**) *Elatine macropoda* from Syracuse; (**G**) *Ranunculus saniculifolius* and *Callitriche brutia* from Hyblean Plateau; (**H**) *Ranunculus ophioglossifolius* from Cozzo Ogliastri (Sortino); (**I**) *Myosurus minimus* from Madonie; (**J**) *Ranunculus lateriflorus* from Cozzo Ogliastri (Sortino); (**K**) *Anthinoria insularis* from Monte Lauro (Hyblean Plateau); (**L**) *Lythrum tribracteatum* from Piana del Signore (Gela); (**M**) *Damasonium bourgaei* from Piana del Signore (Gela); (**N**) *Sisymbrella dentata* from Gurrida lake (Randazzo). (Photos of the Authors).

The Sicilian wetlands colonized by the plant communities belonging to the *Isoëto-Nanojuncetea* host a well specialized flora, which is very rich in species usually having a remarkable taxonomical and phytogeographical role (Appendix A, Table A1). Such flora is represented by small sized hygrophytes with a prevalently therophytic and cryptophytic life form. In particular, some rare Sicilian endemics are worth to be mentioned, such as *Elatine gussonei*, *Myosotis tineoi*, *Ranunculus angulatus*, *Sisymbrella dentata* (Figure 5N), *Solenopsis laurentia* subsp. *hyblaea* (see Brullo et al. [99]) (Figure 5B), *Solenopsis mothiana*, and *Spergularia madoniaca*. Among this group of endemic species, must be added *Solenopsis gasparrinii* (Tineo) Brullo *comb. et stat. nov.* (Bas.: *Lobelia gasparrinii* Tineo, Cat. Pl. Hort. Panorm. 279, 1827), must be added (Figure 5A). Besides, other rare Mediterranean species are also represented, such as *Agrostis pourretii*, *Barbarea bracteosa*, *Gnaphalium uliginosum* var. *prostratum*, *Isoëtes sicula*, *I. todaroana*, *Lotus conimbricensis*, *Molineriella minuta*, *Myosotis sicula*, *Pilularia minuta* (Figure 5C), *Ranunculus pratensis*, *Teucrium campanulatum*, *Trifolium michelianum.* Several species with a wider Mediterranean, Mediterranean-Atlantic and Euro-Mediterranean distribution are frequent in these habitats, such as *Anagallis parviflora*, *Antinoria insularis* (Figure 5K), *Bulliarda vaillantii*, *Callitriche brutia* (Figure 5G), *Centaurium maritimum*, *Cicendia filiformis* (Figure 5D), *Corrigiola litoralis*, *Damasonium bourgaei* (Figure 5M), *D. polyspermum*, *Elatine campylosperma* (Figure 5E), *E. macropoda* (Figure 5F), *Eryngium pusillum*, *Isoëtes durieui*, *I. longissima*, *Juncus capitatus*, *J. pygmaeus*, *Kickxia cirrhosa*, *Lotus angustissimus*, *L. parviflorus*, *Lythrum tribracteatum* (Figure 5L), *Middendorfia borysthenica*, *Polypogon subspathaceus*, *Pulicaria sicula*, *P. vulgaris*, *Ranunculus ophioglossifolius* (Figure 5H), *R. parviflorus*, *R. saniculifolius* (Figure 5G), *R. trilobus*. Finally, many species with a wide range are rather common, chiefly represented by paleotemperate, circumboreal, paleotropical, subcosmopolitan and cosmopolitan taxa, e.g., *Anagallis minima*, *Briza minor*, *Coronopus squamatus*, *Sporobolus aculeatus*, *S. alopecuroides*, *S. schoenoides*, *Cyperus flavescens*, *C. fuscus*, *C. michelianus*, *Gaudinia fragilis*, *Glinus lotoides*, *Heliotropium supinum*, *Isolepis cernua*, *Juncus bufonius*, *Laphangium luteo-album*, *Lythrum hyssopifolia*, *Mentha pulegium*, *Myosurus minimus* (Figure 5I), *Ophioglossum lusitanicum*, *Plantago intermedia*, *Poa infirma*, *Radiola linoides*, *Ranunculus lateriflorus* (Figure 5J), *Trifolium micranthum*, *Verbena supina.*

### 1.3. History of the Syntaxonomic Treatment of the Isoëto-Nanojuncetea Class

Among the phytosociologists who proposed a first syntaxonomic framework on the ephemeral vegetation of these wetlands, Koch must be mentioned [3], who included the Central-European plant communities in the *Nanocyperion flavescentis* alliance, referring it to the order *Nanocypero-Polygonetalia*, an arrangement followed also by Libbert [100]. The first results of investigations on the Mediterranean communities were provided by Braun-Blanquet [4], who published as *nomina nuda* a list of syntaxa concerning the Languedoc region (S France), including the vegetation of the temporary ponds subject to short periods of submersion within the *Isoëtetalia* order represented by the only one alliance, i.e., *Isoëtion*. Conversely, the communities linked to more prolonged submersions were attributed to *Preslion cervinae*, an alliance of the *Phragmitetalia* order. Later, the same author [5] published a detailed study on the vegetation belonging to *Isoëtion* from some Mediterranean localities with the description of several new associations, while a more comprehensive treatment was provided by Moor [7,8], who recognized a single order *Isoëtetalia*, within which the Central European association of the *Nanocyperion flavescentis* was placed, along with the Mediterranean ones of the *Isoëtion* and *Preslion cervinae.* Besides, other data about the *Nanocyperion* are given by Braun-Blanquet & Moor [101]. The arrangement of this vegetation in a new autonomous class (*Isoëto-Nanojuncetea*) was hypothesized by Braun-Blanquet & Tüxen [102], but this syntaxon, proposed as *nomen nudum*, including only the *Isoëtetalia* order, was later validated by Westhoff et al. [103]. Other syntaxonomical contributions were published by Slavinic [104], who identified a new alliance (*Verbenion supinae*), which he included together with *Nanocyperion* into *Bidentetalia*, as well as by Braun-Blanquet et al. [15], who maintained the less nitrophilous communities, such as those of *Isoëtion*, *Preslion cervinae* and *Nanocyperion* within *Isoëto-Nanojuncetea*, while the more nitrophilous ones were attributed to a new provisional alliance represented by *Heliochloion*, referred to the *Paspalo-Heleochloetalia*, order of *Chenopodietea*. Besides, Rivas Goday et al. [19] agreed with the arrangement proposed by Braun-Blanquet et al. [15], identifying, in addition, the new alliance *Agrostion salmanticae*, namely also *Pre-Isoëtion*, including it in the *Isoëtetalia*, which shows an intermediate position between *Tuberarion guttatae* and *Isoëtion*. Later, Rivas Goday (in Rivas Goday & Boria [105]) and also Rivas Goday [21] within *Nanocyperion flavescentis* distinguished the new sub-alliance *Cicendenion*, typified by the Atlantic association *Cicendietum filiformis* Allorge 1922, syntaxon which afterward was elevated to the rank of an alliance by Braun-Blanquet [18].

The relevant floristic and ecological autonomy of the *Nanocyperion flavescentis* alliance, compared to the other alliances of the *Isoëtetalia*, was first highlighted by Klika [6], who considered it more appropriate to include the associations of the *Nanocyperion flavescentis* in a separate order, which he proposed as *Nanocyperetalia*. Regardless, Lohmeyer et al. [106] included the *Nanocyperion flavescentis* in a new order using an unpublished name proposed by Pietsch and Müller-Stoll [107], such as *Cyperetalia fusci*, considered an illegitimate name. This syntaxomic scheme was later updated by Rivas Goday [22], who within *Isoëto-Nanojuncetea* distinguishes two orders *Isoëtetalia* and *Cyperetalia fusci*, including in the first, *Isoëtion*, *Cicendion* and *Preslion cervinae*, while attributing to the second *Nanocyperion flavescentis* and *Heleochloion* in addition to the new alliance *Lythrion tribracteati*.

A detailed review concerning the European and Mediterranean territories was carried out by Pietsch [30], who within *Isoëto-Nanojuncetea* identified the orders *Isoëtetalia* with two alliances (*Isoëtion* and *Preslion cervinae*), and *Cyperetalia fusci*, which is represented by numerous alliances and sub-alliances; they are *Elatino-Eleocharition ovatae*, with two sub-alliances (*Eu-Eleocharitenion ovatae* and *Elatino-Lindernenion procumbentis*), *Radiolion linoidis* with two sub-alliances (*Cicendenion filiformis* and *Centunculenion minima*), *Eu-Nanocyperion flavescentis* distinguished in two sub-alliances (*Carici pulchellae-Cyperenion* and *Fimbristyli-Cyperenion*), and finally *Heleochloo-Cyperion*. The arrangement proposed by Pietsch [30] was revised and updated by Brullo & Minissale [39], who within the order *Isoëtetalia*, apart from the alliances *Isoëtion* and *Preslion cervinae*, also included *Agrostion pourretii* and the new alliance *Cicendio-Solenopsion laurentiae*. Concerning the *Cyperetalia fusci* order Brullo & Minissale [39] emphasized that from the nomenclature viewpoint the correct name is *Nanocyperion* Klika 1935, and proposed to keep within it the alliance *Radiolion linoidis* Pietsch 1973, whose name, however, must be considered illegitimate and replaced by *Cicendion filiformis* (Rivas Goday in Rivas Goday & Borja 1961) Braun-Blanquet 1967.

Another revision of this class, that is more complex and articulated, was carried out by de Foucault [32], who identified two distinct classes, *Isoëtetea velatae*, including mainly the perennial plant communities distributed prevalently in the EU-Mediterranean territories, and *Juncetea bufonii*, regarding the annual aspects occurring in the Europe and Mediterranean. Within these two classes, he identified various orders, alliances, and sub-alliances which in most cases are not clearly distinct, neither from the floristic viewpoint nor from the ecological one. Subsequently, this arrangement was partly taken up by Gehu [108], who recognized only one class, such as *Isoëto-Nanojuncetea*, including within it all the orders already identified by de Foucault [32], and by adding a new one, the *Cicendetalia filiformis*; while regarding the alliances mentioned by the last author, making only a few changes.

Furthermore, for the Iberian Peninsula, a syntaxonomic scheme is provided by several authors [95,109,110,111], in which only the *Isoëto-Nanojuncetea* class is recognized with two orders *Isoëtetalia* and *Nanocyperetalia*. Of these the first one includes four alliances *Isoëtion*, *Preslion cervinae* (=*Menthion cervinae*), *Agrostion pourretii* and *Cicendion*, while the second one is represented by the *Nanocyperion flavescentis*, *Verbenion supinae* (=*Heleochloion schoenoidis*) and *Lythrion tribacteati*. It should be noted that from a nomenclatural point of view these authors mystified the *Cicendion* (Rivas Goday & Borja 1961) Braun-Blanquet 1967 with the *Cicendio filiformis-Solenopsion laurentiae* Brullo & Minissale 1998. In fact, the *Cicendion* alliance groups together plant communities with a prevalently summer cycle, widespread mainly in the Atlantic coastal places and Central Europe, which are characterized by microphytes with Euro-Mediterranean distribution mixed with mesophilous hygrophytes of *Nanocyperetalia*, while the associations of the *Cicendio filiformis-Solenopsion laurentiae* are distributed exclusively in the Mediterranean areas, where they are localized in markedly thermophilous ponds, with an early spring cycle and mainly linked to humid sandy soils, where the species of *Nanocyperetalia* are completely absent. In particular, *Cicendion* was recorded also in the Italian peninsula from the Aspromonte massif [67], where this alliance is represented by only one association (*Barbareo-Corrigioletum litoralis*) localized in the mountain belt at 1100–1300 m a.s.l.

Another syntaxonomic framework for *Isoëto-Nanojuncetea* was proposed by Biondi & Blasi [112] and Biondi et al. [113] from the Italian territory, in which the syntaxa belonging to this class, known for the peninsula and islands, are listed, and commented. In particular, two orders are recognized, such as *Isoëtetalia durieui* and *Nanocyperetalia flavescentis*; within the first one they included *Isoëtion durieui*, *Menthion cervinae*, *Agrostion pourreti*, *Cicendion filiformis*, *Cicendio filiformis-Solenopsion laurentiae*, while in the second one only the *Nanocyperion flavescentis* and *Verbenion supinae* were attributed. However, it should be noted that in this arrangement there are some incoherences since the alliance *Cicendion filiformis* should not be included in the *Isoëtetalia* because, as proposed by these authors, this is a mere synonym of *Cicendio filiformis-Solenopsion laurentiae*, and it must be referred to the *Nanocyperetalia*. Furthermore, in the latter order, the alliance *Elatino-Eleocharition ovatae* Pietsch in Pietsch & Müller-Stoll 1968 was not listed by the aforesaid authors, while it was previously mentioned from Italy by Pietsch [30] and Brullo & Minissale [39], on the basis of three associations described for the Po Valley by Pignatti [54,55].

More recently, the last arrangement has been proposed by Mucina et al. [114] who, while recognizing the two classic orders, *Isoëtetalia* and *Nanocyperetalia*, within *Isoëto-Nanojuncetea*, reworked the alliances sometimes in an inappropriate way, deviating from the schemes proposed by the previous authors. In particular, they included in *Isoëtetalia* two alliances belonging to the *Nanocyperetalia*, which are *Lythrion tribracteati* and *Cicendion*. Besides, they treated the *Cicendio-Solenopsion laurentiae* as a synonym of the last alliance, but this is an evident mistake since as highlighted before, they are two syntaxa distinct from a floristic, ecological and phenological viewpoint. In fact, *Cicendion* is a priority name over *Radiolion linoidis* that, therefore, must be treated as a nomenclatural synonym. On the other hand, they consider correctly the *Elatino-Eleocharition ovate* Pietsch 1973 as a synonym of the *Eleocharition soloniensis* Philippi 1968 for priority reasons.

Based on these arrangements proposed by the aforesaid authors, it is considered more appropriate that the plant communities of this class occurring in the Italian territory can be framed in the following syntaxonomic scheme:

*Isoëto-Nanojuncetea*
  *Isoëtetalia*
    *Isoëtion*
    *Preslion cervinae*
    *Cicendio-Solenopsion laurentiae*
    *Agrostion salmanticae*
  *Nanocyperetalia*
    *Nanocyperion flavescentis*
    *Elocharition soloniensis*
    *Cicendion*
    *Verbenion supinae*
    *Lythrion tribracteati*

## 2. Results and Discussion

### 2.1. Vegetation Analysis

**Figure 6:**
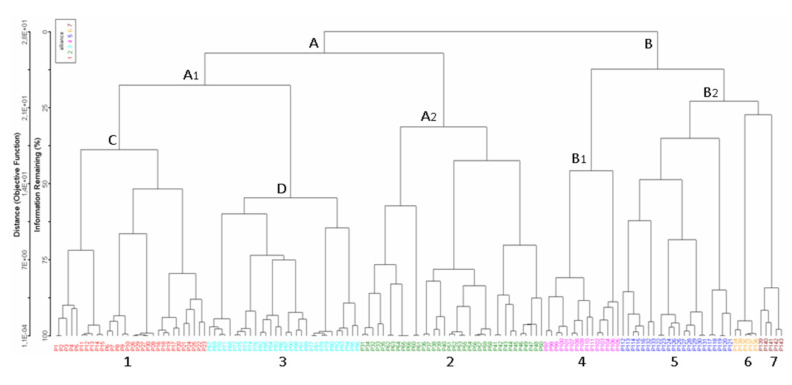
Dendrogram resulting from the cluster analysis of the data set; different colours correspond to different alliances: **1**. *Isöetion*; **2**. *Preslion cervinae*; **3**. *Cicendio-Solenopsion laurentiae*; **4**. *Lythrion tribracteati*; **5**. *Verbenion supinae*; **6**. *Nanocyperion flavescentis*; **7**. *Agrostion salmanticae*.

The Optimclass diagram shows a peak of faithful species at 30 partitions of the dataset. Additionally, compared with the Optimclass diagrams obtained by other clustering methods (e.g., flexible beta, Euclidean distance; UPGMA, Bray-Curtis; UPGMA, Euclidean), the optimal partitions turn out to range from 28 to 34. The Crispness of Classification indicates the clearest separations between two and seven clusters: at two clusters the two orders *Isöetalia* and *Nanocyperetalia* result separated (except for *Agrostion salmanticae*), while the partition at seven groups identifies the seven alliances. According to the Optimclass analysis, the dendrogram was pruned at the level of 32 clusters of relevés. The groups thus identified correspond to the surveyed associations, wholly autonomous from a floristic and ecological point of view and quite well differentiated from each other. Overall, 32 associations, seven alliances and two orders were recognized. On the whole, from the multivariate analysis, the associations identified were well differentiated from each other and were arranged in distinct clusters. Indeed, as shown in Figure 6, two main clusters can be detected, which are separated into ecological groups.

The first to disjoin is cluster A, including the associations referred to as *Isoëtetalia*, with a winter-spring cycle, linked to periodically flooded surfaces drying up from late spring to early autumn. This cluster splits into two main sub-clusters; of these, the first to separate is that one corresponding to A1, which includes communities subject to shorter periods of flooding, while the second is A2, concerning associations with soils submerged for longer periods, often until late spring, including exclusively those of *Preslion cervinae*. In the case of sub-cluster A1, the associations of two different alliances are here arranged, such as the *Isoëtion* (group C) and the *Cicendio-Solenopsion laurentiae* (group D), which differ mainly in the type of habitat in which they are localized. As regards cluster B, also within it there are two main sub-clusters, both corresponding to communities with a summer-autumn cycle. The most isolated one is indicated with B1, which concerns the associations of *Lythrion tribracteati* linked to loamy-clayey and nutrient-rich soils, while in B2 there are the associations of *Verbenion supinae*, typical of more or less nitrified soils and those of the *Nanocyperion* limited to hyper-humid and nutrient-poor soils. Unfortunately, the vegetation of the *Agrostion salmanticae* falls within the sub-cluster B2, together with that of the *Nanocyperion*. This arrangement is totally in contrast with the syntaxonomical framing since from the floristic and ecological point of view it belongs to the *Isoëtetalia.* This is probably due to the quite floristic poor set of this community, which does not allow clustering analysis to find a correct place in the dendrogram. Concerning the ecology of this vegetation, it is localized in large hollows, mainly represented by dolines, usually used as pastures, with well nitrified soils. Results of the DCA (Detrended Correspondence Analysis) ordination approximately confirm the general pattern highlighted by the cluster analysis. The eigenvalues decrease progressively from the first to the third axis (0.82, 0.49 and 0.36, respectively; higher eigenvalues are related to higher beta diversities).

**Figure 7:**
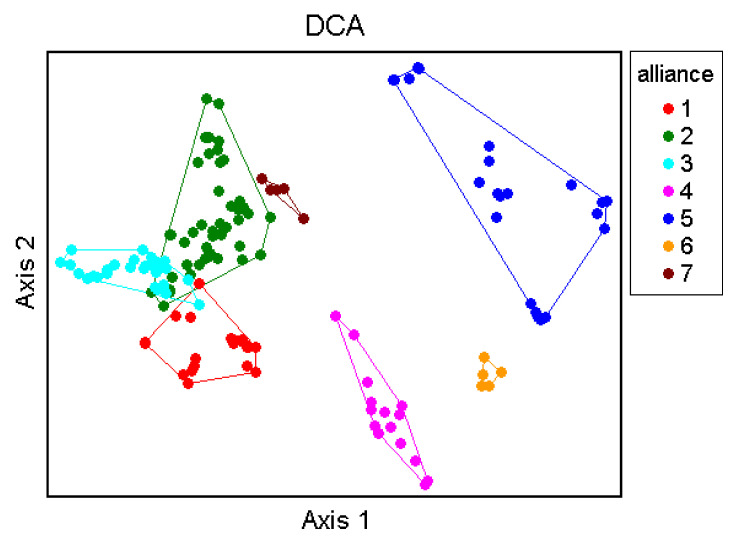
DCA ordination (axis 1 and 2) plot of the data set, with single relevés marked according to different alliances: **1**. *Isöetion*; **2**. *Preslion cervinae*; **3**. *Cicendio-Solenopsion laurentiae*; **4**. *Lythrion tribracteati*; **5**. *Verbenion supinae*; **6**. *Nanocyperion flavescentis*; **7**. *Agrostion salmanticae*.

**Figure 8:**
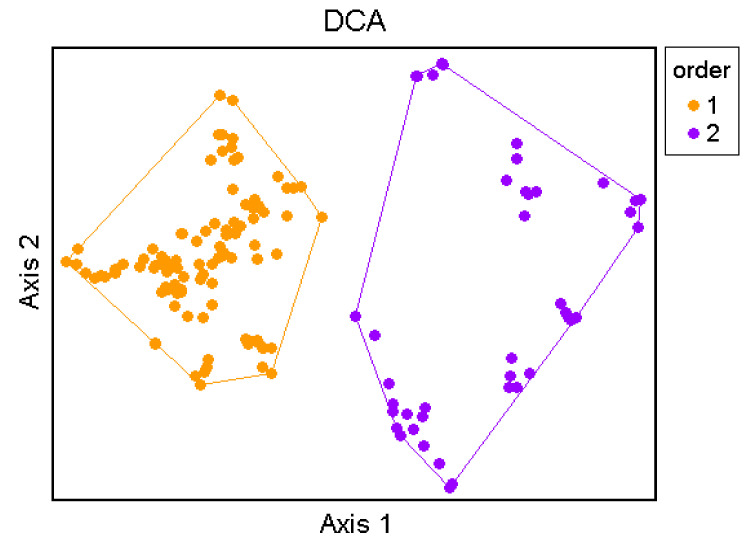
DCA ordination (axis 1 and 2) plot of the data set, with single relevés marked according to orders: **1**. *Isöetalia*; **2**. *Nanocyperetalia*.

Figure 7 shows the ordination results along axis 1 and 2. On the left side of the DCA graph, the *Isoëtetalia* alliances (*Agrostion pourretii* included), are well separated from those ones of *Nanocyperetalia* that are distributed on the right side. Moreover, Figure 8 displays the relevés distributed based on the orders.

### 2.2. Syntaxonomical Scheme of Isoëto-Nanojuncetea in Sicily

According to the multivariate analysis based on the phytosociological relevés used for this investigation, and considering of the arrangements proposed from the aforesaid authors, the plant communities occurring in the study area can be framed in the following syntaxonomical scheme:

ISOËTO-NANOJUNCETEA Br.-Bl. & R. Tx. ex Westhoff, Dijk & Passchier 1946
 ISOËTETALIA Br.-Bl. 1936 nom. conserv. propos.
  *ISOËTION Br.-Bl. 1936*
   *Isoëtetum durieui* Br.-Bl. 1936
   *Pulicario-Scirpetum savii* Brullo & Di Martino 1974
   *Isoëto-Ranunculetum parviflori* Brullo, Di Martino & Marcenò 1977
    subass. *isoëtetosum durieui* Brullo, Di Martino & Marcenò 1977
    subass. *callitrichetosum brutiae* Brullo, Di Martino & Marcenò 1977
   *Crassulo-Elatinetum gussonei* Bartolo, Brullo, Minissale & Spampinato 1990
   *Lythro hyssopifoliae-Elatinetum macropodae* Brullo, Sciandrello, Tavilla & Minissale ass. nov.
    subass. *typicum*
    subass. *buillardietosum vaillantii* subass. nov.
    subass. *callitrichetosum brutiae* subass. nov.
   *Buillardio vaillantii-Elatinetum campylospermae* Brullo, Sciandrello, Minissale, Cambria, Ilardi & Giusso ass. nov.
   *Isoëtetum todaroanae* Brullo & Ilardi ass. nov.
  *PRESLION CERVINAE Br.-Bl. ex Moor 1937*
   *Isoëto velatae-Crassuletum vaillantii* Poiron & Barbero 1965
    subass. *typicum*
    subass. *ranunculetosum ophioglossifolii* Brullo, Minissale, Sciandrello & Tavilla subass. nov.
   *Ranunculo lateriflori-Antinorietum insularis* Brullo, Grillo & Terrasi 1976
   *Myosuro minimi-Ranunculetum lateriflori* Raimondo 1980
   *Ranunculetum pratensi-lateriflori* Brullo, C. Brullo & Giusso ass. nov.
   *Ranunculo lateriflori-Callitrichetum brutiae* Brullo & Minissale 1998
   *Callitricho brutiae-Crassuletum vaillantii* Brullo, Scelsi, Siracusa & Tomaselli 1998
   *Junco pygmaei-Pilularietum minutae* Minissale, Molina & Sciandrello 2017
    subass. *typicum*
    subass. *tillaetosum vaillantii* Minissale, Molina & Sciandrello subass. nov.
   *Pilulario minutae-Myosotidetum siculae* Brullo, Cambria, Ilardi & Minissale ass. nov.
   *CICENDIO-SOLENOPSION LAURENTIAE* Brullo & Minissale 1998
   *Archidio phascoidis-Isoëtetum velatae* Brullo & Minissale 1998
   *Anagallido parviflorae-Molinerielletum minutae* Brullo, Scelsi, Siracusa & Tomaselli 1998
    subass. *typicum*
    subass. *crassuletosum vaillantii* Brullo, Scelsi, Siracusa & Tomaselli 1998
   *Kickxio cirrhosae-Solenopsietum gasparrinii* Brullo & Minissale 1998 corr.
   *Solenopsietum mothianae* Brullo, Giusso, Minissale & Sciandrello ass. nov.
   *Solenopsio gasparrinii-Isoëtetum siculae* Brullo, Cambria, Ilardi & Minissale ass. nov.
   *Myosotido congestae- Isoëtetum histricis* Azzaro & Cambria ass. nov.
  *AGROSTION SALMANTICAE* Rivas Goday 1958
   *Trifolio micheliani-Agrostidetum pourretii* Cambria & Brullo ass. nov.
  NANOCYPERETALIA Klika 1935 nom. cons. propos.
   *NANOCYPERION FLAVESCENTIS* Koch 1926
   *Plantagini intermediae-Cyperetum fusci* Sciandrello, D’Agostino & Minissale 2013
  *VERBENION SUPINAE* Slavnic 1951
   *Gnaphalio luteoalbi-Verbenetum supinae* Rivas Goday 1970
   *Heliotropio supini-Heleochloetum schoenoidis* Rivas Goday 1956
   *Glino lotoidis-Verbenetum supinae* Rivas Goday 1964
   *Coronopo squamati-Sisymbrelletum dentatae* Minissale & Spampinato 1987
   *Heleochloo schoenidis-Chenopodietum botryoidis* Brullo & Sciandrello 2006
   *Coronopo squamati-Corrigioletum litoralis* Brullo & C. Brullo ass. nov.
  *LYTHRION TRIBRACTEATI* Rivas Goday & Rivas-Mart. ex Rivas Goday 1970
   *Damasonio bourgaei-Crypsietum aculeatae* Rivas-Martínez & Costa in Rivas-Martínez *et al.* 1980 corr. V. Silva & J.C. Costa in Costa et al., 2012
   *Ranunculo trilobi-Lythretum tribracteati* Brullo & Sciandrello ass. nov.
   *Pulicario grecae-Damasonietum bourgaei* Minissale, Santo & Sciandrello 2011

For each of these syntaxa the nomenclatural, floristic, ecological and chorological characteristics are analyzed, as can be deduced from the literature data and unpublished field observations.

### 2.3. Description of the Vegetation

*ISOËTO-NANOJUNCETEA* Br.-Bl. & R. Tx. ex Westhoff, Dijk & Passchier 1946, Overz. Plantegem. Neder. 2.:39.

Syn.: *Isoëto-Nanojuncetea* Br.-Bl. & R. Tx. 1943, Comm. S.I.G.M.A. 84: 7, nom. inval. (art. 2b, 8); *Isoëto-Nanojuncetea* Br.-Bl. & R. Tx. in Br.-Bl. et al. 1952, Group. Vég. Fr. Médit.: 80, nom. illeg. (art. 31); *Isoëtetea velatae* de Foucault 1988, Dissert. Bot. 121: 73; *Juncetea bufonii* de Foucault 1988, Dissert. Bot. 121: 78.

Lectotypus: *Isoëtetalia* Br.-Bl. 1936 nom. cons. propos.

Characteristic species: *Damasonium bourgaei*, *Eryngium pusillum*, *Gaudinia fragilis*, *Juncus bufonius*, *J. capitatus*, *J. hybridus*, *J. pygmaeus*, *Lythrum hyssopifolia*, *Mentha pulegium*, *Myosurus minimus*, *Poa infirma*, *Polypogon subspathaceus*, *Pulicaria vulgaris* var. *vulgaris*, *Ranunculus sardous*, *Veronica anagalloides*.

Structure and ecology: Ephemeral amphibious vegetation occurring in temporary wetlands with soils periodically flooded by oligotrophic, mesotrophic, eutrophic, or sometimes brackish waters [39,113,114]. Floristically, these plant communities are dominated by hygrophilous therophytes often mixed with small hemicryptophytes and geophytes. These phytocoenoses correspond to a type of ephemeral vegetation, linked to very peculiar habitats characterized by a temporary submersion alternating with marked aridity, which in the absence of environmental alterations do not tend to evolve; therefore, they can be considered as communities representing ’permaseries’ of vegetation. The associations of this class usually fall in the habitat of Community interest 3170*, which is considered of priority importance [115].

Geographical distribution: The associations of this class are widespread in Europe and all Mediterranean territories, including the Macaronesian islands.

*ISOËTETALIA* Br.-Bl. 1936, Bull. Soc. Et. Sci. Nat. Nimes, 47: 142 nom. cons. propos [116].

Syn.: *Isoëtetalia* Br.-Bl. 1931, Comm. S.I.G.M.A. 9: 38, nom. nud. (art. 2b); *Isoëtetalia velatae* de Foucault 1988, Dissert. Bot. 121: 73.

Type: *Isoëtion* Br.-Bl. 1936 conserved type proposed [116] 

Characteristic species: *Archidium alternifolium*, *Briza minor*, *Bulliarda vaillantii*, *Catabrosa aquatica*, *Centaurium maritimum*, *Damasonium polyspermum*, *Elatine macropoda*, *Isoëtes longissima*, *I. sicula*, *Isolepis cernua*, *Lotus angustissimus*, *L. hispidus*, *L. parviflorus*, *Middendorfia borysthenica*, *Molineriella minuta*, *Myosotis sicula*, *Ranunculus muricatus*, *Romulea ramiflora*, *Trifolium micranthum*, *Triglochin laxiflora*. 

Structure and ecology: Pioneer ephemeral vegetation with thermophilous or subthermophilous requirements linked mainly to oligotrophic soils submerged up to early spring, sometimes flooded until early summer [39,113]. Usually, it is characterized by hygrophilous microphytes having an early spring blooming.

Geographical distribution: This order shows a Mediterranean and South Atlantic-European distribution.

Note: According to Fernández-González et al. [116] *Isoëtetalia* Br-Bl. 1936 is a superfluous name (Art. 29c), and homotypic of *Nanocyperetalia* Klika 1935, having a nomenclature type *Nanocyperion flavescentis* Koch 1926 (Art. 18b). The authors, to avoid publishing a new name for *Isoëtetalia*, propose to use Art. 53 of the new ICPN [117], which allows the preservation of its common use, proposing it as nomen conservandum having the *Isoëtion* Br.-Bl. 1936 as conserved type.

*ISOËTION* Br.-Bl. 1936, Bull. Soc. Et. Sci. Nat. Nimes 47: 141.

Syn.: *Isoëtion* Br.-BI. 1931, Comm. S.I.G.M.A. 9: 38. nom. nud. (art. 2b); *Antinorio agrostideae- Isoëtion velatae* de Foucault 1988 Dissert. Bot. 121: 73, p.p.; *Ophioglosso lusitanici- Isoëtion histricis* de Foucault 1988, Dissert. Bot. 121: 74; *Elatino-Damasonion alismae* de FoucauIt 1988, Dissert. Bot. 121: 86, p.p.; *Crassulo-Lythrion borysthenici* de Foucault 1988, Dissert. Bot. 121: 90 p.p.;

Lectotypus: *Isoëtetum duriei* Br.-Bl. 1936

Characteristic species: *Isoëtes durieui*, *I. histrix*, *Lotus conimbricensis*, *Ranunculus trilobus*.

Structure and ecology: Pioneer and fleeting vegetation localized mainly in small ponds with shallow waters, rich in quillworts and microphytes, showing an early spring blooming, linked to warm Mediterranean climates. It colonizes small surfaces represented by rocky pools with very thin silty soils that dry up very early.

**Figure 9:**
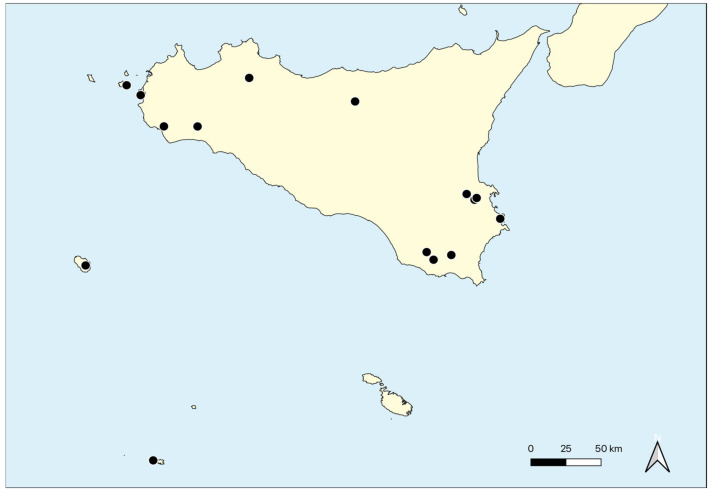
Geographical distribution of the *Isoëtion* associations in Sicily.

Geographical distribution: This alliance has a Mediterranean distribution and in Sicily, it occurs in the several localities of the Islands, as well as Pantelleria Island, Lampedusa Island, Favignana Island and Isola Grande dello Stagnone (Figure 9).

**1.**  *Isoëtetum durieui* Br.-Bl. 1936, Bull. Soc. Étude Sci. Nat. Nîmes 47: 144 (Appendix B, Table A2)

Syn.: Ass. à *Juncus capitatus* - *Isoëtes duriaei* Br.-Bl. 1931, Comm. SIGMA 9 : 38 nom. nud. (art. 2b).

Lectotypus: rel. 3 Tab. « *Isoëtetum duriaei* », Braun-Blanquet [5]

Characteristic species: *Isoëtes durieui*, *Juncus capitatus*, *Trifolium micranthum*.

Structure and ecology: This association was described in southern France by Braun-Blanquet [5] on siliceous substrata where it is localized on small rocky hollows submerged in the winter season. Floristically it is characterized by the dominance of *Juncus capitatus* and *Isoëtes durieui*, which grow together with other hygrophilous microphytes. In Sicily, the association occurs in the slightly depressed soils rich in silt and clay on siliceous substrata, where it is characterized by a very poor floristic set.

Geographical distribution: Previously, the *Isoëtetum durieui* was recorded from southern France [5,8,15], Catalonia in Spain [118,119,120,121,122] and Corse [123]. In Sicily, the plant communities referable to this association are very rare. In particular, *Isoëtetum durieui* was quoted by Marcenò & Trapani [85] from Piana degli Albanesi (Palermo); it occurs also in the Hyblean plateau near Carlentini.

**2.**  *Pulicario graecae-Scirpetum savii* Brullo & Di Martino 1974, Boll. Ist. Bot. Giard. Coll. Palermo 26: 49 (Appendix B, Table A3)

Lectotypus: rel. 10, Tab. 17, Brullo and Di Martino [78], hoc loco.

Characteristic species: *Isolepis cernua (= Scirpus savii)*, *Pulicaria vulgaris* var. *graeca*, *Damasonium polyspermum*, *Damasonium bourgaei (sub D. stellatum)*. 

Structure and ecology: The association was surveyed in the rocky ponds of the arenaceous outcrops submerged by rainwater during the winter. At the bottom of these small depressions, a thin layer of silt is deposited, where peculiar amphibious hygrophilous vegetation grows. In this habitat, the occurrence of some hygrophytes, such as *Isolepis cernua*, *Pulicaria vulgaris* var. *graeca*, *Damasonium polyspermum*, *Damasonium bourgaei* is quite significant. These species, proposed as characteristic, are usually mixed with several other hygrophytes of *Isoëto-Nanojuncetea*. The association colonizes small surfaces within the perennial vegetation belonging to *Sarcocornio fruticosae-Limonietum ferulacei juncetosum subulati*, a hyper-halophilous association of *Salicornietea fruticosae* [78].

Geographical distribution: Currently, it is known only in the Isola Grande dello Stagnone (Trapani).

**3.**  *Isoëto durieui-Ranunculetum parviflori* Brullo, Di Martino & Marcenò 1977, Veg. Pantelleria: 82 (Appendix B, Table A4)

Holotypus: rel. 8. Tab. 12, Brullo et al. [81].

Characteristic species: *Isoëtes durieui*, *Ranunculus parviflorus*.

Structure and ecology: The small, periodically flooded hollows on basaltic rocks, localized at about 600 m a.s.l., are colonized by hygrophilous vegetation dominated by *Isoëtes durieui* and *Ranunculus parviflorus*, which were proposed as characteristic species of a peculiar association named *Isoëto-Ranunculetum parviflori* [81]. In this vegetation, several species of *Isoëto-Nanojuncetea* are frequent, such as *Ranunculus trilobus*, *R. muricatus*, *Juncus bufonius*, *Lythrum hyssopifolia*, *Lotus angustissimus*, *Mentha pulegium*, etc. Besides, two sub-associations can be identified within it: *isoëtetosum durieui* corresponding to the typical aspect and *callitrichetosum brutiae*, linked to conditions of greater edaphic humidity occurring in the central part of the depressions.

Geographical distribution: It is a very rare association exclusive of Pantelleria Island, where it is localized at Monte Gibele on the bottom of a volcanic crater.

**4.**  *Crassulo vaillantii-Elatinetum gussonei* Bartolo, Brullo, Minissale & Spampinato 1990, Boll. Acc. Gioenia Sci. Nat. Catania 21 (334): 205 (Appendix B, Table A5)

Holotypus: rel 6, Tab. 25 Bartolo et al. [88].

Characteristic species: *Elatine gussonei*.

Structure and ecology: The association occurs in the rocky ponds circumscribed to the carbonatic outcrops submerged by freshwater during the autumn-winter period. These peculiar habitats are characterized by a thin layer of soil covered by 5–10 cm of water at the time of maximum flooding. Floristically, this vegetation is differentiated by the dominance of *Elatine gussonei*, endemic to Lampedusa and Maltese islands [124,125], which is related to *Elatine macropoda.* It usually grows together with *Buillardia vaillantii* and other hygrophytes of *Isoëto-Nanojuncetea.* As concerns its bioclimatic requirements, this plant community grows within the upper Infra-Mediterranean belt, with lower semiarid hombrotype [96]. The climatophilous vegetation where it falls is represented by the thermo-xeric maquis belonging to the *Periplocion angustifoliae* Rivas-Martinez 1975 [88].

Geographical distribution: According to Bartolo et al. [88] and Brullo et al. [126], the *Crassulo vaillantii-Elatinetum gussonei* is distributed in Lampedusa (Pelagie Islands) and Maltese Islands (Figure 2F).

**5.**  *Lythro hyssopifoliae-Elatinetum macropodae* Brullo, Sciandrello, Tavilla & Minissale ass. nova hoc loco (Appendix B, Table A6)

Holotypus: rel 10, hoc loco.

Characteristic species: *Elatine macropoda.*

Structure and ecology: This vegetation grows in small and not very deep wet hollows, represented by cupular pools, occurring in limestone plateaus, which start to dry up at the beginning of spring. The relevant occurrence in this phytocoenosis of *Elatine macropoda*, species widespread in the Mediterranean territories, often shows high coverage values. The set of *Isoëto-Nanojuncetea* microphytes is well represented, such as *Juncus bufonius*, *Buillardia vaillanti*, *Lythrum hissopifolia*, *Mentha pulegium*, *Poa infirma*, etc. This community, due to the dominance of *Elatine macropoda*, shows some relationship with associations described for other Mediterranean territories, for instance, *Elatinetum macropodae* Br.-Bl. 1936 from south France and *Junco pygmaei-Elatinetum macropodae* Silva et al., 2021 from the Iberian Peninsula. However, the vegetation is floristically and ecologically well differentiated from these two associations. In particular, *Elatinetum macropodae* colonizes the basaltic rocky pools and is characterized, apart from *Elatine macropoda*, by *Damasonium polyspermum*, *Herniaria glabra*, *Pulicaria vulgaris* and *Lythrum tribracteatum*, which are species fully absent in the Sicilian community, while *Junco pygmaei-Elatinetum macropodae* is localized along the edge of streams or temporary ponds with muddy-sandy substrates and is differentiated by the occurrences of *Juncus pygmaeus* and *Middendorfia borysthenica* growing together with *Elatine macropoda*. As a whole, both these associations show marked differences in comparison with the vegetation surveyed in Sicily. In fact, the latter is floristically differentiated by the occurrence of *Buillardia vaillantii*, showing high cover values, which is lacking in the other two aforesaid associations. Therefore, this Sicilian plant community is here proposed as a new association, named *Lythro hyssopifoliae-Elatinetum macropodae*. It should be noted that this new association is quite similar to *Crassulo vaillantii-Elatinetum gussonei* from Lampedusa, mainly for the habitat where it grows and also for the occurrence of *Buillardia vaillantii*, while *Elatine macropoda* is replaced by *Elatine gussonei.* Within this association three subassociations can be distinguished, namely: (a) subass. *typicum* (rel. 10–20) differentiated by the dominance of *Elatine macropoda*, linked to cupular pools flooded by shallow waters; (b) subass. *buillardietosum vaillantii* subass. nov. (rel. 1–9, holotypus rel. 8, hoc loco) localized in the stands with quite deep waters; (c) subass. *callitrichetosum brutiae* subass. nov. (rel. 21–25, holotypus rel. 24, hoc loco) occurring exclusively on stands with very deep waters.

Geographical distribution: This association to the best of current knowledge is exclusive of the Hyblean basaltic plateau in southern Sicily (Figure 2E and Figure 4B).

**6.**  *Buillardio vaillantii-Elatinetum campylospermae* Brullo, Sciandrello, Minissale, Cambria, Ilardi & Giusso ass. nov., hoc loco (Appendix B, Table A7)

Syn.: *Elatinetum macropodae* Pasta et al. 2008, Nat. Sicil. ser. 4, 32 (1–2): 41, non Br.-Bl. 1936, Bull. Soc. Et. Sci. Nat. Nimes 47:154.

Holotypus: rel 14, hoc loco.

Characteristic species: *Elatine campylosperma.*

Structure and ecology: The small pools between limestone outcrops, both of natural and anthropogenic origin, usually named rocky pools, flooded in the autumn-winter period, and host highly specialized amphibious plant communities. In these stands, *Bulliarda vaillantii* seems to have here its optimum, growing together with various other ephemeral hygrophytes, such as *Lythrum hyssopifolia*, *Juncus bufonius*, *Poa infirma*, *Juncus capitatus*, *Juncus hybridus*, *Polypogon subspathaceus*, etc. The occurrence of a very peculiar species of *Elatine*, was quite significant; according to current knowledge of this genus, it can be attributed to *E. campylosperma*, a Mediterranean species with a very scattered distribution [127]. From the phytosociological point of view, this vegetation shows a close relationship with other Sicilian associations dominated by *Elatine* sp. and *Bulliarda vaillantii*, such as *Lythro hyssopifoliae-Elatinetum macropodae* and *Crassulo vaillantii-Elatinetum gussonei*, occurring in quite similar habitat, but differing among them from the floristic point of view, since characterized by other species of *Elatine*. Therefore, the plant community at issue is proposed as a new association named *Buillardio vaillantii-Elatinetum campylospermae*. Previously, this vegetation was wrongly attributed by Pasta et al. [128] to *Elatinetum macropodae* Br. Bl. 1936.

Geographical distribution: The association is localized in the North-western Sicily, where it occurs in some localities of the Trapani territory, such as Castello della Pietra (Castelvetrano), Isola Lunga dello Stagnone (Marsala) and Favignana island (Aegadian islands) (Figure 2B, Figure 4A and Figure 5E).

**7.**  *Isoëtetum todaroanae* Brullo & Ilardi ass. nova hoc loco (Appendix B, Table A8)

Holotypus: rel. 1, hoc loco.

Characteristic species: *Isoëtes todaroana*.

Structure and ecology: A rather rare and very peculiar community has been surveyed in small hollows on calcarenitic outcrops, submerged especially during the winter period. In this habitat *Isoëtes todaroana*, a peculiar species described by Troia & Raimondo [129], is localized. It grows on a thin layer of clay soil together with other hygrophytes of the *Isoëto-Nanojuncetea* including, in particular: *Triglochin laxiflora*, *Romulea ramiflora*, *Isolepis cernua*, *Mentha pulegium*, *Lythrum hyssopifolia*, *Juncus bufonius.* The temporary wetland where this vegetation currently occurs is a remaining fragment of a larger marsh that has been reclaimed in a cultivated area, and therefore, it takes on a relict meaning. Hence, this phytocoenosis for its floristic and ecological features is proposed as a new association named *Isoëtetum todaroanae*.

Geographical distribution: Currently, this association seems to have a punctiform distribution localizing in a small area near Mazara del Vallo (Trapani).

*PRESLION CERVINAE* Br.-Bl. ex Moor 1937, Prodr. Group. Veg. 4: 22.

Syn.: *Preslion* Br.-Bl. 1931, Comm. S.I.G.M.A.: 38, nom. nud. (art. 2b); *Menthion cervinae* Br.-Bl. ex Moor 1936, nom. mut. propos. by Rivas-Martínez et al. (2002); *Elatino-Damasonion alismae* de FoucauIt 1988, Dissert. Bot. 121: 86, p.p.

Holotypus: *Preslietum cervinae* Br.-Bl. ex Moor 1937.

Characteristic species: *Antinoria insularis*, *Callitriche brutia*, *Juncus foliosus*, *Pilularia minuta*, *Ranunculus lateriflorus*, *R. ophioglossifolius*, *R. pratensis*, *R. saniculifolius*, *Veronica serpyllifolia*.

Structure and ecology: Thermophilous plant communities localized in cupular pools, temporary marshes and dolines with deep stagnant waters or in stands with deep-water runoff flooded for most of the spring. This vegetation is rich in creeping amphibian species mixed with hygrophilous microphytes.

**Figure 10:**
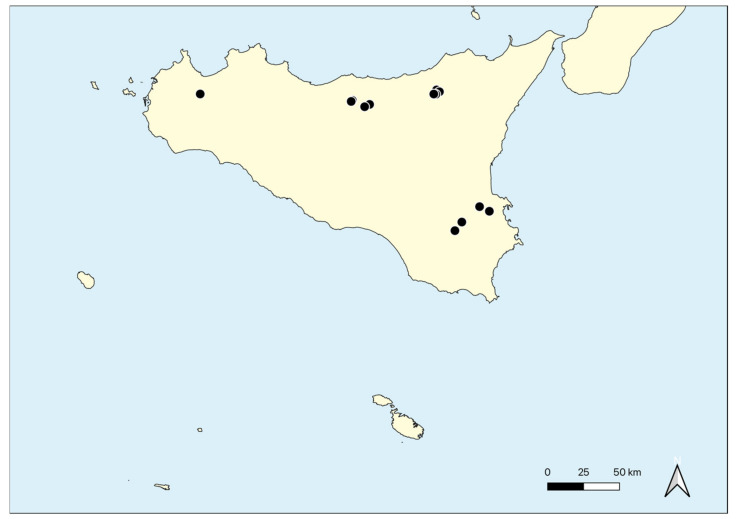
Geographical distribution of the *Preslion cervinae* associations in Sicily.

Geographical distribution: This alliance shows a Mediterranean range and in Sicily, it occurs in some localities of the northern and southern parts of the Island (Figure 10).

**8.**  *Isoëto velatae-Crassuletum vaillantii* Poiron & Barbero 1965, Bull. Soc. Bot. Fr., 112: 437 (Appendix B, Table A9)

Syn.: Association à *Isoëtes velata* et *Crassula vaillantii* Poiron & Barbero 1965, Bull. Soc. Bot. Fr. 112: 437

Lectotypus: rel. 9, Tab. pag. 439, Poiron & Barbero [130], hoc loco.

Characteristic species: *Crassula vaillantii*, *Isoëtes velata*, *Warnstorfia fluitans (= Drepanocladus fluitans)*.

Structure and ecology: The small rocky pools occurring on the basaltic plateau submerged in the rainy periods with very shallow waters persisting until the early spring, host a hygrophylous vegetation characterized by the dominance of *Isoëtes velata* and *Crassula vaillantii*, to which *Warnstorfia fluitans*, a rare moss recently recorded from Sicily [131], is often associated. It is a silicicolous community localized at 230–360 m a.s.l., that, according to Minissale et al. [132], can be referred to as *Isoëto velatae-Crassuletum vaillantii*, an association described in southern France by Poiron & Barbero [130]. The Sicilian vegetation shows a floristic set and ecological requirements quite similar to the one surveyed in France. Apart from the subass. *typicum* (rel. 1–12), in Sicily, is possible to distinguish a subass. *ranunculetosum ophioglossifolii* Brullo, Minissale, Sciandrello & Tavilla subass. nov. (rel. 13–16, holotypus rel. 13, hoc loco) occurring in the stands with a longer flooding period, floristically differentiated by *Ranunculus ophioglossifolius* and *Warnstorfia fluitans*.

Geographical distribution: According to literature data, this association considered circumscribed to France and Italy [51,77,130], occurs also in the Hyblean Plateau (southern Sicily), as already mentioned by Minissale et al. [132].

**9.**  *Ranunculo lateriflori-Antinorietum insularis* Brullo, Grillo & Terrasi 1976, Boll. Acc. Gioenia Sci. Nat. Catania, 12: 92 (Appendix B, Table A10).

Lectotypus: rel. 11, Tab. 3, Brullo et al. [80], hoc loco.

Characteristic species: *Bulliarda vaillantii*, *Isoëtes sicula*, *Myosotis tineoi*.

Structure and ecology: In the small wetlands and rocky pools occurring on the basaltic plateau at about 900 m of altitude, peculiar vegetation linked to habitats flooded until the early summer, when deep freshwater occurs. It is characterized by *Antinoria insularis* and *Ranunculus lateriflorus*, usually growing with *Buillardia vaillantii*, *Isoëtes sicula* and *Myosotis tineoi.* This community was described by Brullo et al. [80] as *Ranunculo lateriflori-Antinorietum insularis* and attributed, even if doubtfully, to *Isoëtion.* Effectively, due to the high frequency of *Antinoria insularis*, *Ranunculus lateriflorus* and *Callitriche brutia* this association is to be included within *Preslion cervinae.* Regarding its bioclimatic requirements, the association falls within the meso-Mediterranean subhumid belt. At first, two subassociations were recognized in this plant community, named *isoëtetosum* and *ranunculetosum*, differentiated, respectively, by *Isoëtes sicula*, formerly identified as *I. durieui*, linked to stands subject to a shorter submersion period, while the other one is dominated by *Callitriche brutia*, localizing on longer flooded surfaces. According to Brullo & Minissale [39], they must be considered as two distinct associations. The first one coincides with the association at issue, while the second one was named by Brullo & Minissale [39] *Ranunculo lateriflori-Callitrichetum brutiae*, which will be treated later.

Geographical distribution: This association occurs exclusively on the top of Monte Lauro in the Hyblean Plateau.

**10.**  *Myosuro minimi-Ranunculetum lateriflori* Raimondo 1980, Quaderni C.N.R., AQ/1/89: 15 (Appendix B, Table A11).

Holotypus: rel. 1, Tab. 2, Raimondo [86].

Characteristic species: *Myosurus minimus*, *Spergularia madoniaca*, *Sagina subulata*, *Ranunculus marginatus*

Structure and ecology: The association is localized in mountain stands, at altitudes between 1400 and 1600 m a.s.l., especially on the bottom of large dolines, limitedly to the small depressions where water, resulting from the melting of snow, is stagnant for a long time. The soil is represented by clayey-silty deposits, resulting from the erosion of the surrounding carbonatic rocks, with an acidic pH, usually drying up towards the end of spring. These wet surfaces are colonized by dense vegetation with hygrophilous microphytes, where some of them play a relevant physiognomic role. In particular, *Myosurus minimus*, *Spergularia madoniaca*, *Antinoria insularis*, *Ranunculus lateriflorus*, are the most frequent, which are associated with several other hygrophytes of the *Isoëto-Nanojuncetea.* The association, described by Raimondo [86] as *Myosuro minimi-Ranunculetum lateriflori*, was included in the *Isoëtion*, while Brullo & Minissale [39] put it in synonymy with *Ranunculo lateriflori-Antinorietum insularis.* Based on the current knowledge this vegetation is more advisable to be kept as an autonomous association, closely related to the latter. As previously emphasized by Brullo et al. [84], both for their localization in the mountain belt and for the floristic set, mainly due to the occurrence of *Ranunculus lateriflorus*, *Myosuro-Ranunculetum lateriflori* and also *Ranunculo-Antinorietum insularis* can be considered as geographical vicariants of other allied associations, such as *Sedo nevadensis-Juncetum pygmaeis* Quezel 1957 from Atlas range (North Africa), *Junco-Isoëtetum velatae* Rivas Goday 1955 from Spain, and *Veronico-Ranunculetum lateriflori* Quezel 1973 from Tauro massif (Turkey).

Geographical distribution: The association is localized in the Madonie massif (North Sicily) (Figure 3A).

**11.**  *Ranunculetum pratensi-lateriflori* Brullo, C. Brullo & Giusso ass. nova hoc loco (Appendix B, Table A12).

Syn.: *Ranunculo-Antinorietum insularis veronicetosum* Brullo & Grillo 1978, Not. Soc. Ital. Fitosociol. 13: 45.

Holotypus: rel. 5, Tab. 7, Brullo & Grillo [84], hoc loco.

Characteristic species: *Ranunculus pratensis*, *Barbarea bracteosa*, *Veronica serpyllifolia*.

Structure and ecology: In the mountain range of northern Sicily, at altitudes between 1300 and 1700 m a.s.l., in correspondence with wetlands periodically flooded by shallow waters; ephemeral hygrophilous vegetation with a typical spring cycle. The substrates consist of siliceous rocks, mainly represented by flysch and schists, covered by silty-clayey deposits. The vegetation colonizing these places is characterized by quite specialized hygrophytes, where a relevant physiognomic role is played by *Antinoria insularis* and *Ranunculus lateriflorus*, as well as by *R. pratensis*, *Veronica serpyllifolia*, *Barbarea bracteosa*, which allow differentiating a new association, closely related to the *Ranunculo-Antinorietum insularis.* Previously Brullo & Grillo [84], treated this plant community as a subass. *veronicetosum* of the last association. Nevertheless, apart from the different floristic sets, it is well diversified also from the ecological point of view, since it is distributed at higher altitudes, well over 1000 m a.s.l., and on different substrates. Therefore, it is proposed as *Ranunculetum pratensi-lateriflori*, which is spread mainly in the supra-Mediterranean humid belt.

Geographical distribution: The association is recorded from the Nebrodi chain (northern Sicily).

**12.**  *Ranunculo lateriflori-Callitrichetum brutiae* Brullo & Minissale 1998, Itinera Geobot. 11: 281 (Appendix B, Table A13).

Syn.: *Ranunculo-antinorietum insularis* subass. *ranunculetosum* Brullo, Grillo & Terrasi 1976, Boll. Acc. Gioenia Sci. Nat. Catania, 12: 93.

Holotypus: rel. 21, Tab. 3, Brullo et al. [80]

Characteristic species: *Callitriche brutia.*

Structure and ecology: This vegetation occurs in the pools with more or less deep waters, limited to the basaltic substrates covered by a thin clayey-loamy soil layer. From a floristic point of view, this vegetation is characterized by the dominance of *Callitriche brutia*, *Ranunculus lateriflorus* and *Bulliarda vaillantii*. This association, described by Brullo & Minissale [39], was treated by Brullo et al. [80] as subass. *ranunculetosum* of the *Ranunculo-Antinorietum insularis*, and by Brullo et al. [83] as subass. *ranunculetosum lateriflori* of the *Callitricho-Crassuletum vaillantii*; in either case, it represents a more hygrophilous variant of these associations.

Geographical distribution: The association is currently recorded only from Monte Lauro (Hyblean plateau).

**13.**  *Callitricho brutiae-Crassuletum vaillantii* Brullo, Scelsi, Siracusa & Tomaselli 1998, Boll. Acc. Gioenia Sci. Nat. Catania 29: 172 (Appendix B, Table A14).

Holotypus: rel. 1, Tab. 2, Brullo et al. [83]. 

Characteristic species: *Callitriche brutia.*

Structure and ecology: In the deeper depressions of the basaltic substrata, often submerged until the end of springtime, *Anagallido parviflorae-Molinerielletum minutae* is replaced by *Callitricho brutiae-Crassuletum vaillantii*, an association with more hygrophilous requirements. Floristically, this vegetation is characterized by the dominance of *Callitriche brutia* and *Bulliarda vaillantii*, which grows together with other hygrophilous species of the *Isoëto-Nanojuncetea.* It shows some relations with *Ranunculo lateriflori-Callitrichetum brutiae*, from which it differs in lower hygrophily and absence of *Ranunculus lateriflorus*, while *Coleostephus myconis* occurs, which emphasizes the more xericity of the stands.

Geographical distribution: The association is currently recorded only from Bosco Pisano (Hyblean plateau).

**14.**  *Junco pygmaei-Pilularietum minutae* Minissale, Molina & Sciandrello 2017, Botany Letters 164: 200 (Appendix B, Table A15).

Holotypus: rel. 8, Tab. 1, Minissale et al. [132])

Characteristic species: *Pilularia minuta*

Structure and ecology: This is a very rare association localized on basaltic substrata, where it grows on ponds and drainage ditches, mainly on flat or slightly sloping surfaces with a superficial clayey or clayey-silty soil layer. This vegetation is distributed at 360-400 m a.s.l., within the thermo-Mediterranean bioclimatic belt. Floristically, it is characterized by the occurrence of *Pilularia minuta*, usually growing with some hygrophilous species of *Isoëto-Nanojuncetea*, among them, such as *Juncus pygmaeus*, *Isoëtes longissima*, *Lotus angustissimus*, *Lythrum hyssopifolia*, *M. borysthenica*, *Callitriche brutia*, *Bulliarda vaillantii*, *Mentha pulegium*, etc. Within this association, two subassociation can be distinguished: the *typicum* (rel. 10–14) localized in the natural ponds and differentiated by *Solenopsis laurentia* subsp. *hyblaea* and *Cicendia filiformis*, while the *tillaetosum vaillantii* Minissale, Molina & Sciandrello (rel. 1–9, holotypus rel. 4, hoc loco) grows in the drainage ditches and is characterized by the occurrence of *Buillardia vailantii* [132]. This association shows some relations with other plant communities rich in *Pilularia minuta* described from other Mediterranean territories, which were quoted by Tomaselli et al. [51] too. In particular, among them *Isoetetum setacei* Br-Bl. 1936 from southern France and the Iberian Peninsula can be mentioned, along with *Pilulario minutae-Isoetetum longissimae* Tomaselli et al. 2020 from Apulia, *Eryngio corniculati-Isoetetum velatae* Paradis & Finidori 2005 from Sardinia. On the whole, all these associations are floristically and ecologically well differentiated from *Junco pygmaei-Pilularietum minutae.*

Geographical distribution: Based on the current knowledge, this association is exclusive of a small area of the Hyblean Plateau near Sortino and Carlentini (Figure 2D, Figure 4G and Figure 5C).

**15.**  *Pilulario minutae-Myosotidetum siculae* Brullo, Cambria, Ilardi & Minissale ass. nova hoc loco (Appendix B, Table A16).

Holotypus: rel. 1, hoc loco

Characteristic species: *Pilularia minuta* and *Myosotis sicula*. 

Structure and ecology: The association was surveyed in wide temporary wetlands flooded during the winter-spring period, localized on clayey-siliceous substrates covered by deep muddy soils. This vegetation is distributed about 200 m a.s.l., within the thermo-Mediterranean bioclimatic belt. It is characterized by the occurrence of *Pilularia minuta*, a rare and inconspicuous fern, which was recently rediscovered in Sicily by Troia & Lansdown [133]. This hygrophyte usually grows with other species of the *Isoëto-Nanojuncetea*, such as *Myosotis sicula. Ranunculus ophioglossifolius*, *Isoëtes longissima*, *Lotus parviflorus*, *Elatine macropoda*, etc. For its ecology and floristic set, it is well differentiated from the *Junco pygmaei-Pilularietum minutae*, as well as from the *Pilulario minutae-Isoetetum longissimae*.

Geographical distribution: This association seems localized in Contrada Anguillara near Calatafimi (western Sicily).

*CICENDIO-SOLENOPSION LAURENTIAE* Brullo & Minissale 1998, Itinera Geobot. 11:275.

Syn.: *Cicendion* auct. medit. non Br.-Bl. 1967, Vegetatio 14: 28. 

Holotypus: *Laurentio-Anthocerotetum dichotomi* Br.-Bl. 1936, Bull. Soc. Et. Sci. Nat. Nimes 47: 9.

Characteristic species: *Anagallis minima*, *A. parviflora*, *Cicendia filiformis*, *Kickxia cirrhosa*, *Ophioglossum lusitanicum*, *Radiola linoides*, *Solenopsis laurentia subsp. laurentia.* *S. gasparrinii*, *S. laurentia subsp. hyblaea*.

Structure and ecology: This alliance groups spring communities with acidophilus requirements, markedly more sciaphilous and hygrophilous than those of the *Isoëtion*, with soils remaining humid for long periods. They are localized on waterlogged soils of large hollows with waterproof surfaces, sometimes represented by a wood clearing, covered by a thick layer of clay-silt soil, usually rich in a sandy component. It can be observed also in rocky ponds with sandy soils. In these stands, the hygrophilous microphytes are submerged by shallow rainwater often until late spring. Mucina et al. [114] synonymized this alliance with *Cicendion* (Rivas Goday in Rivas Goday & Borja 1961) Br.-Bl. 1967, and included it within the *Isoëtetalia*. When Rivas Goday & Borja (1961) described this syntaxon, they treated it as *Cicendenion* (sub *Cicendion*), considering it as a suballiance of *Nanocyperion flavescentis* Koch 1926, including within it the *Cicendietum filiformis* Allorge 1922, which represents, therefore, its nomenclature type. Later, Braun-Blanquet [18] raised this syntaxon to the alliance level, including within it the new association *Isoëto velatae-Cicendietum filiformis*. In particular, the *Cicendietum filiformis*, described from northern France is characterized by a floristic set rich in elements of the *Nanocyperetalia* order, such as *Isolepis setacea*, *Juncus tenuis*, *Cyperus flavescens*, *C. fuscus*, *Lythrum portula*, *Spergularia rubra*, *Sagina procumbens*, *Centaurium pulchellum*, *Gnaphalium ulginosum*, *G. luteo-album*, while the floristic elements of *Isoëtetalia* are absent. Besides, it should be noted that from the nomenclatural viewpoint, the *Radiolion linoidis* Pietsch 1973 represents a synonym of the *Cicendion* as emphasized by Brullo & Minissale [39]. In fact, the two syntaxa are floristically and ecologically perfectly overlapping, since either way the associations referred to them (*Cicendietum filiformis* included) have the optimum at last spring to early summer, and occur in the territories with temperate bioclimate. Besides, both are characterized by a peculiar pool of species, such as *Centunculus minimus*, *Radiola linoides*, *Hypericum humifusum*, *Montia minor*, *Chaetonychia cymosa*, and many other of the *Nanocyperetalia* order.

**Figure 11:**
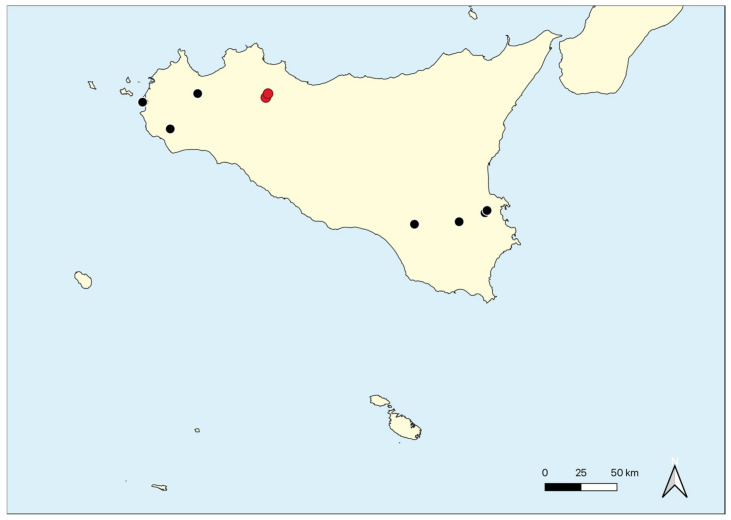
Geographical distribution of the *Cicendio-Solenopsion laurentiae* (black dot) and *Agrostion salmanticae* associations in Sicily (red dot).

Geographical distribution: The alliance is well represented in the western and central Mediterranean area and in Sicily, it occurs in some localities of the western and southern part of the Island (Figure 11).

**16.**  *Archidio phascoidis-Isoetetum velatae* Brullo & Minissale 1998, Itinera Geobot. 11: 281 (Appendix B, Table A17).

Holotypus: rel. pg. 281 related to *Archidio phascoidis-Isoetetum velatae* Brullo & Minissale (1998).

Characteristic species: *Archidium phascoides*, *Isoëtes longissima (= Isoëtes velata)*

Structure and ecology: Hygrophilous vegetation that prefers small humid depressions, on basaltic substrata covered by a shallow silty soil layer, subject to flooding during the autumn-winter period. From a structural point of view, the vegetation is characterized by a low moss carpet dominated by *Archidium phascoides*, on which numerous hygrophilous microphytes grow, such as, *Solenopsis laurentia* subsp. *hyblaea*, *Juncus bufonius*, *J. pygmaeus*, *J. capitatus*, *Anagallis parviflora*, *Centaurium maritimum*, etc. The occurrence of some *Isoëtes*, such as *I. longissima*, *I. durieui* and *I. histrix* is significant. The association is quite rare localizing at 230–385 m a.s.l., within the thermo-Mediterranean sub-humid bioclimatic belt.

Geographical distribution: The association occurs on scattered volcanic rocks in the Hyblean Plateau (Figure 3E and Figure 4C,D).

**17.**  *Anagallido parviflorae-Molinerielletum minutae* Brullo, Scelsi, Siracusa & Tomaselli 1998, Boll. Acc. Gioenia Sci. Nat. Catania 29: 172 (Appendix B, Table A18).

Holotypus: rel. 4, Tab. 1, Brullo et al. [83]

Characteristic species: *Molineriella minuta*

Structure and ecology: Hygrophilous vegetation linked to flat stands with shallow loamy soils deposited on basaltic substrates, subject to short periods of submersion during the autumn and winter months. It is an ephemeral association with a very early vegetative cycle (late winter-early spring), in which a remarkable floristic set occurs, represented mainly by microphytes of the *Isoëto-Nanojuncetea*. Among them, *Isoëtes durieui*, *Anagallis parviflora*, *Lotus conimbricensis*, *L. angustissimus*, *Lythrum hyssopifolia*, *Juncus bufonius*, *J. capitatus*, *J. pygmaeus*, *Polypogon subspathaceus*, *Mentha pulegium*, *Poa infirma*, etc., can be mentioned. Besides, particularly significant is the occurrence of *Molineriella minuta*, a species very rare in Sicily, which is treated as a characteristic species of the association. Within this community, two sub-associations were identified: *typicum*, which occupies poorly wet peripherical places subject to a very short period of submersion, and *crassuletosum vaillantii*, differentiated by the dominance of *Buillardia vaillantii*, which is localized in the central part of the depressions with deeper waters [83].

Geographical distribution: This association is reported from Bosco Pisano near Buccheri (Hyblean plateau).

**18.**  *Kickxio cirrhosae-Solenopsietum gasparrinii* Brullo & Minissale 1998, Itinera Geobot. 11: 281, nom. corr. (Appendix B, Table A19).

Syn.: *Kickxio cirrhosae-Solenopsietum laurentiae* Brullo & Minissa1e 1998, Itinera Geobot. 11: 281; *Laurentio-Juncetum tingitani* Brullo, Scelsi & Siracusa 1994, Boll. Acc. Gioenia Sci. Nat. Catania 27:359, non Rivas Goday & Borja in Rivas Goday 1968, Collect. Bot. 7(2): 1022.

Holotypus: rel. 4, Tab. 11, Brullo et al. [134]

Characteristic species: *Kickxia cirrhosa.*

Structure and ecology: It is a microphytic association growing on muddy-sandy soils, limited to small ponds localized within halophilous meadows belonging to the *Limonio dubii-Lygeetum sparti* Brullo & Di Martino 1974. In this vegetation several elements of the *Cicendio-Solenopsion laurentiae* occur, such as *Kickxia cirrhosa*, *Solenopsis gasparrinii*, *Cicendia filiformis*, *Radiola linoides*, *Ophioglossum lusitanicum*, *Centunculus minimum*, etc., which highlights its acidophilic and markedly hygrophilous requirement. It has its optimum in the spring period, especially in very rainy years. Previously, this vegetation was referred to by Brullo et al. [134] as the *Laurentio-Juncetum tingitani* association described in the Iberian Peninsula by Rivas Goday & Borja [105] and later treated by Brullo & Minissale [39] as a new association named *Kickxio cirrhosae-Solenopsietum laurentiae.* Based on the current knowledge, the name of this association must be corrected in *Kickxio cirrhosae-Solenopsietum gasparrinii*, since *Solenopsis laurentia* is here replaced by *S. gasparrinii.*

Geographical distribution: This association currently is reported only from the Isola Grande dello Stagnone near Marsala.

**19.**  *Solenopsietum mothianae* Brullo, Giusso, Minissale & Sciandrello ass. nova hoc loco (Appendix B, Table A20).

Holotypus: rel. 3, hoc loco

Characteristic species: *Solenopsis mothiana*

Structure and ecology: This association was surveyed on wide wetlands with silty-sandy and slightly brackish soils in stands near the sea. Usually, this vegetation is localized in temporarily flooded surfaces occurring in the large clearings within the maquis and is floristically differentiated by *Solenopsis mothiana*, a rare endemic microphyte described by Brullo et al. [135]. It grows together with *Anagallis parviflora*, *Radiola linoides*, *Cicendia filiformis*, *Centaurium maritimum*, *Briza minor*, *Mentha pulegium*, *Juncus bufonius*, *Lythrum hyssopifolia*, etc. It is very peculiar vegetation floristically and is ecologically well differentiated from the other Sicilian association of the *Cicendio-Solenopsion laurentiae*.

Geographical distribution: This association is exclusive of the Isola Grande dello Stagnone near Marsala, where it is very rare (2A).

**20.**  *Solenopsio gasparrinii-Isoëtetum siculae* Brullo, Cambria, Ilardi & Minissale ass. nova, hoc loco (Appendix B, Table A21).

Holotypus: rel. 2, hoc loco

Characteristic species: *Isoëtes sicula*

Structure and ecology: This association is localized in the wide wetlands that are temporarily flooded, especially in the autumn-winter time, and which tend to dry up since the early spring. It grows on silty-sandy soils which keep the humidity throughout the spring and is differentiated by several small hygrophytes showing a high value of coverage. Floristically, this vegetation is characterized by *Isoëtes sicula*, which grows with *Solenopsis gasparrinii*, *Ophioglossum lusitanicum*, *Anagallis parviflora*, *Anagallis minima*, *Radiola linoides*, *Cicendia filiformis*, *Lotus parviflorus*, *Romulea ramiflora*, *Isolepis cernua*, *Juncus bufonius*, etc. Currently, the *Solenopsio gasparrinii-Isoëtetum siculae*, similarly to most microphytic associations of the *Isoëtetalia*, has a very narrow distribution due to human pressure, in particular for the reclamation of wetlands and their use in farming land.

Geographical distribution: This association occurs in western Sicily near Calatafimi and Mazara del Vallo (Trapani) (Figure 5A).

**21.**  *Myosotido congestae-Isoëtetum histricis* Azzaro & Cambria ass. nova, hoc loco (Appendix B, Table A22).

Holotypus: rel. 2, hoc loco.

Characteristic species: *Isoëtes histrix*, *Myosotis congesta*, *Aphanes arvensis*.

Structure and ecology: The wide wetlands characterized by sandy soils, which are flooded by rainwaters in the autumn-winter period, are colonized by peculiar hygrophilous vegetation dominated by *Isoëtes histrix*. In this stand, remaining quite wet during the springtime, some rare microphytes, such as *Myosotis congesta* and *Aphanes arvensis*, occur too. In particular, *Myosotis congesta* was recently quoted as a new record from Italy by Azzaro et al. [136] and subsequently typified by Tavilla et al. [137], which provides some information on its morphological, ecological and chorological features. This vegetation, for its floristic and ecological peculiarities, is proposed as a new association, named *Myosotido congestae-Isoëtetum histricis*, which shows a quite poor floristic set and can be referred to as *Cicendio-Solenopsion laurentiae* for its ecological requirements and the occurrence of *Anagallis parviflora*. The association is localized within the clearings of thermophilous *Quercus suber* woodlands.

Geographical distribution: This association is very rare and was observed only inside the nature reserve “Bosco di Santo Pietro” near Caltagirone (southern Sicily).

*AGROSTION SALMANTICAE* Rivas Goday 1958 Anal. Inst. Bot. Cavanil1es 15: 612

Syn.: *Pre-Isoetion* Rivas Goday 1955, Anal. Inst. Bot. Cavanil1es 8: 385 nom. inval. (art. 3b); *Agrostion salmanticae* Rivas Goday 1956, Anal. Insl. Bol. Cavanilles 8: 387, nom. nud. (art. 2b); *Agrostion pourretii* Rivas Goday 1958, nom. mutand. propos. by Rivas Martinez et al. (2002: 248).

Lectotypus: As. *Pulicaria uliginosa et Agrostis salmantica* Rivas Goday 1956, Anal. Inst. Bot. Cavanilles 8: 386.

Characteristic species: *Agrostis pourretii*, *Chamaemelum fuscatum*, *Trifolium michelianum*.

Structure and ecology: Spring blooming communities, which are linked to humid depressions with long-lasting waters in winter and spring and with predominantly arenaceous soils. Physiognomically, they are dominated by graminoid therophytes and show their optimum in the late spring.

Geographical distribution: The associations of this syntaxon are frequent in the Iberian-Atlantic and West Mediterranean area (Spain, France, Corse, Sardinia, Sicily, and southern Italy). In Sicily, it is localized in the North-West part of the Island (Figure 11).

**22.**  *Trifolio micheliani-Agrostidetum pourretii* Cambria & Brullo ass. nova hoc loco (Appendix B, Table A23).

Syn.: *Trifolio micheliani-Glycerietum spicati* Caldarella, La Rosa, Cusimano, Romano & Gianguzzi 2013, Plant Biosyst. 147(4): 7, *p.p*.

Holotypus: rel. 3, hoc loco.

Characteristic species: *Trifolium michelianum*.

Structure and ecology: In the wide wetlands characterized by silty-sandy soils deposited on flyschoid clays, dense vegetation dominated by *Agrostis pourretii* and *Trifolium michelianum* occurs. In particular, the plant community characterized by these species colonizes small stands localized within clearings of deciduous oak woods, where it is mixed with perennial hygrophilous vegetation linked to surfaces submerged by deeper waters. Previously, Caldarella et al. [138] described a very heterogeneous association including both the perennial vegetation of *Phragmito-Magnocaricetea* and the annual phytocoenosis with *Agrostis pourretii* belonging to *Isoëto-Nanojuncetea*, which was proposed as *Trifolio micheliani-Glycerietum spicati.* Indeed, this association represents very complex vegetation including two well distinct communities belonging to different phytosociological classes. In fact, these authors carried out their relevés on very large surfaces (70–100 m^2^) without respecting the criterion of the floristic-ecological homogeneity of the surveyed vegetation. This is evident in the floristic set of the relevés, including species of various phytosociological typologies (*Phragmito-Magnocaricetea*, *Molinio-Arrhenatheretea* and *Isoëto-Nanojuncetea*). In particular, within these wide wetlands, the plant communities belonging to *Isoëto-Nanojuncetea* are localized on small surfaces (at most 10–20 m^2^), with soils that are already emerged in early spring compared to the surrounding surfaces still inundated. In fact, the floristic set in these stands consists almost exclusively of hygrophilous therophytes belonging to the *Isoëto-Nanojuncetea* class. This vegetation is, therefore, proposed as a new association named *Trifolio micheliani-Agrostidetum pourretii*, which for its floristic and ecological peculiarities must be referred to as *Agrostion salmanticae.* Previously, associations referring to this alliance were described in the Italian territory from North Sardinia by Biondi & Bagella [75] and Bagella et al. [77] and Apulia by Tomaselli et al. [51], represented, respectively, by *Anthoxantho aristati-Agrostidetum salmanticae* and *Phalarido minoris-Agrostidetum pourretii.*

Geographical distribution: This vegetation was surveyed near Ficuzza (North Sicily) (3B).

*NANOCYPERETALIA* Klika 1935, Beih. Bot. Cent.bl. 53: 292, nom. cons. propos. [116]

Syn.: *Nanocypero-Polygonetalia* W. Kock 1926, Jb. St. Gall. Natunviss. Ges. 61:20, nom. rejic. propos.; *Cyperetalia fusci* Müller-Stoll & Pietsch in Lohmeyer et al. 1962, Melhoramento, 15:20; *Cyperetalia fusci* Pietsch 1963, Abh. u. Ber. Naturkundemus 38:3, nom. illeg. (art. 29); *Cicendietalia filiformis* Géhu 1992, Ann. Bot. (Roma) 50: 139, nom. nud. (2b); *Elatino triandrae-Cyperetalia fusci* de Foucault 1988, Dissert. Bot. 121: 78.

Holotypus: *Nanocyperion flavescentis* W. Koch 1926.

Characteristic species: *Centaurium pulchellum*, *Corrigiola litoralis*, *Cyperus fuscus*, *C. michelianus*, *Hordeum marinum*, *Laphangium luteoalbum*, *Lythrum portula*, *Plantago intermedia*, *Spergularia rubra*.

Structure and ecology: This syntaxon group’s ephemeral vegetation localized in wide wet hollows usually flooded until early summer, with soils being mostly eutrophic or sub-eutrophic, often hypertrophic, and usually well nitrified since they are used as pastures, or more rarely oligo-mesotrophic. Floristically, it is differentiated by the occurrence of species with summer-autumn blooming, showing a prostrate and creeping habit.

Geographical distribution: This order is distributed in the Atlantic and central European territories, extending also to the Mediterranean ones with Temperate bioclimate, such as mountain stands or in artificial basins and along the coastal places with slightly brackish soils. 

Notes: According to Fernández-González et al. [116], the correct name of the order at issue is *Nanocypero-Polygonetalia* Koch 1926 for reasons of priority, as it was validly published (Art. 2b, ICPN). Indeed, Koch [3] within this order included two new alliances, such as *Nanocyperion flavescentis* and *Polygono-Chenopodion polyspermi*, of which the second is invalid according to Art. 3f (ICPN), while the *Nanocyperion flavescentis* is a valid alliance. Indeed, Koch [3] did not provide relevés but unambiguously referred it to an association validly published by Braun-Blanquet [139], namely *Junco compressus-Parvo-Cyperus-*Association. Besides, *Cyperus flavescens* L. occurs in the typical relevé, which, therefore, validates the *Cyperetum flavescentis* proposed by Koch [3]. Unfortunately, this name is a superfluous name for the *Junco compressi-Parvo-Cyperetum* (Art. 29c, ICPN). In conclusion, Fernández-González et al. [116], proposed that, as *Nanocypero-Polygonetalia* is a disused name, *Nanocyperetalia* Klika 1935 should be the nomen conservandum, since the latter has always been the name used in literature.

*NANOCYPERION FLAVESCENTIS* Koch 1926, Jb. St. Gall. Natunviss. Ges. 61: 20–28

Syn.: *Nanocyperion flavescentis* W. Koch *ex* Libbert 1932, Verh. Bol. Ver. Prov. Brandenburg 74: 21; *Junclon bufonii* Phi1ippi 1968, Vertiff. Land. Natur. Landsch. Bad.-Würt. 36: 69; *Centaurio-Blackstonion perfoliatae* de Foucault 1988, Dissert. Bot. 121: 84; *Peplidion portulae* Piertch & Müller-Stoll 1974, Verh. Bot. Vereins Prov. Brandenburg 109: 59.

Holotypus: *Junco compressi-Parvo-Cyperetum* Br. Bl. 1922.

Characteristic species: *Blackstonia perfoliata*, *Cyperus flavescens*.

Structure and ecology: This alliance groups plant communities rich in annual small caespitose sedges linked to wet, sandy-clayey, or organic soils, which are submerged for long period by oligo-mesotrophic, freshwater. This vegetation, showing its maximum vegetative development in the summer-autumn period, in Sicily is very rare and localized in the sub-mountain belt.

**Figure 12:**
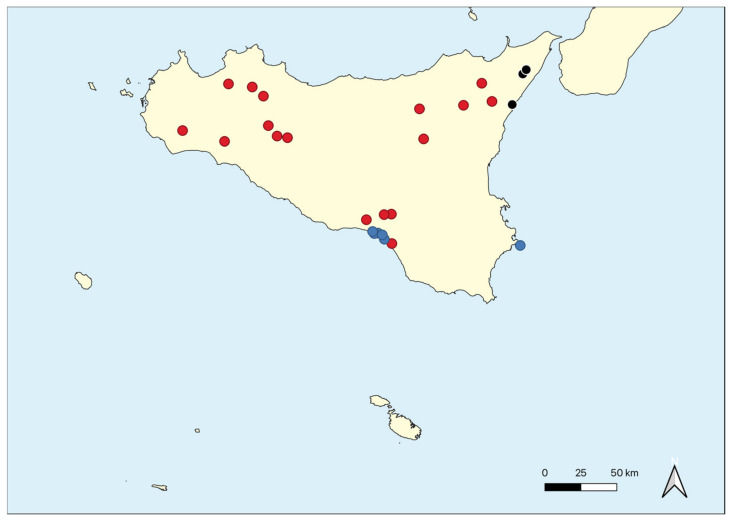
Geographical distribution of the *Nanocyperion flavescentis* (black dot), *Verbenion supinae* (red dot) and *Lythrion tribracteati* (blue dot) associations in Sicily.

Geographical distribution: The associations of this alliance are widespread in the Euro-Siberian region and in particular in the Atlantic and central European territories, while they are less frequent in the Mediterranean ones. As concerns Sicily, it is localized in the North-East part of the Island (Figure 12).

**23.**  *Plantago intermediae-Cyperetum fusci* Sciandrello, D’Agostino & Minissale 2013 (Appendix B, Table A24).

Holotypus: rel. 1, Tab. 7, Sciandrello et al. [140]

Characteristic species: *Plantago intermedia*, *Cyperus fuscus*.

Structure and ecology: The association was surveyed in the small ponds localized on metamorphic substrata with acid soils submerged by freshwater for long period, remaining moist also in the summertime. It seems distributed at an altitude of 600-700 m a.s.l., within the meso-Mediterranean sub-humid belt. Physiognomically, this vegetation is dominated by two caespitose sedges, such as *Cyperus fuscus* and *C. flavescens*, usually growing with *Plantago intermedia*, *Mentha pulegium*, *Juncus hybridus*, *Lythrum hyssopifolia*, *Juncus bufonius*, etc. It can be found in sub-mountains stands characterized by climatophilous deciduous oak woodlands linked to siliceous substrata.

Geographical distribution: This association is circumscribed to a few localities of the Peloritani chain (NE Sicily) (4F).

*VERBENION SUPINAE* Slavnic 1951, Arch. Sci. Mat. Srpska Sci. Nat.1: 146.

Syn.: *Heleochloion* Br.-Bl. 1952, Group. Vég. Fr. Médit.: 72; *Fimbristylion dichotomae* Horvatić 1954, Vegetatio 5: 448; *Dichostylidion micheliani* Horvatić 1963, Acta Biol. 4: 37; *Heleochloo-Cyperion micheliani* Pietsch et Müller-Stoll 1968, 1968, Mitt. Flor.-Soz. Arbeitsgem. nf. 13: 28.

Lectotypus: *Heliotropio-Verbenetum supinae* Slavnić 1951.

Characteristic species: *Coronopus squamatus*, *Sporobolus aculeatus*, *S. alopecuroides*, *S. schoenoides*, *Euphorbia chamaesyce*, *Glinus lotoides*, *Gnaphalium uliginosum* var. *prostratum*, *Heliotropium supinum*, *Hordeum geniculatum*, *Paspalum distichum*, *Pulicaria sicula*, *P. vulgare* var. *graeca*, *Schenkia spicata*, *Teucrium campanulatum*, *Verbena supina*.

Structure and ecology: Ephemeral vegetation occurring in wide depressions, represented by lagoons, lakes, artificial basins, riverbanks, etc., which are subjected to long periods of submersion, usually until early summer, and often characterized by well nitrified soils. In these habitats, flooded by eutrophic or hypertrophic water, prostrate-creeping species, often of large size, having a summer-autumnal blooming are frequent.

Geographical distribution: The communities of this alliance are spread in western and central-eastern Europe and also in the Mediterranean area. As concerns Sicily, it is localized in several parts of the Island (Figure 12).

**24.**  *Gnaphalio luteoalbi-Verbenetum supinae* Rivas Goday 1970, Anales Inst. Bot. Cavanilles 27:273, nom. invers. propos. (Appendix B, Table A25).

Syn.: *Gnaphalium luteo-album-Verbena supina* Rivas Goday 1956, comunidad prov., Anales Inst. Bot. Cavanilles 13:370; *Verbeno-Gnaphalietum luteo-albi* Rivas Goday 1970, Anales Inst. Bot. Cavanilles 27:273.

Lectotypus: rel. 1, Tab. 8, Rivas Goday [22], lectotypus designated by Silva et al. [141].

Characteristic species: *Laphangium luteoalbum*

Structure and ecology: Along the banks of artificial basins, with soils rich in sandy components, maintaining a certain superficial edaphic humidity, even in the summertime, hygrophilous vegetation with sub-nitrophilous requirements occurs. It is dominated by therophytes with prostrate-ascending habits, among them *Verbena supina*, *Paspalum distichum*, *Schenkia spicata*, *Juncus hybridus*, *Euphorbia chamaesyce*, etc. Due to the occurrence and often dominance of *Laphangium luteoalbum*, this community was attributed by Sciandrello [91] to *Verbeno supinae-Gnaphalietum luteo-albi*, association described by Rivas Goday [22] from Iberian Peninsula, previously treated by the same author [19] as *Gnaphalium luteo-album-Verbena supina* comunidad prov. Recently, Silva & Molina [142] renamed this association as *Gnaphalio luteoalbi-Verbenetum supinae* nom. invers. prop. (Arts. 10b and 42, Theurillat et al. [117]).

Geographical distribution: Cimia lake, artificial basin near Mazzarino (Caltanissetta).

**25.**  *Heliotropio supini-Heleochloetum schoenoidis* Rivas Goday 1956, Anales Inst. Bot. Cavanilles 13:371. (Appendix B, Table A26).

Syn.: *Glino-Heliotropietum supini* Brullo & Marcenò 1974 *heliotropietosum* Brullo & Marcenò 1974, Lav. Ist. Bot. Giard, Col. Palermo 25: 189; *Heliotropio supini-Crypsietum schoenoidis* Rivas Goday in Rivas Goday et al. 1956 *nom. mut. nov.*, proposed by Silva et al. (2021), Mediterr. Bot. 42:7.

Lectotypus: rel. 6, Tab. 14 Rivas Goday et al. [19], designated by Silva et al. [141]

Characteristic species: *Heliotropium supinum*, *Sporobolus schoenoides*.

Structure and ecology: On the rather inclined surfaces of artificial basins, covered only superficially by mud, since the submersion period does not last for a long time, sub-nitrophilous vegetation with more xerophilous requirements is localized. Floristically, it is dominated by *Sporobolus schoenoides* (= *Helochloa schoenoides*) and *Heliotropium supinum*, large therophytes with a creeping habit. It was described by Brullo & Marcenò [79] as *Glino-Heliotropietum supini* subass. *heliotropietosum*, which in late summer and early autumn tends to cover large surfaces. As highlighted by Brullo & Minissale [39] and later also by Sciandrello [91], this vegetation must be attributed to *Heliotropio-Heleochloetum schoenoidis* Rivas Goday 1956, the association described from the Iberian Peninsula [22]. More recently, Rivas-Martínez et al. [109] and Silva et al. [141] have proposed to change the name of this association to *Heliotropio supini-Crypsietum schoenoidis* according to Art. 45.

Geographical distribution: Based on literature data of Brullo & Marcenò [79] and Sciandrello [91], this association occurs in some artificial basin of northern, central, and southern Sicily (Scanzano, Prizzi, Pian del Leone, Fanaco, Trinità, Ancipa, Pozzillo, Poma, Comunelli, Disueri) (Figure 4H).

**26.**  *Glino lotoidis-Verbenetum supinae* Rivas Goday 1964, Veg. fl. Cuenca extr. Guadiana: 187 (Appendix B, Table A27).

Syn.: *Glino-Heliotropietum supini* Brullo & Marcenò 1974 *glinetosum* Brullo & Marcenò 1974, Lav. Ist. Bot. Giard, Col. Palermo 25: 190.

Lectotypus: rel. 1, Tab. pg. 188 Rivas Goday [21], designated by Silva et al. [141].

Characteristic species: *Glinus lotoides.*

Structure and ecology: On the more or less flat surfaces characterized by silty-clayey soils, along the shores of artificial basins, drying out during the summer period, sub-nitrophilous hygrophilous vegetation is frequent, which is dominated by therophytes with creeping or prostrate-ascending habit, among them *Sporobolus schoenoides*, *Heliotropium supinum*, *Paspalum disticum*, *Verbena supina*, *Euphorbia chamaesyce*, etc. It is a very peculiar community having its vegetative optimum in the summer-autumn period when the surfaces emerge but with still more or less humid soils. The occurrence of species belonging to *Isoeto-Nanojuncetea* and *Nanocyperetalia* is remarkable since it highlights the hygrophilous requirements of these communities. In particular, the occurrence of *Glinus lotoides* and *Verbena supina* allows referring this vegetation to *Glino lotoidis-Verbenetum supinae*, an association described by Rivas Goday [21] from Spain, recorded by Brullo & Minissale [39] and Sciandrello [91] also from Sicily. Previously, Brullo & Marcenò [79] had attributed this phytocoenosis to *Glino-Heliotropietum supini* subass. *glinetosum*, but for reasons of priority, it must be referred to *Glino lotoidis-Verbenetum supinae* [21,22,109].

Geographical distribution: According to Brullo & Marcenò [79] and Sciandrello [91], this Iberian association is also recorded from the Sicilian artificial basins of Piana degli Albanesi, Scanzano, Arancio, Disueri and Comunelli (Figure 3F).

**27.**  *Coronopo squamati-Sisymbrelletum dentatae* Minissale & Spampinato 1987 (Appendix B, Table A28).

Holotypus: rel. 6, Tab. 8, Minissale & Spampinato [87].

Characteristic species: *Sisymbrella dentata*, *Anthemis cotula*

Structure and ecology: The association was described by Minissale & Spampinato [87] in a depressed area characterized by basaltic substrata, subject to seasonal flooding, often prolonged until the beginning of the summertime. The rocky surfaces are covered with silty-clayey deposits, flooded by shallow waters, and are more or less dried up during the summer. Physiognomically, this vegetation is well differentiated for the dominance of *Sisymbrella dentata*, a rare Sicilian endemism, which generally grows together with other hygrophytes, including *Anthemis cotula*, *Hordeum hystrix*, *Coronopus squamatus*, *Mentha pulegium*, *Teucrium campanulatum*, *Ranunculus sardous*, *R. trilobus*, *Eryngium pusillum*, etc. Currently, the association is rather degraded, since the areas originally occupied by it are subject to cultural activities, due to their transformation into vineyards.

Geographical distribution: The vegetation is mainly diffused in correspondence with the Gurrida Lake, at the base of the Etna Mount near Randazzo. This association was observed also near Castiglione di Sicilia. Probably, this association in the past was much more frequent in Sicily, since *Sisymbrella dentata*, a characteristic species, was recorded in many places on the island, where it has today, unfortunately, disappeared [143] (Figure 3C).

**28.**  *Heleochloo schoenoidis-Chenopodietum botryoidis* Brullo & Sciandrello 2006, Fitosociologia 43(2): 25 (Appendix B, Table A29).

Holotypus: rel. 9, Tab. 2, Brullo & Sciandrello [89].

Characteristic species: *Sporobolus schoenoides*, *Oxybasis chenopodioides (=Chenopodium botryoides)*

Structure and ecology: This association is localized in natural coastal lakes, in correspondence with the peripheral surfaces subject to summer-autumn drying. These stands are characterized by more or less flat and clayey-loamy soils, with a certain concentration of nitrates, still humid under the superficial crust. From the floristic point of view, it appears as an annual pioneer vegetation with a hygro-subnitrophilous character, dominated by therophytes with creeping habits, such as *Sporobolus schoenoides*, *Sporobolus aculeatus*, *Cyperus fuscus*. The differential of the association is *Oxybasis chenopodioides*, a very rare species in Sicily, linked to brackish lake environments, often near the sea, which tends to cover large surfaces along the dried up edges of the basin. As already highlighted by Brullo & Sciandrello [89], due to its floristic and ecological peculiarities, this association shows a remarkable affinity with *Amarantho albi-Chenopodietum botryoidis*, described for the territory of Granada (southern Spain) by Martinez Parras et al. [144].

Geographical distribution: The association was surveyed at Biviere of Gela, a wide coastal lake in southern Sicily.

**29.**  *Coronopo squamati-Corrigioletum litoralis* Brullo & C. Brullo ass. nova, hoc loco (Appendix B, Table A30).

Holotypus: rel. 2, hoc loco. 

Characteristic species: *Corrigiola litoralis.*

Structure and ecology: On the clayey-sandy soils of small ponds, drying out during the summertime, hygrophilous-sub-nitrophilous vegetation, dominated by some therophytes with prostrate-ascending habit, grows. Rather relevant is the occurrence of *Corrigiola litoralis*, which highlights the hygrophilous character of this vegetation, usually growing with *Mentha pulegium*, *Coronopus squamatus*, *Laphangium luteoalbum*, *Lythrum hissopifolia*, *Polypogon subspathaceus*, *Juncus bufonius*, etc. This vegetation is localized in sub-mountain stands on siliceous substrata at about 1200 m a.s.l., within the sub-humid mesomediterranean bioclimatic belt. Due to its floristic and ecological peculiarities, it is proposed as a new association, named *Coronopo squamati-Corrigioletum litoralis*.

Geographical distribution: This association was surveyed near Argimusco in the Peloritani chain.

*LYTHRION TRIBRACTEATI* Rivas Goday & Rivas-Martinez ex Rivas Goday 1970, Anales Inst. Bot. Cavanilles 27: 256.

Syn.: *Lythrion tribracteati* Rivas Goday & Rivas-Mart. 1963, Est. Clas. Past. Espan.: 60 (3b).

Holotypus: *Isolepido-Lythretum castellani* Rivas Goday 1970

Characteristic species: *Lythrum tribracteatum.*

Structure and ecology: This alliance groups the ephemeral, hygrophilous plant communities occurring in wetlands with a long flooding period, having a summer-autumnal optimum. They are linked to silty-clay soils submerged by eutrophic waters, sometimes weakly brackish.

Geographical distribution: This alliance is distributed in the West Mediterranean territory and in Sicily, it occurs in the southern part of the Island (Figure 12).

**30.**  *Damasonio bourgaei-Crypsietum aculeatae* Rivas-Martínez & Costa in Rivas-Martínez et al. 1980 corr. V. Silva & J.C. Costa in Costa et al. 2012, Glob. Geobot. 2: 7 (Appendix B, Table A31).

Syn.: *Damasonio alismae-Crypsietum aculeatae* Rivas-Martínez & Costa in Rivas-Martínez et al. 1980, Lazaroa 2: 31; *Cresso creticae-Damasonietum bourgaei* Sciandrello 2007, Infor. Bot. Ital., 39 (1): 132.

Holotypus: rel. 3, Tab. 15, Rivas-Martínez et al. [145]

Characteristic species: *Cressa cretica*, *Damasonium bourgaei*.

Structure and ecology: This association is localized on flat surfaces with clayey-sandy soils, subject to long periods of submersion. It has its maximum expression in the summer-autumn period, when the soil, which is still quite humid, is no longer submerged. It is a hygrophilous vegetation dominated by therophytes with a prostrate or prostrate-ascending habit, including, in particular, *Damasonium bourgaei*, *Sporobolus aculeatus*, *Heliotropium supinum*, *Coronopus squamatus*, *Euphorbia chamaesyce*, *Paspalum disticum*, etc. The occurrence of *Cressa cretica* is here even more significant since it highlights the halo-nitrophilous requirements of this association. This association was first described by Rivas-Martínez et al. [145] as *Damasonio alismae-Crypsietum aculeatae*, whose name was later corrected by Costa et al. [111] since *Damasonium bourgaei* was misidentified as *D. alisma*. Among the synonyms of this association the *Cresso creticae-Damasonietum bourgaei*, described by Sciandrello [90] from southern Sicily, must be mentioned too. Previously this association was included in *Helochloion* or in the *Verbenion supinae*.

Geographical distribution: From literature data [90] and unpublished relevés, this association in Sicily is localized in the Gela territory. It is also recorded in the Iberian Peninsula [111,145] and Tunisia [146] (Figure 3D, Figure 4E and Figure 5M).

**31.**  *Ranunculo trilobi-Lythretum tribracteati* Brullo & Sciandrello ass. nova hoc loco (Appendix B, Table A32).

Holotypus: rel. 3, hoc loco. 

Characteristic species: *Ranunculus trilobus*

Structure and ecology: The association was surveyed on small marshy surfaces within communities dominated by helophytes belonging to the *Phragmito-Magnocaricetea*. It prefers sandy-loamy soils submerged by slightly salty waters during the winter-spring period while remaining humid during the summer. Floristically this vegetation is characterized by therophytes with erect or prostrate habits, among these can be quoted *Ranunculus trilobus*, usually associated with *Lythrum tribracteatum*, which highlights the sub-halophilous character of this vegetation. Other hydrophytes of *Isoëto-Nanojuncetea* are also frequent, such as *Lythrum hyssopifolia*, *Coronopus squamatus*, *Damasonium bourgaei*, *Pulicaria sicula*, *Hordeum marinum*, *Schenkia spicata*, *Mentha pulegium*, *Polypogon subspathaceus*, etc. It is proposed as a new association, named *Ranunculo trilobi-Lythretum tribracteati.*

Geographical distribution: This association is quite rare and occurs only in some salt marshes near Gela (southern Sicily) (Figure 5L).

**32.**  *Pulicario graecae-Damasonietum bourgaei* Minissale, Santo & Sciandrello 2011, Fitosociologia 48 (2): 81 (Appendix B, Table A33).

Holotypus: rel. 2, Tab. 3, Minissale et al. [92].

Characteristic species: *Pulicaria vugaris* var. *graeca*

Structure and ecology: The association was described by Minissale et al. [92], from a locality near the rocky coast, in correspondence with a small temporary pond, drying up during the summer period. It is limited to a depression within a calcareous outcrop covered by a layer of sandy-clayey soil. Floristically, it is characterized by hygrophilous therophytes with a predominant creeping habit, dominated by *Pulicaria vulgaris* var. *graeca* and *Damasonium bourgaei*. Other species linked to wet habitats, even if rather sporadically, occur, such as *Heliotropium supinum*, *Polypogon subspathaceus*, *Lythrum tribracteatum*, *Coronopus squamatus*, etc. It shows some floristic relationships with *Damasonio bourgaei-Crypsietum aculeatae*, from which it differs substantially in its ecological requirements since the latter association is localized on wide clayey surfaces and shows a more marked halophilous character.

Geographical distribution: This association was surveyed on a stand of the calcareous coast of the Maddalena peninsula near Syracuse. Plant communities referable to this association, but floristically impoverished, were observed in other coastal places from Sicily.

## 3. Materials and Methods

### 3.1. Dataset

The study was carried out over a period of about 50 years with phytosociological investigations in the field and also using the numerous literature data which have been published in the meantime. The present study is based on 394 phytosociological relevés made following the method of Braun Blanquet [147], of which 215 are from literature data and 179 unpublished data (Appendix B). The relevés were carried out in several Sicilian natural areas: the Simeto river (Catania), Capo Murro di Porco and Archeological ruins of Syracuse, Piana del Signore marshes (Gela), Hyblean Plateau, Monte Lauro, the surroundings of Trapani and Palermo, Madonie, Gurrida lake, Nebrodi, Peloritani, etc. The nomenclature of the surveyed syntaxa follows the 4th edition of the International Code of Phytosociological Nomenclature (ICPN, [117]), while the syntaxonomical arrangement follows Brullo and Minissale [39], Rivas Martinez et al. [109], Brullo et al. [148], Biondi et al. [113], and Mucina et al. [114].

### 3.2. Floristic Nomenclautre

As concerns the floristic nomenclature and life-forms, we have followed Pignatti [149,150,151,152] and the Portal to the Flora of Italy [153]. The checklist of the species occurring in the phytosociological relevés is reported in Appendix A. Chorological types follow Brullo et al. [154].

### 3.3. Data Analysis

In order to verify the syntaxonomical relations among the surveyed plant communities, some relevés (up to five based on availability) were selected and the cover-abundance values (following the scale of Braun-Blanquet [147] were transformed according to the method proposed by van Der Maarel [155]. Thus, a matrix of 143 relevés × 158 species was selected from the original data-set and subjected to multivariate analysis, after removing species with a frequency lower than 1%. Hierarchical clustering on the final matrix was performed by using flexible beta linkage, with the Bray–Curtis coefficient. Beta was set at −0.25 so that flexible beta clustering became a space-conserving method [156]. To determine the optimal number of clusters, we used the “Optimclass 1” method (*p* < 10-6) [157], applying the function “Crispness of Classification” to each data set partition. For the ordination analyses, we carried out a Detrended Correspondence Analysis (DCA) according to the Hill & Gauch [158] approach. Hierarchical clustering and ordination analysis were run by PCOrd version 6.08. Optimclass and Crispness of Classification were performed by the software JUICE [159].

## 4. Conclusions

Our survey allowed us to highlight the great diversity of the plant communities belonging to the *Isoëto Nanojuncetea* class occurring in Sicily in many different environments, such as small rocky pools, large ponds, or the banks of artificial basins. In Sicily, they are mainly found on carbonatic, volcanic, siliceous, or clayey substrata. Although we do not have a detailed mapping, on the basis of our expert-based we can affirm that most of the detected plant communities fall into protected areas, such as regional parks, nature reserves and Natura 2000 sites, but this is not enough to guarantee their real protection, because their existence depends above all on good land management and often on a delicate balance between grazing and agro-silvopastoral activities [160]. All the plant communities of *Isoëto-Nanojuncetea* treated here, due to their attribution to Mediterranean temporary ponds, can be referred to as the habitats of priority interest codified as 3170* according to the Annex II of the Habitat Directive (92/43/EEC), whereby they require rigorous protection by the States of the European Union [1]. The criticality of this type of habitat, being temporary ponds, is above all linked to the fact that in many cases they are limited to small surfaces, apart from having a very fragmented distribution. All this tends to make them not very visible and does not give them sufficient importance, therefore, they are usually neglected. Overall, they are quite vulnerable, even to involuntary destruction [161,162], or to changes in land use, which together can contribute to the disappearance or alteration of these relevant micro-habitats [163]. Another problem with the protection and management of these habitats is that in many cases, covering very small surfaces, they escape the cartographic surveys of vegetation on a regional scale, which happened in Sicily [164,165]. Even in the inventories of wetlands on a regional or national scale, Mediterranean temporary ponds are largely under-represented, thus limiting the possibilities of protection and correct management [166]. It is, therefore, understood that there is a need to intensify regional field surveys in order to have a better awareness of the real distribution of this habitat. It is to be hoped that in the future these results will stimulate adequate research and management policies on these Mediterranean temporary ponds and their conservation. Unfortunately, this cannot be separated from effective and coordinated governance at the national level, based on multiple spatial scales ranging from land-use policies to the management of protected areas, agricultural areas, and so on [167].

## Data Availability

Not applicable.

## Appendix A

**Table A1:** Checklist of the taxa occurring in the phytosociological relevés with their life forms and chorotypes.

Taxon	Life Form	Chorotype
*Agrostis pourretii* Willd.	T scap	W Medit.
*Agrostis stolonifera* L.	H rept	Circumbor.
*Aira cupaniana* Guss.	T scap	W Medit.
*Ajuga reptans* L.	H rept	Euro.-Asiat.
*Alisma lanceolatum* With.	I rad	Subcosmop.
*Allium obtusiflorum* Redouté	G bulb	End. Sic.
*Allium subhirsutum* L.	G bulb	Medit.
*Amaranthus deflexus* L.	T scap	Avv.
*Anagallis arvensis* L.	T scap	Paleotemp.
*Anagallis minima* (L.) E. H. L. Krause	T scap	Paleotemp.
*Anagallis parviflora* Hoffmanns. & Link.	T scap	W Medit.
*Anthemis arvensis* L.	T scap	Euro-Medit.
*Anthemis cotula* L.	T scap	Euro-Medit.
*Anthoxantum odoratum* L.	T scap	Euro.-Asiat.
*Antinoria insularis* Parl.	T scap	W Medit.
*Aphanes arvensis* L.	T scap	Subcosmop.
*Archidium alternifolium* (Hedw.) Mitt.	Bryo.	Europ.
*Asteriscus aquaticus* (L.) Less.	T scap	Euro-Medit.
*Atriplex patula* L.	T scap	Circumbor.
*Atriplex prostrata* Boucher ex DC.	T scap	Circumbor.
*Barbarea bracteosa* Guss.	T scap	N Medit.
*Bellis annua* L.	T scap	Medit.
*Bellis perennis* L.	H ros	Euro.-Asiat.
*Beta maritima* L.	H ros	Euro-Medit.
*Biscutella maritima* Ten.	T scap	End. Ital.
*Blackstonia acuminata* (W.D.J. Koch et Ziz) Domin	T scap	Medit.
*Blackstonia perfoliata* (L.) Huds.	T scap	Euro-Medit.
*Bolboschoenus maritimus* (L.) Palla	G rhiz	Cosmop.
*Briza maxima* L.	T scap	Paleotrop.
*Briza minor* L.	T scap	Subcosmop.
*Bromus hordeaceus* L.	T scap	Subcosmop.
*Bryum alpinum* Huds. ex With.	Bryo.	Europ.
*Bulliarda vaillantii* (Willd.) DC.	T scap	Euro-Medit.
*Callitriche brutia* Petagna	I rad	Medit-Atl.
*Callitriche palustris* L.	I rad	Circumbor.
*Callitriche stagnalis* Scop.	I rad	Euro.-Asiat.
*Callitriche truncata* Guss.	I rad	Medit-Atl.
*Cardamine hirsuta* L.	T scap	Paleotrop.
*Cardamine occulta* Hornem.	T scap	Avv.
*Carduus argyroa* Biv.	T scap	C Medit.
*Carex distachya* Desf.	H caesp	Medit.
*Carex divisa* Huds.	H caesp	Paleotemp.
*Carex leporina* L.	H caesp	Euro.-Asiat.
*Carex serrulata* Biv. ex Spreng.	H caesp	Europ.
*Catabrosa aquatica* (L.) P.Beauv.	T scap	Circumbor.
*Catapodium rigidum* (L.) C.E. Hubb.	T scap	Medit-Atl.
*Centaurium erythraea* Rafn	T scap	Paleotemp.
*Centaurium maritimum* (L.) Fritsch.	T scap	Medit.
*Centaurium tenuiflorum* (Hoffmanns. & Link) Fritsch	T scap	Paleotemp.
*Cerastium glomeratum* Thuill.	T scap	Paleotemp.
*Cerastium semidecandrum* L.	T scap	Euro-Medit.
*Chamaemelum fuscatum* (Brot.) Vasc.	T scap	W Medit.
*Chenopodium album* L.	T scap	Subcosmop.
*Chrozophora tinctoria* (L.) A. Juss.	T scap	Medit-Iran-Turan.
*Cicendia filiformis* (L.) Delarbre	T scap	Medit-Atl.
*Cichorium intybus* L.	H scap	Cosmop.
*Cichorium pumilum* Jacq.	T scap	Medit.
*Cirsium italicum* DC.	T scap	SE Europ.
*Coleostephus myconis* (L.) Cass. ex Rchb. f.	T scap	Medit.
*Convolvulus arvensis* L.	G rhiz	Paleotemp.
*Convolvulus silvaticus* Kit.	H scand	SE Europ.
*Conyza bonariensis* L.	T scap	Avv.
*Coronilla repanda* (Poir.) Guss.	T scap	W Medit.
*Coronopus squamatus* (Forssk.) Asch.	T rept	Euro-Medit.
*Corrigiola litoralis* L.	T rept	Medit-Atl.
*Cressa cretica* L.	T scap	Subcosmop.
*Cynodon dactylon* (L.) Pers.	H rept	Cosmop.
*Cynosurus cristatus* L.	H caesp	Europ.
*Cyperus distachyos* All.	G rhiz	Subcosmop.
*Cyperus flavescens* L.	T caesp	Subcosmop.
*Cyperus fuscus* L.	T caesp	Paleotemp.
*Cyperus michelianus* (L.) Delile	T caesp	Paleotemp.
*Cyperus rotundus* L.	G rhiz	Subcosmop.
*Damasonium bourgaei* Coss.	I rad	Medit-Atl.
*Damasonium polyspermum* Coss.	I rad	W Medit.
*Digitaria ciliaris* (Retz.) Koeler	T scap	Tropical.
*Dittrichia viscosa* (L.) Greuter	H scap	W Medit.
*Dysphania ambrosioides* (L.) Mosyakin & Clemants	T scap	Avv.
*Ecballium elaterium* (L.) A. Rich.	T scap	Medit.
*Echinochloa crus-galli* (L.) P. Beauv.	T scap	Subcosmop.
*Echium pustulatum* Sm.	T scap	Europ.
*Elatine campylosperma* Seub.	I rad	Medit.
*Elatine gussonei* (Sommier) Brullo, Lanfr., Pavone & Ronsisv.	I rad	End. Lampedusa-Malta
*Elatine macropoda* Guss.	I rad	Medit.
*Eleocharis nebrodensis* Parl.	G rhiz	End. Sic.
*Eleocharis palustris* (L.) Roem. et Schult.	G rhiz	Subcosmop.
*Elymus repens* (L.) Gould	G rhiz	Circumbor.
*Epilobium parviflorum* Schreb.	T scap	Paleotemp.
*Eryngium pusillum* L.	H bienn	S Medit.
*Euphorbia akenocarpa* Guss.	T scap	SW Medit.
*Euphorbia chamaesyce* L.	T rept	Euro-Medit.
*Euphorbia exigua* L.	T scap	Euro-Medit.
*Euphorbia pterococca* Brot.	T scap	Medit.
*Festuca geniculata* (L.) Lag. & Rodr.	T scap	Medit-Atl.
*Filago pyramidata* L.	T scap	Euro.-Asiat.
*Frankenia hirsuta L.*	Ch suffr	Medit.
*Galium debile* Desv.	H scap	Medit.
*Galium elongatum* C. Presl	H scap	Euro-Medit.
*Galium murale* (L.) All.	T scap	Medit.
*Gaudinia fragilis* (L.) P. Beauv.	T scap	Euro-Medit.
*Geranium dissectum* L.	T scap	Euro-Medit.
*Geranium robertianum* L.	T scap	Circumbor.
*Glebionis segetum* (L.) Fourr.	T scap	Euro-Medit
*Glinus lotoides* L.	T scap	Paleotrop.
*Glyceria notata* Chevall.	I rad	Subcosmop.
*Glyceria spicata* Guss.	I rad	W Medit.
*Gnaphalium uliginosum* L. var. *prostratum* Huet	T scap	End. Ital.
*Heliotropium curassavicum* L.	Ch suffr	Avv.
*Heliotropium europaeum* L.	T scap	Medit-Iran-Turan.
*Heliotropium supinium* L.	T scap	Paleotrop.
*Helminthotheca echioides* (L.) Holub	T scap	Euro-Medit.
*Helosciadium inundatum* (L.) W.D.J.Koch	I rad	Medit-Atl.
*Helosciadium nodiflorum* (L.) W.D.J.Koch	H scap	Euro-Medit.
*Hordeum geniculatum* All.	T scap	Euro-Medit.
*Hordeum marinum* Huds.	T scap	Medit-Atl.
*Hypericum hircinum* L. subsp. *majus* (Aiton) N. Robson	NP	Medit.
*Hypericum pubescens* Boiss.	H scap	W Medit.
*Hypochaeris radicata* L.	H ros	Euro-Medit.
*Hypochoeris achyrophorus* L.	T scap	Medit.
*Isoëtes durieui* Bory	G bulb	W Medit.
*Isoëtes histrix* Bory	G bulb	Medit-Atl.
*Isoëtes longissima* Bory	G bulb	W Medit.
*Isoëtes sicula* Tod.	G bulb	CE Medit.
*Isoëtes todaroana* Troìa & Raimondo	G bulb	Medit.
*Isolepis cernua* (Vahl) Roem. & Schult.	T scap	Subcosmop.
*Juncus articulatus L.*	G rhiz	Circumbor.
*Juncus bufonius* L.	T scap	Cosmop.
*Juncus capitatus* Weigel	T scap	Euro-Medit.
*Juncus compressus* Jacq.	G rhiz	Euro.-Asiat.
*Juncus foliosus* Desf.	T scap	W Medit.
*Juncus hybridus* Brot.	T scap	Medit-Atl.
*Juncus maritimus* Lam.	G rhiz	Subcosmop.
*Juncus pygmaeus* Rich.	T scap	Medit-Atl.
*Juncus subulatus* Forssk.	G rhiz	S Medit.
*Kickxia cirrhosa* (L.) Fritsch.	T scap	Medit.
*Kickxia spuria* (L.) Dumort. subsp. *integrifolia* (Brot.) R. Fern.	T scap	Euro.-Asiat.
*Laphangium luteo-album* (L.) Tzvelev	T scap	Subcosmop.
*Limonium narbonense* Mill.	H ros	Medit.
*Linum bienne* Mill.	T scap	Medit-Atl.
*Logfia gallica* (L.) Cosson & Germ.	T scap	Medit.
*Lolium perenne* L.	H caesp	Euro-Medit.
*Lotus angustissimus* L.	T scap	Euro-Medit.
*Lotus conimbricensis* Brot.	T scap	Medit.
*Lotus hispidus* DC.	T scap	Medit-Atl.
*Lotus parviflorus* Desf.	T scap	Medit.
*Lotus preslii* Ten.	H scap	Euro-Medit.
*Lotus tetragonolobus* L.	T scap	Medit.
*Lythrum hyssopifolia* L.	T scap	Subcosmop.
*Lythrum junceum* Banks & Sol.	H scap	Medit.
*Lythrum tribracteatum* Spreng.	T scap	Euro-Medit.
*Malva sylvestris* L.	T scap	Euro-Medit.
*Medicago ciliaris* (L.) All.	T scap	S Medit.
*Medicago doliata* Carmign.	T scap	Medit.
*Medicago intertexta* (L.) Mill.	T scap	Medit.
*Medicago polymorpha* L.	T scap	Medit-Iran-Turan.
*Medicago turbinata* (L.) All.	T scap	Euro-Medit.
*Mentha pulegium* L.	H scap	Euro-Medit.
*Mentha suaveolens* Ehrh.	H scap	Euro-Medit.
*Middendorfia borysthenica* (Schrank) Trautv.	T scap	Euro-Medit.
*Moenchia erecta* (L.) G. Gaertn., B. Mey. & Scherb.	T scap	Medit-Atl.
*Molineriella minuta* (L.) Rouy.	T scap	Medit.
*Montia arvensis* Wallr.	I rad	Medit-Atl.
*Moraea sisyrinchium* (L.) Ker Gawl.	G bulb	Medit-Iran-Turan.
*Myosotis congesta* Shuttlew ex Albert & Reyn	T scap	Medit.
*Myosotis ramosissima* Rochel	T scap	Euro.-Asiat.
*Myosotis sicula* Guss.	T scap	N Medit.
*Myosotis tineoi* C. Brullo & Brullo	T scap	End. Sic.
*Myosurus minimus* L.	T scap	Subcosmop.
*Nasturtium officinale* W.T. Aiton	H scap	Cosmop.
*Oenanthe fistulosa* L.	H scap	Euro.-Asiat.
*Oenanthe globulosa* L.	H scap	Medit.
*Oenanthe pimpinelloides* L.	H scap	Medit-Atl.
*Ophioglossum lusitanicum* L.	T scap	Paleotemp.
*Ornithogalum gussonei* Ten.	G bulb	E Medit.
*Oxybasis chenopodioides* (L.) S.Fuentes, Uotila & Borsch	T scap	Subcosmop.
*Oxybasis rubra* (L.) S. Fuentes, Uotila & Borsch	T scap	Circumbor.
*Parapholis cylindrica* (Willd.) Romero Zarco	T scap	Euro-Medit.
*Parapholis incurva* (L.) C.E.Hubb.	T scap	Medit-Atl.
*Parentucellia latifolia* (L.) Caruel	T scap	Euro-Medit.
*Paronychia echinulata* Chater	T scap	Medit.
*Paspalum distichum* L.	G rhiz	Subcosmop.
*Persicaria lapathifolia* (L.) Delarbre	T scap	Circumbor.
*Persicaria maculosa* Gray	T scap	Subcosmop.
*Phalaris minor* Retz.	T scap	Paleotrop.
*Phragmites australis* (Cav.) Trin. ex Steud.	G rhiz	Subcosmop.
*Phyla nodiflora* (L.) Greene	H rept	Tropical.
*Pilularia minuta* Durieu	I rad	Medit.
*Plantago afra* L.	T scap	Medit-Iran-Turan.
*Plantago bellardii* All.	T scap	Medit.
*Plantago coronopus* L.	T scap	Euro-Medit.
*Plantago cupanii* Guss.	H ros	SW Medit.
*Plantago intermedia* Gilib.	H ros	Euro-Medit.
*Plantago lagopus* L.	T scap	Medit-Iran-Turan.
*Plantago lanceolata* L.	T scap	Euro.-Asiat.
*Plantago major* L.	H ros	Paleotemp.
*Pleurochaete squarrosa* (Brid.) Lindb.	Bry.	Medit.-Atl.
*Poa annua* L.	T scap	Cosmop.
*Poa bulbosa* L.	H caesp	Paleotemp.
*Poa infirma* Kunth	T scap	Euro-Medit.
*Podospermum canum* C.A. Mey.	H scap	Euro.-Asiat.
*Polygonum arenastrum* Boreau	T rept	Subcosmop.
*Polygonum aviculare* L.	T rept	Cosmop.
*Polypogon maritimus* Willd.	T scap	Medit-Iran-Turan.
*Polypogon monspeliensis* (L.) Desf.	T scap	Paleotrop.
*Polypogon subspathaceus* Req.	T scap	Medit.
*Polypogon viridis* (Gouan) Breistr.	H caesp	Paleotrop.
*Portulaca oleracea* L.	T scap	Subcosmop.
*Potentilla reptans* L.	H rept	Paleotemp.
*Prunella vulgaris* L.	H scap	Circumbor.
*Pulicaria dysenterica* (L.) Bernh.	H scap	Euro-Medit.
*Pulicaria odora* (L.) Rchb.	H scap	Euro-Medit.
*Pulicaria sicula* (L.) Moris	T scap	Medit.
*Pulicaria vulgaris* Gaertn. var. *graeca* (Sch.-Bip.) Fiori	T scap	S Medit.
*Pulicaria vulgaris* Gaertn. var. *vulgaris*	T scap	Paleotemp.
*Radiola linoides* Roth.	T scap	Paleotemp.
*Ranunculus angulatus* C. Presl	H scap	End. Sic.
*Ranunculus lanuginosus* L.	H scap	Europ.
*Ranunculus lateriflorus* DC.	T scap	Paleotrop.
*Ranunculus marginatus* d’Urv.	T scap	E Medit.
*Ranunculus muricatus* L.	T scap	Euro-Medit.
*Ranunculus ophioglossifolius* Vill.	T scap	Euro-Medit.
*Ranunculus paludosus* Poir.	H scap	Medit-Iran-Turan.
*Ranunculus parviflorus* L.	T scap	Medit-Atl.
*Ranunculus peltatus* Schrank	I rad	Europ.
*Ranunculus pratensis* C. Presl	H scap	End. Sard. Sic.
*Ranunculus saniculifolius* Viv.	I rad	Medit.
*Ranunculus sardous* Crantz	T scap	Euro-Medit.
*Ranunculus trilobus* Desf.	T scap	Medit.
*Romulea ramiflora* Ten.	G bulb	Medit.
*Rostraria hispida* (Savi) Doğan	T scap	Medit.
*Rumex conglomeratus* Murray	H scap	Euro.-Asiat.
*Rumex pulcher* L.	H scap	Euro-Medit.
*Sagina apetala* Ard.	T scap	Euro-Medit.
*Sagina subulata* (Sw.) C. Presl	H caesp	Euro-Medit.
*Samolus valerandi* L.	H scap	Subcosmop.
*Saxifraga tridactylites* L.	T scap	Medit-Atl.
*Schenkia spicata* (L.) G.Mans.	T scap	Euro-Medit.
*Schoenoplectus tabernaemontani* (C.C. Gmel.) Palla	G rhiz	Euro.-Asiat.
*Scirpoides holoschoenus* (L.) Soják	G rhiz	Euro-Medit.
*Scolymus maculatus* L.	T scap	S Medit.
*Scorpiurus muricatus* L.	T scap	Euro-Medit.
*Scorzoneroides muelleri* (Sch. Bip.) Greuter & Talavera	T scap	S Medit.
*Sedum caeruleum* L.	T succ	SW Medit.
*Sedum rubens* L.	T succ	Medit-Atl.
*Senecio vulgaris* L.	T scap	Cosmop.
*Serapias lingua* L.	G bulb	Medit.
*Sherardia arvensis* L.	T scap	Euro-Medit.
*Silene gallica* L.	T scap	Euro-Medit.
*Sisymbrella dentata* (L.) O.E. Schulz.	T scap	End. Sic.
*Solanum nigrum* L.	T scap	Euro.-Asiat.
*Solenopsisgasparrinii* (Tineo) Brullo	T scap	Medit.
*Solenopsis laurentia* (L.) C.Presl subsp. *hyblaea* Brullo et al.	T scap	End. Sic.
*Solenopsis mothiana* C. Brullo, Brullo & Giusso	T scap	End. Sic.
*Sonchus asper* (L.) Hill	T scap	Euro.-Asiat.
*Sonchus oleraceus* L.	T scap	Subcosmop.
*Spergularia bocconei* (Scheele) Graebn.	T scap	Subcosmop.
*Spergularia madoniaca* Lojac.	T scap	End. Sic.
*Spergularia marina* (L.) Besser	T scap	Subcosmop.
*Spergularia rubra* (L.) J. Presl & C. Presl.	T scap	Subcosmop.
*Sporobolus aculeatus* (L.) P.M. Peterson	T scap	Paleotemp.
*Sporobolus alopecuroides* (Piller & Mitterp.) P.M. Peterson	T scap	Medit-Iran-Turan.
*Sporobolus schoenoides* (L.) P.M. Peterson	T scap	Paleotemp.
*Stellaria media* (L.) Vill.	T scap	Cosmop.
*Suaeda spicata* (Willd.) Moq.	T succ	Medit.
*Sulla coronaria* (L.) Medik.	T scap	W Medit.
*Symphyotrichum squamatum* (Spreng.) G.L. Nesom	H scap	Avv.
*Tamarix africana* Poir.	P scap	W Medit.
*Tamarix gallica* L.	P scap	W Medit.
*Teucrium campanulatum* L.	H scap	W Medit.
*Tillaea alata* Viv.	T succ	Paleotemp.
*Tillaea muscosa* L.	T succ	Medit-Atl.
*Trifolium campestre* Schreb.	T scap	Paleotemp.
*Trifolium fragiferum* L.	T scap	Paleotemp.
*Trifolium grandiflorum* Schreb.	T scap	E Medit.
*Trifolium lappaceum* L.	T scap	Euro-Medit.
*Trifolium leucanthum* M. Bieb.	T scap	E Medit.
*Trifolium michelianum* Savi	T scap	W Medit.
*Trifolium micranthum* Viv.	T scap	Paleotemp.
*Trifolium nigrescens* Viv.	T scap	Euro-Medit.
*Trifolium pratense* L.	H rept	Subcosmop.
*Trifolium resupinatum* L.	T scap	Paleotemp.
*Trifolium scabrum* L.	T scap	Euro-Medit.
*Trifolium spumosum* L.	T scap	Medit.
*Trifolium squarrosum* L.	T scap	Medit.
*Trifolium strictum* L.	T scap	Euro-Medit.
*Trifolium subterraneum* L.	T rept	Euro-Medit.
*Trifolium suffocatum* L.	T scap	Medit-Atl.
*Trifolium tomentosum* L.	T scap	Paleotemp.
*Triglochin barrelieri* Loisel.	G bulb	Medit.
*Triglochin laxiflora* Guss.	G bulb	W Medit.
*Trigonella sicula* (Turra) Coulot & Rabaute	T scap	S Medit.
*Trigonella sulcata* (Desf.) Coulot & Rabaute	T scap	S Medit.
*Trisetaria aurea* (Ten.) Pignatti ex Kerguélen	T scap	E Medit.
*Tussilago farfara* L.	G rhiz	Euro.-Asiat.
*Typha latifolia* L.	G rhiz	Cosmop.
*Valantia muralis* L.	T scap	Medit.
*Valerianella eriocarpa* Desv.	T scap	Medit-Atl.
*Verbena officinalis* L.	H scap	Subcosmop.
*Verbena supina* L.	T scap	Euro-Medit.
*Veronica anagallis-aquatica* L.	H scap	Cosmop.
*Veronica arvensis* L.	T scap	Subcosmop.
*Veronica serpyllifolia* L.	H scap	Circumbor.
*Visnaga daucoides* Gaertn.	T scap	Medit-Iran-Turan.
*Warnstorfia fluitans* (Hedw.) Loeske	Bry.	Cosmop.
*Xanthium italicum* Moretti	T scap	S Europ.
*Xanthium spinosum* L.	T scap	Avv.

## Appendix B

**Table A2:** *Isoëtetum durieui* Br.-Bl. 1936.

Relevé number	1	2	3	4	5	6	7	8	9	10	Presences
Altitude (dam)	127	127	80	80	80	80	80	35	35	35	
Surface (m^2^)	1	0.5	3	3	0.4	3	2	5	5	5	
Coverage (%)	80	100	80	80	95	90	90	90	90	80	
**Char. Association and All. (ISOËTION)**											
*Isoëtes durieui*	3	4	4	4	4	2	2	3	1	2	10
*Lotus conimbricensis*	.	.	+	+	1	.	.	.	.	.	3
*Ranunculus trilobus*	.	.	.	.	.	.	.	+	+	+	3
**Char. Ord. (ISOËTETALIA)**											
*Lotus angustissimus*	1	1	.	.	.	.	.	+	.	+	4
*Bulliarda vaillantii*	.	.	.	.	.	.	.	2	1	1	3
*Ranunculus saniculifolius*	.	.	.	.	.	.	.	1	+	+	3
*Ranunculus lateriflorus*	.	.	.	.	.	.	.	2	1	1	3
*Trifolium micranthum*	1	1	.	.	.	.	.	.	.	.	2
*Myosotis sicula*	.	.	.	.	.	.	.	.	.	1	1
*Romulea ramiflora*	.	.	.	.	.	.	.	+	.	.	1
**Char. Class (ISOËTO-NANOJUNCETEA)**											
*Mentha pulegium*	3	2	.	.	3	3	3	2	1	+	8
*Juncus capitatus*	1	.	1	+	1	2	2	.	.	.	6
*Juncus bufonius*	1	+	1	1	.	1	+	.	.	.	6
*Eryngium pusillum*	.	.	.	.	.	.	.	2	2	1	3
*Coronopus squamatus*	.	.	.	.	.	.	.	1	2	+	3
*Callitriche brutia*	.	.	.	.	.	.	.	1	+	+	3
*Polypogon subspathaceus*	.	.	.	.	.	.	.	3	2	3	3
*Pulicaria vulgaris* var. *vulgaris*	.	.	.	.	.	.	.	2	1	+	3
*Lythrum hyssopifolia*	.	.	.	.	.	.	.	.	.	+	1
**Other species**											
*Aira cupaniana*	.	.	1	1	1	+	+	.	.	.	5
*Bellis perennis*	.	2	+	+	.	1	1	.	.	.	5
*Moenchia erecta*	+	.	+	+	+	.	+	.	.	.	5
*Prunella vulgaris*	+	.	+	+	.	.	+	.	.	.	4
*Carex divisa*	.	.	.	.	.	.	+	1	+	2	4
*Hypochaeris radicata*	.	.	1	+	.	.	+	.	.	.	3
*Trifolium fragiferum*	.	.	.	.	.	.	.	+	+	+	3
*Linum bienne*	.	.	+	+	.	.	+	.	.	.	3
*Glyceria notata*	.	.	.	.	.	.	.	2	3	1	3
*Eleocharis palustris*	.	.	.	.	.	.	.	+	1	1	3
*Ranunculus lanuginosus*	.	.	.	.	.	+	+	.	.	.	2
*Bromus hordeaceus*	+	+	.	.	.	.	.	.	.	.	2
*Anagallis arvensis*	+	.	.	+	.	.	.	.	.	.	2
*Trifolium campestre*	+	+	.	.	.	.	.	.	.	.	2
*Cynosurus cristatus*	1	.	.	.	+	.	.	.	.	.	2
*Vulpia geniculata*	+	+	.	.	.	.	.	.	.	.	2
*Trifolium scabrum*	1	+	.	.	.	.	.	.	.	.	2
*Potentilla reptans*	.	+	.	.	.	.	.	.	.	.	1
*Dittrichia viscosa*	.	.	.	+	.	.	.	.	.	.	1
*Trifolium nigrescens*	.	.	+	.	.	.	.	.	.	.	1
*Cerastium glomeratum*	.	+	.	.	.	.	.	.	.	.	1
*Pulicaria odora*	.	.	.	+	.	.	.	.	.	.	1
*Orchis* sp.	.	.	.	+	.	.	.	.	.	.	1
*Ranunculus paludosus*	.	+	.	.	.	.	.	.	.	.	1
*Pulicaria dysenterica*	.	.	.	.	+	.	.	.	.	.	1

**Localities and dates of relevés.** Rel. 1–2: Petralia, Pollicino Margio—20 June 1975; rel. 3–7: Piana degli Albanesi, Gorgo Dingoli, Marcenò & Trapani [85]—Table 1, rel. 1–3, 6–7; rel. 8–10: Cozzofico (Carlentini)—4 May 2009.

**Table A3:** *Pulicario graecae-Isolepidetum cernuae* Brullo & Di Martino 1974.

Relevé number	1	2	3	4	5	6	7	8	9	10	11	Presences
Altitude (m)	-	-	-	-	-	-	-	-	-	-	-	
Surface (m^2^)	3	4	1	8	1	0.5	2	3	3	5	2	
Coverage (%)	40	90	30	30	50	60	60	60	70	80	70	
**Char. Association**												
*Pulicaria vulgaris* var. *graeca*	.	+	1	1	1	+	2	1	+	2	1	10
*Damasonium polyspermum*	.	.	.	.	.	+	2	1	+	1	+	6
**Char. All. (ISOËTION) and Ord. (ISOËTETALIA)**												
*Isolepis cernua*	2	4	1	2	2	3	3	3	3	2	3	11
*Centaurium maritimum*	+	.	+	+	1	+	+	+	+	+	+	10
*Middendorfia borysthenica*	.	.	.	.	.	.	.	.	.	2	2	2
*Damasonium bourgaei*	.	.	.	.	.	.	.	.	.	+	+	2
*Lotus hispidus*	1	.	.	.	.	.	+	.	.	.	.	2
**Char. Class (ISOËTO-NANOJUNCETEA)**												
*Lythrum hyssopifolia*	2	3	2	+	1	2	1	2	3	+	2	11
*Juncus capitatus*	1	+	+	+	2	1	+	1	1	+	+	11
*Polypogon subspathaceus*	2	2	2	+	1	2	2	2	1	1	2	11
*Mentha pulegium*	1	.	+	1	1	+	+	2	.	1	+	9
*Spergularia rubra*	.	.	+	+	1	1	.	.	.	+	1	6
*Juncus bufonius*	.	.	.	+	.	.	+	1	+	.	.	4
*Juncus pygmaeus*	.	.	.	.	.	.	.	+	+	1	+	4
*Gaudinia fragilis*	+	.	.	.	.	+	.	.	.	.	.	2
**Other species**												
*Polypogon maritimus*	+	+	+	2	2	1	+	1	2	2	2	11
*Juncus subulatus*	.	.	.	+	.	.	+	+	.	.	.	3
*Callitriche palustris*	.	.	.	.	.	.	.	.	.	3	2	2
*Parapholis incurva*	.	+	.	.	.	.	.	.	+	.	.	2
*Plantago coronopus*	.	+	.	.	.	.	.	.	1	.	.	2
*Bolboschoenus maritimus*	.	.	.	.	+	+	.	.	.	.	.	2
*Centaurium erythraea*	.	.	.	.	.	.	+	.	.	.	+	2
*Cressa cretica*	.	.	.	+	.	.	+	.	.	.	.	2
*Coleostephus myconis*	+	.	.	.	.	.	.	.	.	.	.	1
*Logfia gallica*	+	.	.	.	.	.	.	.	.	.	.	1
*Anagallis arvensis*	+	.	.	.	.	.	.	.	.	.	.	1
*Frankenia hirsuta*	.	.	.	+	.	.	.	.	.	.	.	1

**Localities and dates of relevés.** Rel. 1–11: Isola Grande dello Stagnone (Marsala), Brullo & Di Martino [78]—Table 17.

**Table A4:** *Isoëto durieui-Ranunculetum parviflori* Brullo, Di Martino & Marcenò 1977.

Relevé number	1	2	3	4	5	6	7	8	9	Presences
Altitude (dam)	61	61	61	61	61	61	61	61	61	
Surface (m^2^)	1	1	2	1	2	2	0.5	0.5	2	
Coverage (%)	100	100	80	100	70	60	90	100	100	
**Char. Association**										
*Ranunculus parviflorus*	4	3	3	2	3	3	2	3	+	9
**Char. Subassociation**										
*Callitriche brutia*	.	.	.	.	.	.	.	.	4	1
**Char. All. (ISOËTION)**										
*Isoëtes durieui*	3	2	3	4	2	2	1	3	+	9
*Ranunculus trilobus*	2	2	1	1	1	+	+	1	1	9
**Char. Ord. (ISOËTETALIA)**										
*Ranunculus muricatus*	1	+	1	2	1	2	3	1	2	9
*Lotus angustissimus*	.	1	+	+	+	1	+	1	+	8
**Char. Class (ISOËTO-NANOJUNCETEA)**										
*Lythrum hyssopifolia*	+	+	.	+	+	+	2	+	1	8
*Juncus bufonius*	+	+	1	+	+	.	+	+	1	8
*Mentha pulegium*	.	.	.	.	+	+	1	+	2	5
**Other species**										
*Myosotis ramosissima*	1	1	+	1	+	1	1	+	.	8
*Geranium robertianum*	+	+	+	+	+	.	+	+	.	7
*Coleostephus myconis*	.	+	.	+	+	+	1	1	.	7
*Cardamine hirsuta*	+	+	.	.	+	+	.	+	+	6
*Galium murale*	.	.	.	.	1	1	2	1	2	5
*Poa annua*	1	1	+	1	.	.	.	.	.	4
*Stellaria media*	+	1	+	+	.	.	.	.	.	4
*Carex distachya*	1	+	+	1	.	.	.	.	.	4
*Sherardia arvensis*	+	.	.	.	+	.	+	+	.	4
*Trifolium nigrescens*	.	.	.	.	+	1	+	+	.	4
*Cerastium semidecandrum*	.	.	.	.	+	1	+	+	.	4
*Allium subhirsutum*	+	1	1	.	.	.	.	.	.	3

**Localities and dates of relevés.** Rel. 1–9: Pantelleria Island, Brullo et al. [81]—Table 12.

**Table A5:** *Crassulo-Elatinetum gussonei* Bartolo, Brullo, Minissale & Spampinato 1990.

Relevé number	1	2	3	4	5	6	7	8	9	Presences
Altitude (m)	20	20	25	22	30	20	20	30	30	
Surface (m^2^)	0.5	0.5	4	1	2	1	1	0.5	1	
Coverage (%)	50	20	50	60	70	80	70	80	50	
**Char. Association**										
*Elatine gussonei*	1	+	2	3	+	3	2	4	3	9
**Char. All. (ISOËTION) and Ord. (ISOËTETALIA)**										
*Bulliarda vaillantii*	2	+	3	1	1	3	3	1	+	9
**Char. Class (ISOËTO-NANOJUNCETEA)**										
*Lythrum hyssopifolia*	3	2	1	2	3	2	1	1	1	9
*Juncus bufonius*	.	+	+	1	2	1	2	1	.	7
*Polypogon subspathaceus*	+	.	+	1	2	2	2	.	.	6
*Spergularia rubra*	.	.	+	+	2	.	.	.	.	3
*Poa infirma*	.	.	.	.	.	.	1	.	.	1
**Other species**										
*Plantago coronopus*	+	1	+	2	1	+	1	.	.	7
*Cichorium pumilum*	.	.	.	1	+	.	.	.	.	2
*Bromus hordeaceus*	.	.	.	+	1	.	.	.	.	2
*Parapholis incurva*	1	.	.	.	.	.	.	.	.	1
*Asteriscus aquaticus*	.	.	1	.	.	.	.	.	.	1
*Triglochin barrelieri*	.	.	.	1	.	.	.	.	.	1
*Medicago polymorpha*	.	.	.	+	.	.	.	.	.	1

**Localities and dates of relevés.** Rel. 1–9: Lampedusa Island, Bartolo et al. [88]—Table 25.

**Table A6:** *Lythro hyssopifoliae-Elatinetum macropodae* Brullo, Sciandrello, Tavilla & Minissale ass. nov.

Relevé number	1	2	3	4	5	6	7	8	9	10	11	12	13	14	15	16	17	18	19	20	21	22	23	24	25	Presences
Altitude (m)	420	420	440	430	410	450	530	550	560	46	45	36	33	33	32	35	37	33	34	36	240	240	240	240	240	
Surface (m^2^)	2	2	1	3	2	1	1	1	1.5	0.5	0.3	1	0.2	0.3	0.2	1	0.2	1	1	0.2	0.5	0.5	0.5	0.5	1	
Coverage (%)	100	100	90	100	100	80	90	70	80	90	100	100	80	80	80	70	100	100	100	100	100	40	70	100	90	
**Char. Association**																										
*Elatine macropoda*	1	2	1	+	2	2	5	3	2	3	5	5	3	4	4	3	5	5	5	5	3	2	3	4	1	25
**Char. Subassociation**																										
*Callitriche brutia*	.	.	.	.	.	.	.	.	.	.	.	.	.	.	.	.	.	.	.	.	1	1	2	2	1	5
**Char. All. (ISOËTION) and Ord. (ISOËTETALIA)**																										
*Buillardia vaillantii*	5	5	4	5	5	4	2	3	4	.	1	.	2	1	1	1	.	.	.	1	4	3	3	3	4	20
*Catabrosa aquatica*	+	.	+	.	.	.	+	.	.	.	.	.	.	.	.	.	.	.	.	.	.	.	.	.	.	3
*Romulea ramiflora*	.	.	.	.	.	.	.	+	.	.	.	.	.	.	.	.	.	.	.	.	.	.	.	.	.	1
**Char. Class (ISOËTO-NANOJUNCETEA)**																										
*Juncus bufonius*	1	2	1	2	2	1	+	1	1	.	.	.	.	.	.	.	.	.	.	.	3	.	2	+	1	13
*Lythrum hyssopifolia*	3	1	2	+	1	+	.	1	1	3	1	1	1	1	1	2	1	1	1	.	2	1	.	.	1	21
*Poa infirma*	1	+	1	+	1	+	1	2	2	.	.	.	.	.	.	.	.	.	.	.	.	.	.	.	2	10
*Mentha pulegium*	2	+	2	2	1	1	.	1	1	.	.	.	.	.	.	.	.	1	.	.	.	.	.	.	.	9
*Spergularia rubra*	+	1	+	.	1	+	.	.	.	.	.	.	.	.	.	.	.	.	.	.	.	.	.	.	.	5
*Polypogon subspathaceus*	1	+	.	.	.	.	.	+	.	.	.	.	.	.	.	1	.	1	1	.	.	.	.	.	.	6
*Gaudinia fragilis*	1	.	.	.	1	.	1	.	.	.	.	.	.	.	.	.	.	.	.	.	.	.	.	.	.	3
*Juncus foliosus*	.	.	.	.	.	.	.	.	.	.	1	.	.	.	.	1	.	.	.	.	.	.	.	.	.	2
**Other species**																										
*Trifolium resupinatum*	+	+	.	.	.	+	.	.	.	.	.	.	.	.	.	.	.	.	.	.	.	.	.	.	+	4
*Chamaemelum fuscatum*	+	+	.	+	.	+	.	.	.	.	.	.	.	.	.	.	.	.	.	.	.	.	.	.	.	4
*Parapholis cylindrica*	.	.	.	.	.	.	.	.	.	.	.	.	.	.	.	.	.	.	.	.	1	.	.	+	2	3
*Cerastium glomeratum*	.	.	.	.	.	.	+	1	2	.	.	.	.	.	.	.	.	.	.	.	.	.	.	.	.	3
*Persicaria lapathifolia*	+	.	.	.	+	+	.	.	.	.	.	.	.	.	.	.	.	.	.	.	.	.	.	.	.	3
*Lythrum junceum*	1	.	.	1	.	.	.	.	.	.	.	.	.	.	.	.	.	.	.	.	.	.	.	.	.	2
*Glyceria spicata*	1	.	1	.	.	.	.	.	.	.	.	.	.	.	.	.	.	.	.	.	.	.	.	.	.	2
*Sedum rubens*	.	.	.	.	.	.	+	.	+	.	.	.	.	.	.	.	.	.	.	.	.	.	.	.	.	2
*Trisetaria aurea*	.	+	.	.	.	.	.	+	.	.	.	.	.	.	.	.	.	.	.	.	.	.	.	.	.	2
*Callitriche truncata*	.	.	.	.	.	.	.	.	.	.	.	.	.	.	.	.	1	.	1	.	.	.	.	.	.	2
*Eleocharis palustris*	.	2	+	.	.	.	.	.	.	.	.	.	.	.	.	.	.	.	.	.	.	.	.	.	.	2
*Trifolium nigrescens*	.	.	.	+	.	.	.	.	.	.	.	.	.	.	.	.	.	.	.	.	.	.	.	.	.	1
*Vulpia geniculata*	.	.	.	+	.	.	.	.	.	.	.	.	.	.	.	.	.	.	.	.	.	.	.	.	.	1
*Plantago coronopus*	.	.	.	.	.	.	+	.	.	.	.	.	.	.	.	.	.	.	.	.	.	.	.	.	.	1
*Catapodium rigidum*	.	.	.	.	.	.	.	.	.	.	.	.	.	.	.	.	.	.	.	.	+	.	.	.	.	1
*Polygonum aviculare*	.	.	.	.	.	.	.	.	.	.	.	.	.	.	.	.	.	.	.	.	+	.	.	.	.	1
*Symphyotrichum squamatum*	.	.	.	.	.	.	.	.	.	.	.	.	.	.	.	.	.	.	.	1	.	.	.	.	.	1

**Localities and dates of relevés.** Rel. 1–6: Modica plateau—21 April 1984; rel. 7–9: Ragusa plateau—12 May 1977; rel. 10–20: Syracuse, Minissale & Sciandrello [93]—Table S2, rel. 15, 23, 25-28, 32, 36, 43, 45, 50; rel. 21–25: Cava Grande (Rosolini)—28 March 2013.

**Table A7:** *Bulliardo-Elatinetum campylospermae* Brullo, Sciandrello, Minissale, Cambria, Ilardi & Giusso ass. nov.

Relevé number	1	2	3	4	5	6	7	8	9	10	11	12	13	14	15	Presences
Altitude (m)	20	20	25	30	30	25	4	4	92	140	153	4	4	80	80	
Surface (m^2^)	1	1	1	1	1	1	1	1	0.5	0.5	1	1	1	1	1	
Coverage (%)	80	90	70	90	40	60	40	30	100	90	70	80	90	60	80	
**Char. Association**																
*Elatine campylosperma*	1	1	2	1	+	3	2	2	5	5	3	2	3	3	4	15
**Char. All. (ISOËTION) and Ord. (ISOËTETALIA)**																
*Bulliarda vaillantii*	4	5	3	3	3	1	+	+	.	1	1	3	2	1	1	14
*Isolepis cernua*	.	.	.	.	.	.	2	2	.	.	.	.	.	.	.	2
*Damasonium bourgaei*	.	.	.	.	.	.	.	.	.	.	.	+	+	.	.	2
*Triglochin laxiflora*	.	.	.	.	.	.	.	.	.	.	+	.	.	.	.	1
**Char. Class (ISOËTO-NANOJUNCETEA)**																
*Lythrum hyssopifolia*	1	+		+	.	.	1	+	+	.	+	2	+	+	+	12
*Juncus bufonius*	1	2	+	1	1	+	+	1	.	.	.	.	.	.	.	8
*Poa infirma*	3	3	2	2	+	.	.	.	.	.	.	.	.	1	+	7
*Juncus capitatus*	+	2	1	2	.	.	1	+	.	.	.	.	.	.	.	6
*Callitriche brutia*	1	.	2	1	.	3	.	.	.	.	.	.	.	+	2	6
*Mentha pulegium*	2	1	3	2	.	+	.	.	.	.	.	.	.	.	.	5
*Juncus hybridus*	.	.	.	.	.	.	.	.	1	+	.	.	+	+	+	5
*Polypogon subspathaceus*	+	.	1	+	.	.	2	1	.	.	.	.	.	.	.	5
*Gaudinia fragilis*	+	.	.	.	.	.	.	.	.	.	.	.	.	.	.	1
**Other species**																
*Plantago coronopus*	+	.	.	.	+	.	1	2	.	.	.	.	.	.	.	4
*Sedum caeruleum*	.	.	.	+	.	.	.	.	.	1	.	.	.	+	+	4
*Poa bulbosa*	+	1	.	.	.	+	.	.	.	.	.	.	.	.	.	3
*Trifolium resupinatum*	.	+	.	+	.	.	.	.	.	.	.	.	.	.	.	2
*Chamaemelum fuscatum*	.	.	.	.	.	.	.	.	.	1	+	.	.	.	.	2
*Lythrum junceum*	+	.	+	.	.	.	.	.	.	.	.	.	.	.	.	2
*Bromus hordeaceus*	.	.	+	+	.	.	.	.	.	.	.	.	.	.	.	2
*Hypericum pubescens*	.	+	1	.	.	.	.	.	.	.	.	.	.	.	.	2
*Bellis annua*	.	.	+	.	.	.	.	.	.	.	.	.	.	.	.	1
*Trifolium scabrum*	.	.	.	.	.	+	.	.	.	.	.	.	.	.	.	1
*Sedum rubens*	.	.	.	.	.	+	.	.	.	.	.	.	.	.	.	1
*Trisetaria aurea*	.	.	.	.	.	+	.	.	.	.	.	.	.	.	.	1
*Plantago afra*	.	.	.	.	.	.	.	.	.	1	.	.	.	.	.	1

**Localities and dates of relevés.** Rel. 1–6: Favignana (Aegadian Islands)—14 April 1973; rel. 7–8: Isola Grande dello Stagnone (Marsala)—24 April 1994; rel. 9–11: Castello della Pietra (Castelvetrano), Pasta et al. [128]—Table 7; rel. 12–13: Isola Lunga dello Stagnone—28 March 2010; rel. 14–15: Trapani Vallone della Pietra—1 April 2021.

**Table A8:** *Isoëtetum todaroanae* Brullo & Ilardi ass. nova.

Relevé number	1	2	3	4	5	Presences
Altitude (m)	60	60	60	60	60	
Surface (m^2^)	2	2	3	1	2	
Coverage (%)	90	90	80	90	80	
**Char. Association**						
*Isoëtes todaroana*	3	2	3	3	1	5
**Char. All. (ISOËTION) and Ord. (ISOËTETALIA)**						
*Triglochin laxiflora*	3	3	2	3	3	5
*Romulea ramiflora*	2	2	1	2	2	5
*Isolepis cernua*	1	2	1	1	2	5
**Char. Class (ISOËTO-NANOJUNCETEA)**						
*Mentha pulegium*	3	3	3	3	2	5
*Lythrum hyssopifolia*	3	3	3	2	3	5
*Juncus bufonius*	2	2	1	3	2	5
**Other species**						
*Carex serrulata*	1	1	+	1	+	5
*Bolboschoenus maritimus*	1	2	+	1	1	5
*Blackstonia acuminata*	1	1	+	1	+	5
*Linum bienne*	2	1	1	2	2	5
*Eleocharis palustris*	+	+	.	.	+	3
*Oenanthe pimpinelloides*	.	+	+	.	.	2

**Localities and dates of relevés.** Rel. 1–5: Contrada Critazzo (Mazara del Vallo)—4 April 2013.

**Table A9:** Isoëto velatae - Crassuletum vaillantii Poiron & Barbero 1965.

Relevé number	1	2	3	4	5	6	7	8	9	10	11	12	13	14	15	16	Presences
Altitude (m)	360	360	360	360	386	360	360	360	230	230	230	230	230	230	230	230	
Surface (m^2^)	1.6	1.6	1.6	1.6	1.6	1	1	1	1	3	1	2	4	2	2	3	
Coverage (%)	85	80	85	90	80	80	70	80	100	100	80	80	80	100	90	90	
**Char. Association**																	
*Isoëtes longissima*	3	2	4	4	4	1	3	3	3	5	3	2	3	3	3	3	16
**Char. Subassociation**																	
*Ranunculus ophioglossifolius*	.	.	.	.	.	.	.	.	.	.	.	.	4	3	3	1	4
*Warnstorfia fluitans*	.	.	.	.	.	.	.	.	.	.	.	.	3	2	2	3	4
**Char. All. (PRESLION CERVINAE)**																	
*Callitriche brutia*	.	.	1	.	.	3	2	3	4	1	1	.	3	2	2	.	10
*Ranunculus lateriflorus*	1	1	3	2	1	2	2	3	.	.	.	.	.	.	.	.	8
*Pilularia minuta*	2	1	.	1	.	1	2	2	.	.	.	.	.	.	.	.	6
**Char. Ord. (ISOËTETALIA)**																	
*Bulliarda vaillantii*	2	1	1	2	1	3	2	2	1	2	1	3	.	1	1	.	14
*Lotus angustissimus*	+	1	.	1	.				.	+	.	+	1	2	1	1	9
*Solenopsis laurentia* subsp. *hyblaea*	.	.	.	.	.	.	.	.	.	.	.	.	1	+	.	1	3
*Isoëtes durieui*	.	.	.	.	.	2	.	.	.	.	.	.	.	.	.	.	1
**Char. Class (ISOËTO-NANOJUNCETEA)**																	
*Lythrum hyssopifolia*	+	1	1	1	1	1	1	1	1	2	2	2	2	3	1	1	16
*Poa infirma*	1	1	.	1	.	2	1	1	1	2	2	3	2	2	3	1	14
*Juncus hybridus*	1	1	.	+	.	2	1	2	1	1	1	1	2	1	1	2	14
*Mentha pulegium*	.	.	.	.	1	1	1	1	1	2	.	1	2	2	1	2	11
*Juncus pygmaeus*	.	.	.	.	1	1	1	1	1	.	.	.	1	+	+	1	9
*Romulea ramiflora*	.	.	.	.	.	.	.	.	1	2	2	2	2	1	2	+	8
*Eryngium pusillum*	1	1	.	1	.	1	1	1	.	.	.	.	.	.	.	.	6
*Archidium alternifolium*	.	.	.	.	.	.	.	.	.	2	2	1	1	1	1	.	6
*Juncus capitatus*	.	.	.	.	.	.	.	.	1	+	.	+	+	+	+	.	6
*Lotus parviflorus*	.	.	.	.	.	.	.	.	.	.	.	1	2	1	1	.	4
*Pulicaria vulgaris* var. *vulgaris*	.	.	.	.	.	1	+	+	.	.	.	.	.	.	.	.	3
*Isolepis cernua*	.	.	.	.	.	.	.	.	.	.	.	.	+	.	.	1	2
*Briza minor*	.	.	.	.	.	.	.	.	.	.	.	.	.	.	.	1	1
**Other species**																	
*Trifolium resupinatum*	.	+	.	1	.	.	.	+	.	.	.	+	+	1	1	4	8
*Bellis annua*	.	.	.	.	.	.	.	.	1	1	1	1	2	2	1	.	7
*Glyceria spicata*	.	.	1	1	1	1	2	1	.	.	.	.	.	.	.	.	6
*Polygonum arenastrum*	.	.	.	.	.	1	1	1	2	+	.	.	.	.	+		6
*Anthemis cotula*	.	+	+	.	+	1	.	1	.	.	.	.	.	.	.	.	5
*Linum bienne*	.	.	.	.	.	.	.	.	.	+	.	+	.	1	1	1	5
*Coleostephus myconis*	.	.	.	.	.	.	.	.	.	.	.	1	+	1	1	1	5
*Veronica anagallis-aquatica*	1	1	+	.	+	.	.	.	.	.	.	.	.	.	.	.	4
*Eleocharis palustris*	+	.	.	.	.	.	.	1	.	.	.	.	.	.	2	.	3
*Montia arvensis*	.	.	.	.	.	.	.	.	.	.	.	.	2	1	1	.	3
*Carex* sp.	.	.	.	.	.	.	.	.	.	.	.	.	+	1	.	.	2
*Cardamine occulta*	.	.	.	.	.	.	.	.	.	.	3	2	.	.	.	.	2
*Plantago lagopus*	.	.	.	.	.	+	.	.	.	.	.	.	.	.	.	.	1
*Cerastium glomeratum*	.	.	.	.	.	.	.	+	.	.	.	.	.	.	.	.	1
*Vulpia* sp.	.	.	.	.	.	.	.	.	.	.	.	.	.	.	.	2	1
*Anagallis arvensis*		.	.	.	.	.	.	.	.	.	.	.	.	.	.	1	1
*Ornithogalum gussonei*	.	.	.	.	.	.	.	.	.	.	.	.	.	.	.	+	1
*Cyperus* sp.	.	.	.	.	.	.	.	.	.	.	.	.	.	.	.	2	1

**Localities and dates of relevés.** Rel. 1–4: Cozzo Fico (Carlentini), Minissale et al. [132]—Tab.1, rel. 14,16–18; rel. 5: Cozzo Ogliastri (Sortino), Minissale et al. [132]—Table 1, Ril. 19; rel. 6–8: Cozzo Fico (Carlentini)—5 May 2021; rel. 9–15: Curcuraggi (Melilli)—1 April 2021; rel. 16: Curcuraggi (Melilli), 5 May 2021.

**Table A10:** Ranunculo laterifloro-Antinorietum insularis Brullo, Grillo & Terrasi 1976.

Relevé number	1	2	3	4	5	6	7	8	9	Presences
Altitude (dam)	93	92	92	95	93	92	92	92	96	
Surface (m^2^)	2	2	2	2	2	2	2	3	3	
Coverage (%)	90	100	90	90	80	70	90	70	70	
**Char. Association**										
*Bulliarda vaillantii*	.	+	.	+	2	+	2	1	2	7
*Isoëtes sicula*	+	2	1	1	+	.	1	1	.	7
*Myosotis tineoi*	.	4	3	3	2	.	.	.	.	4
**Char. All. (PRESLION CERVINAE)**										
*Antinoria insularis*	3	+	1	1	2	1	2	3	2	9
*Ranunculus lateriflorus*	.	1	+	1	2	+	2	1	3	8
*Callitriche brutia*	.	.	.	.	1	2	2	1	1	5
**Char. Ord. (ISOËTETALIA)**										
*Lotus angustissimus*	+	+	1	+	1	1	.	.	+	7
*Trifolium micranthum*	1	+	.	+	.	1	+	+	.	6
*Ranunculus muricatus*	+	.	.	1	+	2	.	.	+	5
*Isoëtes longissima*	.	+	1	.	1	2	.	.	.	4
*Ranunculus trilobus*	2	.	.	.	.	.	.	+	1	3
*Lotus conimbricensis*	.	1	+	+	.	.	.	.	.	3
**Char. Class (ISOËTO-NANOJUNCETEA)**										
*Mentha pulegium*	2	3	3	2	3	2	3	2	3	9
*Lythrum hyssopifolia*	2	+	1	1	2	+	1	1	+	9
*Gaudinia fragilis*	1	1	2	3	.	.	.	+	1	6
*Eryngium pusillum*	1	.	.	.	1	2	2	2	1	6
*Poa infirma*	.	1	2	2	2	1	.	.	.	5
*Juncus bufonius*	+	.	.	.	.	.	+	.	+	5
*Juncus pygmaeus*	3	.	.	.	.	.	+	+	1	4
*Juncus capitatus*	1	.	.	.	+	.	.	+	+	4
*Polypogon subspathaceus*	.	.	1	1	.	.	.	.	.	2
**Other species**										
*Trifolium tomentosum*	+	.	.	.	.	.	+	.	+	3
*Trifolium squarrosum*	.	+	+	1	.	.	.	.	.	3
*Trifolium leucanthum*	.	+	.	+	.	.	.	.	.	2
*Anagallis arvensis*	.	+	.	.	.	.	.	.	.	1
*Myosotis ramosissima*	.	.	.	.	+	.	.	.	.	1

**Localities and dates of relevés.** Rel. 1–9: Monte Lauro, Brullo et al. [80]—Table 3.

**Table A11:** Myosuro minimi-Ranunculetum lateriflori Raimondo 1980.

Relevé number	1	2	3	4	5	6	7	8	9	10	11	Presences
Altitude (dam)	160	160	160	160	160	160	160	150	160	140	160	
Surface (m^2^)	4	9	2	4	2	3	1	1	2	3	5	
Coverage (%)	90	90	60	80	60	80	80	80	75	70	80	
**Char. Association**												
*Myosurus minimus*	1	1	3	2	2	2	1	+	3	2	1	11
*Spergularia madoniaca*	3	4	2	2	+	1	+	+	+	.	4	10
*Sagina subulata*	+	+	+	.	.	.	.	1	+	+	.	6
*Ranunculus marginatus*	.	.	.	.	.	.	.	.	1	+	.	2
**Char. All. (PRESLION CERVINAE)**												
*Antinoria insularis*	+	+	2	1	+	2	2	+	1	1	.	11
*Ranunculus lateriflorus*	3	2	3	3	1	3	3	+	1	1	4	11
**Char. Ord. (ISOËTETALIA)**												
*Trifolium micranthum*	.	.	.	+	+	.	1	.	+	.	.	5
*Middendorfia borysthenica*	.	.	.	.	.	.	.	.	+	+	.	2
*Lotus angustissimus*	.	.	.	.	.	.	.	.	1	.	.	1
**Char. Class (ISOËTO-NANOJUNCETEA)**												
*Poa infirma*	1	1	.	+	+	+	1	+	1	+	+	10
*Juncus bufonius*	.	.	1	+	+	+	+	1	1	1	.	8
*Mentha pulegium*	.	.	3	3	+	1	+	.	.	+	.	6
**Other species**												
*Plantago cupanii*	.	+	+	1	+	+	.	3	+	+	.	8
*Polygonum aviculare*	2	1	.	.	+	+	.	.	+	+	2	7
*Cynosurus cristatus*	+	+	.	.	.	.	.	.	+	+	.	4
*Eleocharis nebrodensis*	.	.	.	.	.	+	+	.	+	.	.	3
*Montia arvensis*	.	.	.	.	+	.	+	.	+	.	.	3
*Potentilla reptans*	.	.	.	.	.	.	.	.	+	+	.	2
*Moenchia erecta*	.	.	.	.	+	.	+	.	.	.	.	2
*Lolium perenne*	.	+	.	.	2	.	.	.	.	.	.	2
*Trifolium strictum*	.	.	.	.	+	.	.	.	.	.	2	2
*Carex leporina*	.	.	.	.	.	.	.	.	+	+	.	2
*Cerastium semidecandrum*	.	.	.	.	.	.	.	.	+	+	.	2
*Agrostis stolonifera*	.	.	.	.	.	.	.	.	+	+	.	2
*Juncus compressus*	+	.	.	.	.	.	.	.	+	.	.	2
*Carex divisa*	.	.	.	.	.	.	.	.	.	.	+	1
*Bryum alpinum*	.	.	.	.	.	.	.	.	+	.	.	1

**Localities and dates of relevés.** Rel. 1–2: Piano della Battaglietta (Madonie)—20 June 1975; rel. 3–7: Piano Gallo (Geraci)—13 June 1975; rel. 8: Piano Catarineci (Madonie)—13 June 1975; rel. 9–10: Madonie, Raimondo [86]—Table 2; rel. 11: Piano della Battaglia (Madonie)—20 June 1975.

**Table A12:** *Ranunculetum pratensi-lateriflori* Brullo, C. Brullo & Giusso ass. nov.

Relevé number	1	2	3	4	5	6	7	8	9	10	11	Presences
Altitude (dam)	146	146	146	146	146	146	170	170	134	134	165	
Surface (m^2^)	1	0.6	0.6	0.6	0.3	0.2	0.5	0.8	0.5	1.5	2	
Coverage (%)	100	100	100	100	100	100	80	80	80	90	80	
**Char. Association**												
*Ranunculus pratensis*	1	+	1	2	1	1	+	+	+	1	1	11
*Barbarea bracteosa*	+	1	+	+	+	+	+	1	.	.	.	8
*Veronica serpyllifolia*	+	.	+	+	1	+	3	2	.	.	.	7
**Char. All. (PRESLION CERVINAE)**												
*Antinoria insularis*	3	4	1	2	3	1	1	1	1	+	1	11
*Ranunculus lateriflorus*	3	2	3	3	2	3	2	2	1	2	+	11
**Char. Ord. (ISOËTETALIA)**												
*Lotus angustissimus*	+	+	1	1	1	1	+	.	.	1	1	9
*Trifolium micranthum*	1	.	1	.	+	1	1	1	.	.	.	6
*Middendorfia borysthenica*	4	3	3	2	2	3	.	.	.	.	.	6
*Myosotis sicula*	+	1	1	+	+	1	.	.	.	.	.	6
**Char. Class (ISOËTO-NANOJUNCETEA)**												
*Mentha pulegium*	1	+	.	.	+	1	+	1	4	4	+	9
*Poa infirma*	2	1	1	2	+	2	1	.	.	.	.	7
*Juncus bufonius*	.	.	.	+	.	.	.	.	+	+	+	4
**Other species**												
*Spergularia bocconei*	+	2	1	2	1	3	.	+	.	.	+	8
*Oenanthe fistulosa*	1	+	+	1	1	+	.	.	.	.	.	6
*Polypogon viridis*	.	+	.	1	.	+	.	.	2	1	.	5
*Polygonum aviculare*	+	.	.	+	+	.	.	+	.	.	.	4
*Potentilla reptans*	+	.	.	.	+	1	.	+	.	.	.	4
*Helosciadium inundatum*	.	+	+	.	.	1	.	.	.	.	.	3
*Lythrum junceum*	.	.	.	.	.	.	.	1	+	+	.	3
*Cynosurus cristatus*	.	.	.	.	.	.	.	.	+	+	.	2
*Trifolium pratense*	.	.	.	.	.	1	+	.	.	.	.	2
*Oenanthe globulosa*	.	.	.	.	.	.	.	+	.	.	.	1

**Localities and dates of relevés.** Rel. 1–11: Nebrodi mounts, Brullo & Grillo [84]—Table 7.

**Table A13:** Ranunculo lateriflori-Callitrichetum brutiae Brullo & Minissale 1998.

Relevé number	1	2	3	4	5	6	7	Presences
Altitude (m)	980	960	960	960	960	980	980	
Surface (m^2^)	1.5	1	1	2	1	3	2	
Coverage (%)	100	90	95	100	90	100	100	
**Char. Association and All. (PRESLION CERVINAE)**								
*Callitriche brutia*	4	3	3	4	3	1	1	7
*Ranunculus lateriflorus*	+	1	2	3	2	3	4	7
**Char. Ord. (ISOËTETALIA)**								
*Bulliarda vaillantii*	3	3	4	2	2	3	2	7
*Middendorfia borysthenica*	1	+	+	+	.	+	.	5
*Ranunculus trilobus*	.	+	1	1	+	+	.	5
**Char. Class (ISOËTO-NANOJUNCETEA)**								
*Mentha pulegium*	+	2	1	1	3	2	3	7
*Lythrum hyssopifolia*	1	1	2	2	2	1	1	7
*Juncus bufonius*	+	1	2	2	2	+	+	7
*Polypogon subspathaceus*	.	+	1	+	+	+	+	6
*Juncus capitatus*	.	+	1	+	+	.	.	4
*Gaudinia fragilis*	.	+	+	.	+	.	.	3
*Poa infirma*	.	+	.	+	+	.	.	3
**Other species**								
*Polygonum aviculare*	.	1	1	+	1	1	1	6
*Trifolium tomentosum*	.	+	.	+	+	+	1	6
*Spergularia bocconei*	+	+	1	1	+	.	.	5

**Localities and dates of relevés.** Rel. 1–7: Monte Lauro, Brullo et al. [80]—Table 3, rel. 18–24.

**Table A14:** *Callitricho brutiae-Crassuletum vaillantii* Brullo, Scelsi, Siracusa & Tomaselli 1998.

Relevé number	1	2	3	4	5	Presences
Altitude (dam)	52	52	52	51	51	
Surface (m^2^)	2	2	3	2	1	
Coverage (%)	90	100	90	90	90	
**Char. Association and All. (PRESLION CERVINAE)**						
*Callitriche brutia*	5	5	3	3	3	5
**Char. Ord. (ISOËTETALIA)**						
*Bulliarda vaillantii*	4	3	3	3	3	5
*Lotus angustissimus*	+	1	1	+	.	4
*Isoëtes durieui*	1	1	3	2	.	4
*Isoëtes longissima*	.	.	.	.	4	1
*Romulea ramiflora*	.	.	2	.	.	1
**Char. Class (ISOËTO-NANOJUNCETEA)**						
*Mentha pulegium*	3	3	2	3	2	5
*Juncus bufonius*	1	2	2	2	1	5
*Lythrum hyssopifolia*	2	3	3	1	2	5
*Polypogon subspathaceus*	2	1	2	2	1	5
*Poa infirma*	1	1	2	+	1	5
*Juncus capitatus*	+	1	2	1	.	4
*Juncus pygmaeus*	.	.	.	.	2	1
**Other species**						
*Coleostephus myconis*	+	1	1	1	+	5
*Bellis annua*	+	+	1	.	.	3
*Juncus articulatus*	.	.	.	.	2	1
*Linum bienne*	.	.	.	+	.	1

**Localities and dates of relevés.** Rel. 1–5: Bosco Pisano (Buccheri), Brullo et al. [83]—Table 2.

**Table A15:** *Junco pygmaei-Pilularietum minutae* Minissale, Molina & Sciandrello 2017.

Relevé number	1	2	3	4	5	6	7	8	9	10	11	12	13	14	Presences
Altitude (m)	384	384	384	384	386	386	360	360	360	386	386	386	386	386	
Surface (m^2^)	1.6	1.6	1.6	1.6	1.6	1.6	1	1	1	1.6	1.6	1.6	1.6	1.6	
Coverage (%)	70	50	85	90	85	90	80	70	80	60	80	80	85	90	
**Char. Association**															
*Pilularia minuta*	2	1	3	4	3	4	1	2	2	3	2	3	1	1	14
**Char. Subassociation**															
*Solenopsis laurentia* subsp. *hyblaea*	.	.	.	.	.	.	.	.	.	1	1	1	2	2	5
*Cicendia filiformis*	.	.	.	.	.	.	.	.	.	+	1	1	1	+	5
**Char. All. (PRESLION CERVINAE)**															
*Callitriche brutia*	4	3	2	1	+	.	3	2	3	.	.	.	.	.	8
*Ranunculus lateriflorus*	.	.	.	.	.	.	2	2	3	1	1	1	2	2	8
**Char. Ord. (ISOËTETALIA)**															
*Isoëtes longissima*	+	.	.	.	1	1	1	3	3	1	1	1	2	2	11
*Bulliarda vaillantii*	1	1	1	1	1	+	3	2	2	.	.	.	.	.	9
*Lotus angustissimus*	.	.	.	.	.	.				1	1	+	1	1	5
*Isoetes durieui*	.	.	.	.	.	.	2	.	.	.	.	.	.	.	1
**Char. Class (ISOËTO-NANOJUNCETEA)**															
*Juncus pygmaeus*	1	.	1	1	3	2	1	1	1	2	3	2	3	3	13
*Lythrum hyssopifolia*	1	.	+	1	1	2	1	1	1	2	1	1	1	1	13
*Mentha pulegium*	.	.	+	+	1	+	1	1	1	1	1	1	1	1	12
*Juncus hybridus*	.	.	+	+	1	1	2	1	2	1	1	1	1	1	12
*Poa infirma*	.	.	+	.	+	+	2	1	1	.	.	.	.	.	6
*Eryngium pusillum*	.	.	.	.	.	.	1	1	1	.	.	.	.	.	3
*Polypogon subspathaceus*	.	.	.	.	.	.	2	1	1	.	.	.	.	.	3
*Pulicaria vulgaris* var. *vulgaris*	.	.	.	.	.	.	1	+	+	.	.	.	.	.	3
**Other species**															
*Polygonum arenastrum*	.	.	+	.	.	.	1	1	1	.	.	.	.	.	4
*Anthemis cotula*	.	.	.	.	.	.	1	.	1	.	.	.	.	.	2
*Eleocharis palustris*	.	.	.	.	.	.	.	.	1	+	.	.	.	.	2
*Spergularia sp.*	.	.	1	.	.	.	.	.	.	.	.	.	.	.	1
*Trifolium resupinatum*	.	.	.	.	.	.	.	.	+	.	.	.	.	.	1
*Plantago lagopus*	.	.	.	.	.	.	+	.	.	.	.	.	.	.	1
*Cerastium glomeratum*	.	.	.	.	.	.	.	.	+	.	.	.	.	.	1

**Localities and dates of relevés.** Rel. 1–6: Cozzo Ogliastri (Sortino), Minissale et al. [132]—Table 1, rel. 1–4, 22-23; rel. 7–9: Cozzo Fico (Carlentini)—5 May 2021; rel. 10–14: Cozzo Ogliastri (Sortino), Minissale et al. [132]—Table 1, rel. 6, 8, 10, 12, 20.

**Table A16:** *Pilulario minutae-Myosotidetum siculae* Brullo, Cambria, Ilardi & Minissale ass. nov.

Relevé number	1	2	Presences
Altitude (m)	205	205	
Surface (m^2^)	5	5	
Coverage (%)	100	100	
**Char. Association**			
*Pilularia minuta*	1	1	2
*Myosotis sicula*	3	4	2
**Char. All. (PRESLION CERVINAE)**			
*Ranunculus ophioglossifolius*	2	3	2
**Char. Ord. (ISOËTETALIA)**			
*Isoëtes longissima*	2	2	2
*Lotus parviflorus*	1	2	2
*Elatine macropoda*	1	+	2
*Romulea ramiflora*	1	2	2
**Char. Class (ISOËTO-NANOJUNCETEA)**			
*Polypogon subspathaceus*	2	2	2
**Other species**			
*Eleocharis palustris*	3	3	2
*Alisma lanceolatum*	2	1	2
*Ranunculus peltatus*	2	2	2
*Glyceria notata*	4	4	2
*Carex divisa*	1	1	2

**Localities and dates of relevés.** Rel. 1–2: Contrada Anguillara (Calatafimi)—28 April 2017.

**Table A17:** Archidio phascoidis-Isoëtetum velatae Brullo & Minissale 1998.

Relevé number	1	2	3	4	5	6	7	8	Presences
Altitude (m)	385	385	385	385	385	230	230	230	
Surface (m^2^)	1	2	1	1	1	3	1	1	
Coverage (%)	100	100	100	100	90	90	70	70	
**Char. Association**									
*Archidium alternifolium*	2	1	1	2	3	2	2	2	8
**Char. All. (CICENDIO-SOLENOPSION LAURENTIAE)**									
*Solenopsis laurentia* subsp. *hyblaea*	1	+	1	1	2	2	2	2	8
*Anagallis parviflora*	+	+	+	+	+	.	.	.	5
**Char. Ord. (ISOËTETALIA)**									
*Isoëtes longissima*	4	2	4	4	4	1	3	2	8
*Triglochin laxiflora*	1	2	1	1	1	1	.	.	6
*Isoëtes durieui*	1	.	1	2	.	2	.	+	5
*Isoëtes histrix*	.	+	+	1	+	.	.	.	4
*Centaurium maritimum*	1	+	.	.	+	.	.	.	3
*Bulliarda vaillantii*	.	.	.	.	.	4	2	3	3
**Char. Class (ISOËTO-NANOJUNCETEA)**									
*Juncus bufonius*	3	3	1	1	2	2	1	1	8
*Lythrum hyssopifolia*	+	.	+	1	1	2	2	3	7
*Juncus pygmaeus*	.	1	1	+	1	2	1	.	6
*Juncus capitatus*	1	.	+	+	1	2	2	.	6
*Mentha pulegium*	.	1	1	1	2	2	.	.	5
*Poa infirma*	.	.	.	.	.	2	2	1	3
*Romulea ramiflora*	.	.	.	.	.	1	2	1	3
*Lotus parviflorus*	.	.	.	.	.	1	1	1	3
*Callitriche brutia*	.	.	.	.	.	2	1	.	2
*Lotus angustissimus*	.	.	.	.	.	.	+	+	2
*Ranunculus ophioglossifolius*	.	.	.	.	.	+	.	.	1
*Ranunculus sardous*	.	.	.	.	.	.	.	+	1
**Other species**									
*Oenanthe pimpinelloides*	1	1	1	1	1	.	.	.	5
*Ranunculus paludosus*	2	1	1	2	+	.	.	.	5
*Bellis annua*	.	.	1	+	.	1	1	+	5
*Bryum* sp.	2	1	+	2	.	.	.	.	4
*Aira cupaniana*	1	1	.	1	+	.	.	.	4
*Centaurium tenuiflorum*	.	+	+	.	+	.	.	.	3
*Pleurochaete squarrosa*	2	1	.	1	.	.	.	.	3
*Polygonum aviculare*	.	.	.	.	.	2	.	1	2
*Linum bienne*	.	.	.	.	.	2	.	+	2
*Cardamine occulta*	.	.	.	.	.	.	+	+	2
*Blackstonia perfoliata*	1	+	.	.	.	.	.	.	2
*Coleostephus myconis*	.	.	.	.	.	.	1	+	2

**Localities and dates of relevés.** Rel. 1–4: Cozzo Ogliastri (Sortino)—29 April 1992; rel. 5: Cozzo Ogliastri (Sortino), Brullo & Minissale [39], rel. pg. 283; rel. 6–8: Curcuraggi (Melilli)—1 April 2021.

**Table A18:** *Anagallido parviflorae-Molinerielletum minutae* Brullo, Scelsi, Siracusa & Tomaselli 1998.

Relevé number	1	2	3	4	5	6	7	8	9	10	11	12	13	14	15	16	17	18	19	20	21	22	23	24	Presences
Altitude (m)	530	530	500	500	500	510	510	520	520	520	520	520	530	530	530	510	510	500	500	500	530	530	500	500	
Surface (m^2^)	2	2	2	2	2	3	2	1	2	1	2	1	1	1	2	1	1	1	2	1	1	1	2	1	
Coverage (%)	70	70	70	80	40	80	70	80	80	80	60	50	80	80	90	90	70	90	90	80	80	80	90	50	
**Char. Association**																									
*Molineriella minuta*	2	3	2	2	+	2	2	2	+	+	1	2	2	1	1	2	3	2	2	2	1	2	1	1	24
**Char. All. (CICENDIO-SOLENOPSION LAURENTIAE)**																									
*Anagallis parviflora*	2	2	2	2	1	2	2	1	1	+	2	1	2	+	2	+	1	+	2	+	2	1	2	1	24
**Char. Ord. (ISOËTETALIA)**																									
*Isoëtes durieui*	2	2	2	2	1	2	1	1	2	+	1	1	1	2	2	2	1	2	2	1	2	2	1	1	24
*Romulea ramiflora*	+	.	.	1	+	1	.	+	.	+	+	.	+	+	+	1	1	1	+	1	1	+	1	+	20
*Lotus angustissimus*	1	1	+	2	.	2	2	.	1	.	+	+	.	+	2	1	1	2	1	1	1	1	+	.	19
*Lotus conimbricensis*	+	.	1	1	1	1	.	1	+	1	1	1	.	1	1	+	.	+	1	+	1	1	.	1	19
*Bulliarda vaillantii*	.	.	.	+	+	.	+	+	1	+	1	.	.	1	.	.	.	1	2	3	4	2	4	3	15
*Centaurium maritimum*	.	.	+	+	+	.	.	.	.	+	+	1	.	.	.	+	+	1	.	+	.	.	.	.	10
**Char. Class (ISOËTO-NANOJUNCETEA)**																									
*Lythrum hyssopifolia*	1	1	1	3	2	3	2	1	2	1	+	1	2	2	2	3	1	2	3	2	3	2	1	1	24
*Juncus bufonius*	3	2	3	2	1	2	3	2	2	1	1	2	3	2	1	2	2	2	2	1	2	2	1	1	24
*Juncus capitatus*	1	2	2	1	+	1	1	1	2	2	2	1	1	1	2	1	+	1	2	1	2	2	1	1	24
*Polypogon subspathaceus*	1	1	2	2	2	2	2	2	2	2	2	2	2	2	2	3	3	2	3	2	2	2	1	2	24
*Poa infirma*	2	2	2	1	+	1	2	2	3	1	1	1	2	2	2	.	.	1	2	+	3	2	1	1	22
*Juncus pygmaeus*	+	+	1	1	.	.	1	1	+	+	+	+	.	.	+	+	1	1	+	+	+	1	.	.	18
*Juncus hybridus*	.	.	.	.	.	.	.	.	.	.	.	.	.	.	.	.	1	+	+	.	.	.	.	.	3
**Other species**																									
*Coleostephus myconis*	2	2	2	2	2	1	1	3	3	2	2	1	2	.	+	2	2	1	2	1	2	1	+	+	23
*Bellis annua*	2	2	2	2	+	2	2	1	+	+	1	1	+	2	2	.	+	.	1	+	1	1	+	2	22
*Euphorbia exigua*	1	1	1	1	1	+	.	1	+	+	+	+	1	+	1	.	+	.	+	+	.	.	1	1	19
*Medicago turbinata*	+	1	1	1	+	1	1	.	1	+	1	+	1	+	1	.	.	+	1	.	1	.	+	1	19
*Plantago lagopus*	+	1	1	.	2	.	+	1	+	.	2	2	.	2	1	.	.	.	+	+	1	2	1	.	16
*Allium obtusiflorum*	+	1	1	1	+	1	1	+	+	+	.	+	+	.1	.	+	.	1	+	.	.	.	.	.	15
*Scorpiurus muricatus*	.	.	.	.	1	.	.	+	1	1	1	1	+	1	+	.	.	.	+	.	+	1	2	1	15
*Ranunculus paludosus*	.	.	+	.	1	.	+	+	.	.	2	1	+	+	.	.	.	.	+	+	+	+	.	1	13
*Moenchia arrecta*	+	.	.	+	.	.	.	.	+	+	+	1	.	1	+	.	.	.	.	.	+	+	1	+	12
*Sagina apetala*	1	1	1	1	1	1	.	.	.	+	+	.	1	+	.	.	.	.	.	.	.	1	+	.	12
*Logfia gallica*	+	+	+	.	+	.	+	+	.	.	+	1	+	.	.	.	.	.	+	+	+	.	.	.	12
*Poa bulbosa*	.	+	+	1	.	1	1	+	1	1	+	.	+	.	1	.	.	.	.	.	.	.	.	.	11
*Linum bienne*	.	.	.	+	1	1	+	.	.	+	.	.	+	.	.	+	.	+	+	+	.	.	.	.	10
*Paronychia echinulata*	+	.	.	+	.	+	.	.	.	.	.	.	.	1	+	.	.	+	.	.	+	1	1	1	10
*Anthemis arvensis*	+	.	+	.	+	.	.	+	+	.	1	.	.	.	+	+	.	1	1	.	.	.	.	.	10
*Gynandriris sysirinchium*	.	.	.	+	1	+	.	.	.	.	1	.	+	1	.	.	.	.	.	.	.	1	+	.	8
*Tillaea muscosa*	.	.	.	.	.	.	.	.	.	.	+	1	.	+	.	.	.	.	.	.	.	1	2	2	6
*Hypochoeris achyrophorus*	.	.	.	.	.	.	.	.	.	.	+	+	+	.	.	+	.	+	.	.	.	.	.	+	6
*Biscutella maritima*	.	.	.	.	.	.	.	.	.	.	.	+	+	.	.	.	.	.	.	.	.	+	.	.	3
*Parentucellia latifolia*	.	.	.	.	1	.	.	.	.	.	.	1	.	.	.	.	.	+	.	.	.	.	.	.	3
*Saxifraga tridactylites*	.	.	.	.	+	.	.	.	.	.	.	.	.	.	.	.	.	.	.	.	.	.	.	.	1
*Silene gallica*	.	.	.	.	.	.	.	.	.	.	.	.	.	.	.	.	.	.	.	.	.	.	.	1	1

**Localities and dates of relevés.** Rel. 1–24: Bosco Pisano (Buccheri), Brullo et al. [83]—Table 1.

**Table A19:** Kickxio cirrhosae-Solenopsietum gasparrinii Brullo & Minissale 1998 corr.

Relevé number	1	2	3	4	5	6	7	8	9	10	11	12	Presences
Altitude (m)	2	2	2	2	2	2	2	2	2	2	2	2	
Surface (m^2^)	1	1	0.6	0.6	0.5	1	2	2	2	0.5	5	2	
Coverage (%)	50	70	70	70	90	90	70	90	90	80	100	90	
**Char. Association**													
*Kickxia cirrhosa*	1	1	2	2	1	2	1	1	+	+	+	1	12
**Char. All. (CICENDIO-SOLENOPSION LAURENTIAE)**													
*Solenopsis gasparrinii*	+	2	1	2	2	1	2	2	3	3	1	+	12
*Cicendia filiformis*	2	3	1	2	2	2	2	2	1	+	1	2	12
*Radiola linoides*	3	3	3	2	4	4	3	3	2	3	.	+	11
*Anagallis minima*	+	.	+	.	+	+	1	1	+	+	.	1	9
*Anagallis parviflora*	.	.	+	+	.	1	1	2	2	+	1	1	9
*Ophioglossum lusitanicum*	1	.	1	+	.	+	.	.	+	.	.	+	6
**Char. Ord. (ISOËTETALIA)**													
*Lotus parviflorus*	+	1	2	1	1	+	1	+	+	.	4	1	11
*Centaurium maritimum*	1	1	+	1	1	2	1	1	2	+	.	+	11
*Briza minor*	.	.	.	.	.	.	.	+	1	.	.	.	2
*Isoëtes histrix*	.	.	.	.	.	.	.	.	.	.	.	1	1
**Char. Class (ISOËTO-NANOJUNCETEA)**													
*Juncus capitatus*	1	1	2	2	2	1	2	3	2	1	1	1	12
*Lythrum hyssopifolia*	+	+	.	.	+	.	+	2	2	2	2	2	9
*Polypogon subspathaceus*	.	+	.	+	+	.	+	2	3	1	2	1	9
*Juncus bufonius*	1	1	2	1	+	+	.	+	.	.	.	1	8
*Gaudinia fragilis*	.	.	.	.	.	1	+	.	+	1	.	.	4
**Other species**													
*Euphorbia pterococca*	.	.	+	+	+	+	1	.	.	.	+	+	7
*Anthoxantum odoratum*	.	.	.	.	.	.	.	.	.	.	1	+	2
*Coleostephus myconis*	.	.	.	.	.	.	.	.	.	.	+	1	2
*Trifolium campestre*	.	.	.	.	.	.	.	.	.	.	+	+	2
*Triglochin barrelieri*	.	.	.	.	.	.	.	.	.	.	+	.	1

**Localities and dates of relevés.** Rel. 1–10: Isola Grande dello Stagnone (Marsala), Brullo et al. [134]—Tab.11; rel. 11–12: Isola Grande dello Stagnone (Marsala)—28 March 2010.

**Table A20:** *Solenopsietum mothianae* Brullo, Giusso, Minissale & Sciandrello ass. nov.

Relevé number	1	2	3	4	5	Presences
Altitude (m)	1	1	1	1	1	
Surface (m^2^)	2	2	1	1	2	
Coverage (%)	40	50	30	30	40	
**Char. Association**						
*Solenopsis mothiana*	2	2	3	1	2	5
**Char. All. (CICENDIO-SOLENOPSION LAURENTIAE)**						
*Anagallis parviflora*	2	1	1	2	+	5
*Radiola linoides*	+	.	1	.	+	3
*Cicendia filiformis*	.	+	+	.	+	3
**Char. Ord. (ISOËTETALIA)**						
*Centaurium maritimum*	1	1	.	1	1	4
*Briza minor*	+	.	+	+	.	3
**Char. Class (ISOËTO-NANOJUNCETEA)**						
*Mentha pulegium*	1	2	1	+	1	5
*Juncus bufonius*	2	2	1	2	1	5
*Lythrum hyssopifolia*	1	1	+	1	2	5
*Polypogon subspathaceus*	.	1	1	1	2	4
*Gaudinia fragilis*	+	+	.	.	+	3
*Juncus capitatus*	.	+	+	.	.	2
**Other species**						
*Blackstonia acuminata*	.	.	+	+	+	3
*Senecio vulgaris*	.	+	+	.	1	3
*Valantia muralis*	+	.	.	+	.	2
*Parapholis incurva*	+	1	.	.	.	2

**Localities and dates of relevés.** Rel. 1–5: Isola Grande dello Stagnone (Marsala)—19 April 2010.

**Table A21:** *Solenopsio gasparrinii-Isoëtetum siculae* Brullo, Cambria, Ilardi & Minissale ass. nova.

Relevé number	1	2	3	4	5	6	7	8	9	10	11	12	13	14	15	16	17	18	19	20	21	Presences
Altitude (m)	205	205	205	205	205	205	80	80	80	80	80	80	80	80	80	80	80	80	80	80	80	
Surface (m^2^)	4	5	5	4	2	2	1	1	1	0.5	0.5	1	1	1	1	0.5	0.5	0.5	0.5	0.5	1	
Coverage (%)	100	100	100	100	100	100	50	60	60	80	60	90	40	70	70	95	90	85	85	80	80	
**Char. Association**																						
*Isoëtes sicula*	4	4	5	3	4	5	2	1	3	2	2	4	1	1	+	1	3	3	2	+	1	21
**Char. All. (CICENDIO-SOLENOPSION LAURENTIAE)**																						
*Solenopsis gasparrinii*	1	2	2	2	1	2	1	3	+	2	3	1	1	3	3	4	4	3	2	4	3	21
*Anagallis parviflora*	2	1	1	2	2	2	.	.	+	+	+	.	.	+	+	.	.	.	.	+	1	13
*Ophioglossum lusitanicum*	2	2	2	1	2	2	.	.	.	+	.	+	+	.	.	.	+	.	.	.	.	10
*Radiola linoides*	.	.	.	.	.	.	.	.	1	+	+	.	.	.	+	+	+	+	1	.	+	9
*Anagallis minima*	2	2	3	2	1	2	.	.	.	.	.	.	.	.	.	.	.	.	.	.	.	6
*Cicendia filiformis*	1	+	+	+	.	+	.	.	.	.	.	.	.	.	.	.	.	.	.	.	.	5
**Char. Ord. (ISOËTETALIA)**																						
*Romulea ramiflora*	2	3	2	1	2	2	+	1	+	1	+	+	+	+	.	+	.	+	.	1	.	17
*Isolepis cernua*	1	+	.	1	1	+	.	.	.	.	1	.	1	.	.	1	2	2	3	.	3	12
*Centaurium maritimum*	.	.	.	.	.	.	+	+	.	.	+	1	1	+	+	+	.	.	.	+	.	9
*Lotus parviflorus*	1	+	1	1	2	1	.	.	.	.	.	.	.	.	.	.	.	.	.	.	.	6
*Bulliarda vaillantii*	.	.	.	.	.	.	.	.	2	1	.	.	.	+	.	.	.	.	.	.	.	3
*Triglochin laxiflora*	.	.	.	+	2	1	.	.	.	.	.	.	.	.	.	.	.	.	.	.	.	3
*Lotus angustissimus*	.	.	.	.	.	.	.	+	+	.	.	.	.	.	.	.	.	.	.	.	.	2
*Briza minor*	.	.	.	.	.	.	.	.	.	.	.	.	.	+	.	+	.	.	.	.	.	2
**Char. Class (ISOËTO-NANOJUNCETEA)**																						
*Polypogon subspathaceus*	3	2	3	2	3	2	1	+	1	+	1	.	1	1	2	2	2	2	1	2	2	20
*Juncus bufonius*	1	2	1	2	1	2	1	1	1	1	1	+	2	1	.	2	1	1	.	1	1	19
*Mentha pulegium*	2	1	1	3	2	3	1	+	+	1	+	.	1	.	2	.	+	+	+	.	1	17
*Lythrum hyssopifolia*	.	.	.	.	.	.	2	1	1	2	1	+	1	1	2	2	.	2	2	2	2	14
*Gaudinia fragilis*	.	.	.	.	.	.	2	1	1	3	1	+	2	1	+	.	.	1	+	1	2	13
*Juncus pygmaeus*	.	.	.	.	.	.	+	+	.	+	.	.	+	1	.	.	1	.	2	.	.	7
**Other species**																						
*Linum bienne*	2	2	+	1	1	1	+	1	.	1	1	.	1	.	1	1	1	+	+	.	1	17
*Centaurium tenuiflorum*	.	.	.	.	.	.	2	1	1	1	2	1	2	1	1	+	1	1	+	1	1	15
*Coleostephus myconis*	1	2	2	2	2	2	.	+	.	.	+	.	+	.	.	+	.	+	+	.	+	13
*Blackstonia perfoliate*	.	.	.	.	.	.	+	+	+	+	+	+	+	1	+	.	.	+	.	+	+	12
*Trifolium squarrosum*	.	.	.	.	.	.	1	2	1	2	1	1	.	.	+	.	.	.	.	.	.	7
*Carex divisa*	3	2	2	1	2	1	.	.	.	.	.	.	.	.	.	.	.	.	.	.	.	6
*Euphorbia akenocarpa*	+	1	+	+	1	1	.	.	.	.	.	.	.	.	.	.	.	.	.	.	.	6
*Bellis annua*	2	1	2	1	1	2	.	.	.	.	.	.	.	.	.	.	.	.	.	.	.	6
*Serapias lingua*	1	1	1	+	1	1	.	.	.	.	.	.	.	.	.	.	.	.	.	.	.	6
*Trifolium grandiflorum*	.	.	.	.	.	.	2	1	+	.	+	.	2	1	.	.	.	.	.	.	.	6
*Ranunculus paludosus*	2	1	1	1	1	1	.	.	.	.	.	.	.	.	.	.	.	.	.	.	.	6
*Trifolium resupinatum*	.	.	.	.	.	.	.	.	.	.	.	.	.	.	1	.	2	2	+	1	2	6
*Briza maxima*	.	.	.	.	.	.	.	+	.	+	+	.	1	.	.	.	.	.	.	.	+	5
*Anagallis arvensis*	.	.	.	.	.	.	.	.	.	.	.	.	.	+	.	+	.	.	.	+	+	4
*Veronica anagalloides*	.	.	.	.	.	.	.	.	.	.	.	.	.	.	.	.	+	+	1	.	.	3
*Logfia gallica*	.	.	.	.	.	.	+	.	.	1	2	.	.	.	.	.	.	.	.	.	.	3
*Aira cupaniana*	.	.	.	+	+	1	.	.	.	.	.	.	.	.	.	.	.	.	.	.	.	3
*Trifolium spumosum*	.	.	.	.	.	.	.	.	.	.	.	.	1	+	1	.	.	.	.	.	.	3
*Trifolium subterraneum*	.	1	1	.	.	1	.	.	.	.	.	.	.	.	.	.	.	.	.	.	.	3
*Parapholis incurve*	.	.	.	.	.	.	.	.	.	.	.	.	.	.	+	.	.	.	.	.	+	2
*Trifolium lappaceum*	.	.	.	.	.	.	.	.	.	.	.	.	.	.	.	+	+	.	.	.	.	2
*Lotus tetragonolobus*	.	.	.	.	.	.	.	.	.	.	.	.	.	.	.	.	.	.	.	+	.	1

**Localities and dates of relevés**. Rel. 1–6: Contrada Anguillara (Calatafimi)—28 April 2017; Rel. 7–15: Mazara del Vallo (Poggiallegro)—17 June 1978; rel. 16–21: Mazara del Vallo (Poggiallegro)—3 May 1979.

**Table A22:** Myosotido congestae-Isoëtetum histricis Azzaro & Cambria ass. nov.

Relevé number	1	2	3	Presences
Altitude (m)	323	323	323	
Surface (m^2^)	10	10	10	
Coverage (%)	70	80	70	
**Char. Association**				
*Isoetes histrix*	3	4	1	3
*Myosotis congesta*	+	+	+	3
*Aphanes arvensis*	1	+	+	3
**Char. Subassociation**				
*Callitriche brutia*	.	.	3	1
**Char. All. (CICENDIO-SOLENOPSION LAURENTIAE)**				
*Anagallis parviflora*	+	+	+	3
**Char. Ord. (ISOËTETALIA) and Class (ISOËTO-NANOJUNCETEA)**				
*Mentha pulegium*	1	+	1	3
*Poa infirma*	+	+	+	3
*Juncus bufonius*	2	2	1	3
*Juncus capitatus*	.	+	.	1
*Lotus angustissimus*	+	.	.	1
**Other species**				
*Tillaea alata*	1	+	+	3
*Sagina apetala*	1	1	+	3
*Trifolium suffocatum*	+	1	+	3
*Aira cupaniana*	+	+	.	2
*Ranunculus paludosus*	+	+	.	2
*Veronica arvensis*	+	+	.	2
*Glebionis segetum*	.	+	+	2
*Cerastium glomeratum*	.	.	+	1
*Filago pyramidata*	.	+	.	1
*Galium murale*	+	.	.	1
*Plantago bellardii*	.	+	.	1
*Valerianella eriocarpa*	+	.	.	1

**Localities and dates of relevés.** Rel. 1–3: Bosco di Santo Pietro (Caltagirone)—8 April 2021.

**Table A23:** Trifolio micheliani-Agrostidetum pourretii Cambria & Brullo ass. nov.

Relevé number	1	2	3	4	5	6	Presences
Altitude (m)	529	529	664	664	664	664	
Surface (m^2^)	10	10	10	10	20	20	
Coverage (%)	60	60	80	90	90	80	
**Char. Association**							
*Trifolium michelianum*	3	3	2	3	1	1	6
**Char. All. (AGROSTION SALMANTICAE )**							
*Agrostis pourretii*	+	1	3	2	4	4	6
**Char. Ord. (ISOËTETALIA)**							
*Ranunculus ophioglossifolius*	1	+	+	.	+	+	5
**Char. Class (ISOËTO-NANOJUNCETEA)**							
*Mentha pulegium*	2	2	1	+	.	+	5
*Juncus bufonius*	+	+	+	+	.	.	4
*Poa infirma*	.	+	+	.	+	.	3
*Ranunculus angulatus*	.	.	1	+	+	.	3
**Other species**							
*Lythrum junceum*	1	+	+	.	.	+	4
*Trifolium resupinatum*	+	+	.	.	.	.	2
*Callitriche stagnalis*	.	.	.	.	+	+	2
*Trifolium nigrescens*	.	.	.	+	.	.	1
*Rumex pulcher*	.	+	.	.	.	.	1

**Localities and dates of relevés.** Rel. 1–2: Margiazzo di Vallone Arcera (Ficuzza)—10 June 2020; rel. 3–6: Gorgo di Gaetanello (Ficuzza)—13 June 2020.

**Table A24:** *Plantagini intermediae-Cyperetum fusci* Sciandrello, D’Agostino & Minissale 2013.

Relevé number	1	2	3	4	5	6	7	8	9	10	11	12	13	14	15	16	17	18	19	20	21	22	23	24	25	26	27	28	29	Presences
Altitude (m)	679	678	678	679	677	662	654	654	620	620	620	630	620	620	620	589	620	615	615	615	615	615	615	615	615	615	615	615	615	
Surface (m^2^)	1	1	1	1	1	1	1	1	5	5	1	1	1	1	1	1	1	2	1	2	2	2	2	2	2	2	2	2	2	
Coverage (%)	90	85	90	90	90	85	90	95	90	80	80	90	85	80	85	80	85	85	85	90	85	90	100	100	100	85	90	90	100	
**Char. Association and All. (NANOCYPERION FLAVESCENTIS)**																														
*Cyperus flavescens*	.	.	.	.	.	.	.	.	1	+	+	2	3	1	2	3	3	4	3	3	3	4	4	4	5	4	3	4	4	21
*Plantago intermedia*	1	2	2	1	+	3	.	.	.	.	.	.	.	.	.	.	+	+	2	1	1	1	+	+	+	+	1	1	+	19
*Samolus valerandi*	.	.	.	.	.	+	+	.	.	.	.	.	1	+	1	2	1	1	+	1	+	1	2	3	1	1	1	1	2	19
**Char. Ord. (NANOCYPERETALIA)**																														
*Cyperus fuscus*	3	2	2	2	4	3	3	2	2	2	1	3	2	3	3	1	2	2	1	2	3	1	1	+	+	2	2	1	1	29
*Isolepis cernua*	.	.	.	.	.	.	.	.	.	.	.	.	.	.	.	.	.	.	.	+	.	+	+	.	+	.	+	+	+	7
*Euphorbia chamaesyce*	.	.	.	.	.	.	.	.	.	.	.	.	.	.	.	.	.	+	1	+	+		.	.	.	+	+	.	.	6
*Laphangium luteoalbum*	.	.	.	.	.	.	.	.	.	.	.		+	+	+	.	+	.	.	.	.	.	.	.	.	.	.	.	.	4
**Char. Class (ISOËTO-NANOJUNCETEA)**																														
*Mentha pulegium*	3	2	3	1	1	+	2	4	.	.	.	.	+	+	.	.	+	1	2	1	1	1	1	+	2	1	1	1	1	23
*Juncus hybridus*	1	+	1	+	.	2	.	.	.	.	.	.	.	.	.	.	.	.	+	+	+	.	.	.	.	+	+	+	.	11
*Lythrum hyssopifolia*	2	3	2	2	2	.	+	.	+	+	.	+	.	.	.	.	+	.	.	.	.	.	.	.	.	.	.	.	.	10
*Juncus bufonius*	3	2	3	3	1	+	1	+	.	.	.	.	.	.	.	.	,	.	.	.	.	.	.	.	.	,	,	,	,	8
*Lotus angustissimus*	.	.	.	.	.	.	+	+	.	.	.	.	.	.	.	.	.	.	.	.	.	.	.	.	.	.	.	.	.	2
**Other species**																														
*Helosciadium nodiflorum*	.	.	.	.	.	.	.	.	2	1	+	+	+	1	+	1	+	1	+	+	+	1	+	.	.	1	+	1	+	19
*Veronica anagallis-aquatica*	.	.	.	.	.	.	.	.	3	2	2	+	+	.	1	.	+	+	+	1	+	+	+	.	.	+	1	+	+	17
*Mentha suaveolens*	.	.	.	.	.	.	.	.	+	1	+	1	.	+	+	+	+	+	.	+	.	+	+	.	.	+	+	+	+	16
*Digitaria ciliaris*	.	.	.	.	.	.	.	.	.	.	.	.	+	+	+	.	+	1	1	1	1	1	+	.	.	1	1	1	+	14
*Juncus articulatus*	+	1	.	.	+	+	1	+	.	.	.	.	.	.	.	.	.	.	+	.	+	.	1	+	+	.	.	.	1	12
*Trifolium* sp.	.	.	.	.	.	.	.	.	.	.	.	.	1	+	1	+	.	+	1	1	+	+	1	1	+	.	.	.	.	12
*Rumex pulcher*	.	.	.	.	.	.	.	.	.	.	.	+	+	+	+	+	+	+	+	+	.	.	.	.	.	+	+	.	.	11
*Trifolium resupinatum*	.	.	.	.	.	+	+	+	.	.	.	.	.	.	.	.	1	.	.	.	.	.	.	.	.	+	1	+	1	8
*Polypogon viridis*	1	1	2	1	1	1	+	+	.	.	.	.	.	.	.	.	.	.	.	.	.	.	.	.	.	.	.	.	.	8
*Polygonum arenastrum*	.	.	.	.	.	.	.	.	.	.	.	.	.	.	.	.	.	+	+	+	+	+	.	.	.	+	+	+	.	8
*Polypogon maritimus*	+	+	+	+	+	+	.	.	.	.	.	.	.	.	.	.	.	.	.	.	.	.	.	.	.	.	.	.	.	6
*Persicaria laphatifolia*	.	.	.	.	.	.	.	.	.	.	.	.	.	.	.	.	.	+	+	.	+	+	.	.	.	+	.	+	.	6
*Nasturtium officinale*	.	.	.	.	.	.	.	.	+	1	3	.	+	.	+	.	+	.	.	.	.	.	.	.	.	.	.	.	.	6
*Verbena officinalis*	.	.	.	.	.	.	.	.	.	.	.	.	.	.	.	.	.	.	+	+	+	.	.	.	.	.	+	.	.	4
*Agrostis* sp.	.	.	.	.	.	.	.	.	.	.	.	.	.	.	.	.	.	.	.	+	+	+	.	.	.	.	.	.	.	3
*Callitriche stagnalis*	.	.	.	.	.	.	.	.	+	+	1	.	.	.	.	.	.	.	.	.	.	.	.	.	.	.	.	.	.	3
*Chara* sp.	.	.	.	.	.	.	.	.	+	.	+	.	.	.	.	.	.	.	.	.	.	.	.	.	.	.	.	.	.	2
*Epilobium parviflorum*	+	.	.	.	.	.	.	.	.	.	.	.	.	.	.	.	.	.	.	.	.	.	.	.	.	.	.	.	.	1
*Hypericum hircinum* subsp. *majus*	.	.	.	+	.	.	.	.	.	.	.	.	.	.	.	.	.	.	.	.	.	.	.	.	.	.	.	.	.	1
*Spergularia* sp.	.	.	.	.	.	.	.	.	.	.	.	.	.	.	.	.	.	+	.	.	.	.	.	.	.	.	.	.	.	1

**Localities and dates of relevés.** Rel. 1–8: Castelmola, Sciandrello et al. [140]—Table 7; rel. 9–12: Torrente Vacco (Fiumedinisi)—7 November 2019; rel. 13–17: Torrente Vacco (Fiumedinisi)—16 November 2019; rel. 18–29: Gole Santissima (Fiumedinisi)—4 October 2020.

**Table A25:** Gnaphalio luteoalbi-Verbenetum supinae Rivas Goday 1970.

Relevé number	1	2	Presences
Altitude (m)	140	140	
Surface (m^2^)	10	10	
Coverage (%)	70	100	
**Char. Association**			
*Laphangium luteoalbum*	2	2	2
**Char. All. (VERBENION SUPINAE) and** **Ord. (NANOCYPERETALIA)**			
*Verbena supina*	3	1	2
*Paspalum distichum*	2	1	2
*Schenkia spicata*	1	1	2
*Sporobolus schoenoides*	1	.	1
**Char. Class (ISOËTO-NANOJUNCETEA)**			
*Juncus hybridus*	+	1	2
**Other species**			
*Xanthium italicum*	1	2	2
*Symphyotrichum squamatum*	+	1	2
*Medicago intertexta*	3	3	2
*Sonchus oleraceus*	+	+	2
*Phragmites australis*	+	.	1
*Polypogon monspeliensis*	.	+	1
*Polygonum aviculare*	1	.	1
*Rumex pulcher*	.	1	1
*Medicago polymorpha*	.	+	1

**Localities and dates of relevés.** Rel. 1–2: Cimia dam, Sciandrello [91]—Tab. 3 rel. 16–17.

**Table A26:** Heliotropio supini-Heleochloetum schoenoidis Rivas Goday 1956.

Relevé number	1	2	3	4	5	6	7	8	9	10	11	12	13	14	15	16	17	18	19	20	21	22	23	24	25	26	27	28	29	Presences
Altitude (m)	50	366	50	50	680	680	680	835	835	680	640	640	640	835	680	680	680	680	950	950	835	90	90	90	90	90	90	140	140	
Surface (m^2^)	100	100	100	100	100	100	100	100	100	100	100	100	100	100	100	100	100	100	100	100	100	10	5	10	30	20	25	20	10	
Coverage (%)	20	40	50	20	20	30	40	40	50	50	80	70	80	60	90	90	50	60	40	60	70	60	70	70	90	85	80	80	90	
**Char. Association**																														
*Heliotropium supinum*	+	+	1	1	1	1	1	2	3	3	4	4	4	4	5	1	1	2	1	2	4	1	2	4	5	4	3	4	4	29
*Sporobolus schoenoides*	.	1	+	.	+	+	+	.	.	.	.	.	.	.	.	+	1	.	+	+	.	2	3	2	1	3	4	+	+	18
**Char. All. (VERBENION SUPINAE)**																														
*Euphorbia chamaesyce*	.	1	1	+	2	2	2	1	.	1	1	1	2	.	1	1	1	2	.	.	.	.	.	.	.	.	.	3	+	17
*Paspalum distichum*	+	+	3	+	+	1	.	2	+	.	.	.	.	.	.	1	1	+	.	.	.	+	+	.	.	+	+	1	1	17
*Pulicaria sicula*	.	.	+	.	.	.	+	.	.	+	.	.	.	.	+	1	1	.	.	.	.	.	.	.	.	.	.	.	.	6
*Coronopus squamatus*	.	.	.	.	.	+	1	.	.	.	+	1	.	.	.	.	.	.	.	.	2	.	.	.	.	.	.	.	.	5
*Verbena supina*	+	.	.	.	.	.	.	.	.	.	.	.	.	.	.	.	.	.	.	.	.	.	.	.	.	.	.	3	1	3
*Sporobolus aculeatus*	1	.	.	1	.	.	.	.	.	.	.	.	.	.	.	.	.	.	.	.	.	.	.	.	.	.	.	.	.	2
*Schenkia spicata*	.	.	.	.	.	.	.	.	.	.	.	.	.	.	.	.	.	.	.	.	.	.	.	.	+	.	.	.	.	1
**Char. Ord. (NANOCYPERETALIA)**																														
*Cyperus fuscus*	.	.	.	.	.	.	+	.	.	.	.	.	.	.	+	1	1	.	.	.	.	.	.	.	.	.	.	.	.	4
*Plantago intermedia*	.	.	+	.	.	.	+	.	.	.	.	+	.	.	.	.	.	.	.	.	+	.	.	.	.	.	.	.	.	4
**Char. Class (ISOËTO-NANOJUNCETEA)**																														
*Mentha pulegium*	+	+	+	+	+	+	+	+	+	+	+	+	+	+	+	1	1	+	.	.	3	.	.	.	.	.	.	.	.	19
*Lythrum hyssopifolia*	+	.	1	+	+	+	1	1	+	.	+	.	.	.	.	.	.	.	.	.	.	.	.	.	.	.	.	.	.	9
*Mentha pulegium*	.	.	.	.	+	.	.	.	.	.	.	.	.	.	.	.	.	.	+	1	.	.	.	.	.	.	.	.	.	3
**Other species**																														
*Xanthium spinosum*	+	+	.	+	+	.	2	.	+	1	+	2	+	+	1	5	3	3	+	+	1	.	.	.	.	.	.	.	.	18
*Helminthotheca echioides*	+	+	.	+	+	1	+	+	+	.	+	+	+	+	+	+	.	+	+	.	+	.	.	.	.	.	.	.	.	17
*Convolvulus arvensis*	+	+	.	.	.	+	+	.	+	.	+	+	1	2	.	+	+	+	+	.	1	.	.	.	.	.	.	.	.	14
*Verbena officinalis*	+	+	+	+	.	1	1	+	+	.	.	.	.	.	+	+	+	+	.	.	.	.	.	.	.	.	.	.	.	12
*Xanthium italicum*	.	.	2	+	.	.	.	1	.	+	.	.	.	.	+	+	+	+	.	2	.	.	.	+	1	+	.	.	.	12
*Trifolium fragiferum*	+	.	+	+	+	+	+	.	.	+	.	.	.	.	.	1	+	1	.	.	.	.	.	.	.	.	.	.	.	10
*Solanum nigrum*	+	.	1	+	+	.	+	1	.	+	+	.	.	.	.	.	.	.	1	1	.	.	.	.	.	.	.	.	.	10
*Cynodon dactylon*	2	3	+	1	.	.	+	+	+	.	.	.	.	.	.	.	.	.	.	+	+	.	.	.	.	.	.	.	.	9
*Kickxia spuria* subsp. i*ntegrifolia*	.	+	.	.	.	.	+	.	1	+	.	.	.	+	.	+	.	.	+	.	+	.	.	.	.	.	.	.	.	8
*Tamarix gallica*	.	.	.	.	.	.	.	.	.	.	.	.	.	.	.	.	.	.	.	.	.	+	+	+	+	+	+	+	.	7
*Symphyotrichum squamatum*	.	.	.	.	.	.	.	.	.	.	.	.	.	.	.	.	.	.	.	.	.	.	.	2	2	2	1	+	+	6
*Atriplex prostrata*	.	.	.	.	.	.	.	.	.	.	.	.	.	.	.	.	.	.	.	.	.	2	1	2	1	+	1	.	.	6
*Rumex pulcher*	.	.	.	.	.	.	.	.	.	.	.	.	.	.	.	.	.	.	.	.	.	+	+	1	1	+	1	.	.	6
*Persicaria laphatifolia*	.	.	.	.	.	.	.	.	.	.	.	.	.	.	.	.	.	.	.	.	.	2	1	.	1	+	+	.	.	5
*Phragmites australis*	.	.	.	.	.	.	.	.	.	.	.	.	.	.	.	.	.	.	.	.	.	+	+	+	.	+	+	.	.	5
*Polygonum aviculare*	.	.	.	.	.	.	.	.	.	.	.	.	.	.	.	.	.	.	.	.	.	.	.	1	+	1	1	.	.	4
*Polypogon monspeliensis*	.	.	.	.	.	.	.	.	.	.	.	.	.	.	.	.	.	.	.	.	.	.	.	+	1	+	+	.	.	4
*Sonchus oleraceus*	.	.	.	.	.	.	.	.	.	.	.	.	.	.	.	.	.	.	.	.	.	.	.	+	1	+	+	.	.	4
*Sulla coronaria*	.	.	.	.	.	.	.	.	.	.	.	.	.	.	.	.	.	.	.	.	.	.	.	+	+	+	+	.	.	4
*Dysphania ambrosioides*	.	.	.	.	.	.	.	.	.	.	.	.	.	.	.	.	.	.	.	.	.	.	.	+	+	1	.	.	.	3
*Oxybasis rubra*	.	.	.	.	.	.	.	.	.	.	.	.	.	.	.	.	.	.	.	.	.	1	+	.	.	.	.	.	1	3
*Ecballium elaterium*	.	.	.	.	.	.	.	.	.	.	.	.	.	.	.	.	.	.	.	.	.	.	.	+	+	.	.	.	+	3
*Scolymus maculatus*	.	.	.	.	.	.	.	.	.	.	.	.	.	.	.	.	.	.	.	.	.	.	.	+	+	+	.	.	.	3
*Medicago doliata*	.	.	.	.	.	.	.	.	.	.	.	.	.	.	.	.	.	.	.	.	.	1	1	.	+	.	.	.	.	3
*Heliotropium curassavicum*	.	.	.	.	.	.	.	.	.	.	.	.	.	.	.	.	.	.	.	.	.	.	.	+	.	+	+	.	.	3
*Lythrum junceum*	.	.	.	.	.	.	.	.	.	.	.	.	.	.	.	.	.	.	.	.	.	+	+	.	+	.	.	.	.	3
*Chrozophora tinctoria*	.	.	.	.	.	.	.	.	.	.	.	.	.	.	.	.	.	.	.	.	.	.	.	.	.	.	.	+	+	2
*Medicago polymorpha*	.	.	.	.	.	.	.	.	.	.	.	.	.	.	.	.	.	.	.	.	.	.	.	.	+	.	.	.	.	1

**Localities and dates of relevés.** Rel. 1–21: Brullo & Marcenò [79]—Tab 1 rel. 1–8, 10–13, 15–17, 21–23, 25–26, 33; rel. 22–29: Comunelli and Cimia dams (Butera), Sciandrello [91]—Tab 3 rel. 8–15.

**Table A27:** Glino lotoidis-Verbenetum supinae Rivas Goday 1964.

Relevé number	1	2	3	4	5	6	7	8	9	10	11	12	13	14	15	16	17	18	19	20	21	22	23	24	25	26	27	28	29	30	31	32	33	34	Presences
Altitude (m)	195	195	195	195	195	195	195	195	195	520	520	520	520	160	160	610	610	610	610	610	610	610	610	610	610	610	610	160	160	160	160	160	160	160	
Surface (m^2^)	100	100	100	100	100	100	100	100	100	100	100	100	100	100	100	200	100	100	100	100	100	50	200	100	100	100	200	50	50	80	50	30	40	20	
Coverage (%)	80	80	90	80	80	90	60	50	60	20	15	10	25	70	70	50	70	70	50	50	70	70	15	40	70	80	80	70	70	80	80	90	80	80	
**Char. Association**																																			
*Glinus lotoides*	4	4	5	4	4	5	3	1	3	2	1	1	2	1	4	1	2	2	2	2	2	3	1	1	3	4	3	1	2	1	1	1	1	4	34
**Char. All. (VERBENION SUPINAE)**																																			
*Paspalum distichum*	+	.	1	1	+	+	+	+	.	1	+	2	.	1	2	2	3	3	2	2	+	1	+	2	2	2	1	2	1	2	2	1	1	+	31
*Sporobolus schoenoides*	2	.	2	1	2	1	1	1	+	+	+	+	.	.	.	2	2	2	1	2	2	3	+	+	2	1	1	2	3	2	4	4	3	2	30
*Heliotropium supinum*	2	3	2	2	3	2	2	3	2	1	1	+	+	4	1	3	1	+	+	2	.	+	1	+	1	+	+	.	.	+	+	+	.	.	29
*Verbena supina*	2	.	+	1	2	2	+	.	.	+	.	+	+	1	+	2	1	+	+	2	1	2	1	1	2	.	+	3	3	4	3	2	+	1	29
*Euphorbia chamaesyce*	.	.	.	+	.	+	+	.	.	.	.	.	.	+	+	1	+	.	.	+	+	.	+	+	+	.	+	+	1	+	+	+	+	+	20
*Coronopus squamatus*	.	.	.	.	.	.	.	.	.	+	+	2	.	.	.	.	.	.	.	.	.	.	.	+	1	.	.	.	.	.	.	.	.	.	5
*Pulicaria sicula*	.	.	.	.	.	.	.	.	.	.	.	.	.	.	+	.	.	.	.	.	.	.	+	.	.	.	.	.	.	.	.	.	.	.	2
*Sporobolus alopecuroides*	.	.	.	.	.	.	.	.	.	.	.	.	.	.	.	.	.	.	.	.	.	.	.	.	.	.	.	.	.	.	.	.	.	1	1
**Char. Ord. (NANOCYPERETALIA)**																																			
*Plantago intermedia*	1	.	+	+	.	+	+	.	.	+	+	+	+	.	.	+	+	+	+	1	.	1	+	1	1	1	1	.	.	.	.	.	.	.	20
*Corrigiola litoralis*	.	.	1	1	+	+	+	.	.	.	1	.	+	.	.	+	.	.	+	.	+	+	+	+	+	+	.	.	.	.	.	.	.	.	15
*Gnaphalium uliginosum* var. *prostratum*	.	.	.	.	.	.	.	.	.	.	.	.	.	.	.	.	1	1	+	1	3	2	+	+	+	+	1								11
*Cyperus fuscus*	.	.	2	1	+	.	1	.	+	.	.	.	.	.	.	.	.	.	.	.	.	.	.	.	.	+	.	.	.	.	.	.	1	.	7
*Cyperus michelianus*	.	.	.	.	.	.	.	.	.	.	.	.	.	.	.	.	.	.	.	.	.	+	.	.	+	+	+	.	.	.	.	.	.	.	4
**Char. Class (ISOËTO-NANOJUNCETEA)**																																			
*Lythrum hyssopifolia*	+	.	.	.	.	+	.	.	.	+	+	+	+	.	.	.	.	.	.	.	+	.	.	1	.	+	+	.	.	.	.	.	.	.	10
*Mentha pulegium*	+	.	.	.	.	.	.	.	.	.	+	+	.	.	.	.	.	.	.	.	.	.	.	+	+	.	.	+	+	1	+	.	.	.	9
*Eryngium pusillum*	.	.	.	.	.	.	.	.	.	.	.	.	.	.	.	2	.	.	.	+	.	.	.	+	2	.	+	.	.	.	.	.	.	.	5
*Juncus hybridus*	.	.	.	.	.	.	.	.	.	.	.	.	.	.	.	.	.	.	.	.	.	.	.	.	.	.	.	+	.	.	.	.	.	.	1
*Veronica anagalloides*	.	.	.	.	.	+	.	.	.	.	.	.	.	.	.	.	.	.	.	.	.	.	.	.	.	.	.	.	.	.	.	.	.	.	1
**Other species**																																			
*Persicaria laphatifolia*	+	1	+	+	+	.	.	.	+	+	.	+	+	.	.	.	+	1	1	.	+	+	+	.	+	+	+	.	.	.	.	+	2	+	22
*Xanthium spinosum*	.	.	+	+	+	.	+	+	.	1	+	+	+	.	.	+	1	+	.	+	+	+	+	+	2	2	+	.	.	.	.	.	.	.	20
*Trifolium fragiferum*	+	+	+	+	.	+	.	+	.	1	+	+	+	.	.	.	+	+	+	+	+	+	.	.	+	+	+	.	.	.	.	.	.	.	19
*Amaranthus deflexus*	.	.	+	+	+	.	.	.	.	1	1	.	1	.	.	.	+	+	.	.	.	+	.	.	+	+	+	+	+	+	+	.	.	.	16
*Xanthium italicum*	+	.	.	.	+	+	.	.	.	.	.	.	+	+	+	.	.	.	.	.	.	.	.	.	.	.	.	1	1	+	+	2	+	+	13
*Helminthotheca echioides*	+	+	.	+	.	.	.	.	.	+	.	.	+	+	.	.	.	.	.	.	+	.	.	.	.	.	.	+	1	2	1	+	+	.	13
*Oxybasis rubra*	.	.	.	.	.	+	.	.	.	.	.	.	+	.	.	.	.	+	.	+	1	.	.	+	+	+	1	.	.	.	.	.	+	.	10
*Rumex pulcher*	.	.	.	.	.	.	.	.	.	+	+	+	.	.	.	.	.	.	.	.	.	.	+	.	.	.	.	+	.	+	+	1	+	.	10
*Atriplex prostrata*	+	.	.	.	.	.	.	.	.	.	.	.	.	.	.	.	.	+	+	+	.	+	.	.	.	.	.	1	1	1	1	.	.	+	10
*Symphyotrichum squamatum*	1	.	.	.	+	1	.	.	.	+	+	.	.	.	+	.	.	.	.	.	.	.	.	.	.	.	.	+	+	+	+	.	.	.	10
*Echinochloa crus-galli*	.	.	1	+	+	+	+	1	.	.	.	+	.	.	.	.	+	.	.	+	.	.	.	.	.	1	.	.	.	.	.	.	.	.	10
*Portulaca oleracea*	.	.	.	.	.	.	.	.	.	1	.	1	1	.	.	.	+	.	+	+	+	+	.	.	.	+	+	.	.	.	.	.	.	.	10
*Cyperus rotundus*	.	.	+	+	+	.	.	.	.	.	.	.	.	.	.	.	+	+	.	.	.	.	.	.	+	+	.	+	1	+	+	.	.	.	10
*Polygonum aviculare*	+	.	+	.	+	+	.	.	.	1	+	+	+	.	+	.	.	.	.	.	.	.	.	.	.	.	.	.	.	.	.	.	.	.	9
*Verbena officinalis*	+	.	.	.	.	2	.	+	+	.	.	.	.	.	.	.	.	.	.	.	.	.	.	.	.	.	.	+	+	+	+	.	.	.	8
*Malva sylvestris*	.	+	.	.	+	+	.	.	.	.	.	.	.	.	.	.	.	+	+	.	.	.	.	.	+	.	+	.	.	.	.	.	.	.	7
*Sonchus asper*	.	.	+	.	.	.	.	.	.	.	.	.	.	.	.	.	.	.	.	.	.	.	+	.	.	.	.	+	+	+	+	+	.	.	7
*Cynodon dactylon*	+	+	.	.	+	.	.	+	.	.	+	+	.	.	.	.	.	.	.	.	.	.	.	.	.	.	.	.	.	.	.	.	.	.	6
*Mentha suaveolens*	+	.	+	+	+	.	+	.	.	.	.	.	.	.	.	.	.	.	.	.	.	+	.	.	.	.	.	.	.	.	.	.	.	.	6
*Tamarix gallica*	.	.	.	.	.	.	.	.	.	.	.	.	.	.	.	.	.	.	.	.	.	.	.	.	.	.	.	+	1	1	+	+	+	.	6
*Kickxia spuria* subsp. *integrifolia*	.	.	.	.	.	.	.	.	.	+	.	1	.	.	.	+	.	.	.	.	.	+	.	.	.	+	+	.	.	.	.	.	.	.	6
*Chenopodium album*	.	.	.	.	.	.	.	.	.	+	.	.	.	.	+	+	.	.	.	.	.	.	.	.	.	+	+	.	.	.	.	.	.	.	5
*Potentilla reptans*	.	.	.	.	+	.	.	.	.	.	.	.	.	.	.	.	+	+	.	.	.	.	.	+	+	.	.	.	.	.	.	.	.	.	5
*Ajuga reptans*	.	.	.	.	.	.	.	.	.	1	+	+	1	.	.	.	.	.	.	.	.	.	.	.	.	.	.	.	.	.	.	.	.	+	5
*Beta maritima*	.	.	.	.	.	.	.	.	.	.	.	.	.	.	.	.	.	.	.	.	.	.	.	.	.	.	.	1	2	1	1	.	.	.	4
*Visnaga daucoides*	.	.	.	.	.	.	.	.	.	+	.	.	.	.	.	.	.	.	.	.	.	.	.	+	.	+	+								4
*Anagallis arvensis*	.	.	.	.	.	.	.	.	.	.	.	.	.	.	.	.	.	.	+	.	.	.	+	.	.	.	+	.	.	.	.	.	.	.	3
*Scolymus maculatus*	.	.	.	.	.	.	.	.	.	.	.	.	.	.	.	.	.	.	.	.	.	.	.	.	.	.	.	+	.	+	+	.	.	.	3
*Convolvulus arvensis*	.	.	.	.	.	.	.	.	+	.	.	.	.	.	.	.	+	.	+	.	.	.	.	.	.	.	.	.	.	.	.	.	.	.	3
*Lythrum junceum*	.	.	.	.	.	.	.	.	.	.	.	.	.	.	.	.	.	.	.	.	.	.	.	.	.	.	.	1	.	+	.	.	.	.	2
*Pulicaria dysenterica*	.	.	.	.	.	.	.	.	.	.	.	.	.	.	.	.	.	.	.	.	.	+	.	.	.	.	+	.	.	.	.	.	.	.	2
*Chrozophora tinctoria*	.	.	.	.	.	.	.	.	.	.	.	.	.	.	.	.	.	.	.	.	.	.	.	.	.	.	.	.	.	.	.	+	.	.	1
*Polypogon monspeliensis*	.	.	.	.	.	.	.	.	.	.	.	.	.	.	.	.	.	.	.	.	.	.	.	.	.	.	.	.	.	.	.	+	.	.	1

**Localities and dates of relevés.** Rel. 1–27: Brullo & Marcenò [79]—Table 1, rel. 36–62; rel. 28–34: Disueri dam (Gela), Sciandrello [91]—Table 3 rel. 1–7.

**Table A28:** Coronopo squamati-Sisymbrelletum dentatae Minissale & Spampinato 1987.

Relevé number	1	2	3	4	5	6	7	8	9	10	11	12	13	14	15	16	Presences
Altitude (m)	850	850	850	850	850	850	850	850	850	850	850	850	850	850	850	700	
Surface (m^2^)	40	50	50	30	50	6	2	2	10	30	50	50	50	2	20	10	
Coverage (%)	70	90	50	90	60	60	70	70	80	60	70	70	70	60	60	90	
**Char. Association**																	
*Sisymbrella dentata*	3	4	2	3	2	2	3	2	2	2	3	3	4	2	1	1	16
*Anthemis cotula*	2	.	2	3	1	2	2	+	1	1	2	2	1	.	.	2	13
**Char. All. (VERBENION SUPINAE) and Ord. (NANOCYPERETALIA)**																	
*Hordeum geniculatum*	.	2	1	+	+	2	1	2	.	2	1	+	.	.	1	3	12
*Coronopus squamatus*	.	.	.	.	.	1	2	2	2	3	2	3	2	1	2	2	11
*Ranunculus sardous*	.	.	.	.	.	.	.	.	+	+	1	1	+	.	.	2	6
*Teucrium campanulatum*	2	2	2	2	.	.	.	.	.	.	.	.	.	.	.	.	4
**Char. Class (ISOËTO-NANOJUNCETEA)**																	
*Eryngium pusillum*	4	2	1	2	5	+	2	3	3	1	1	2	+	1	3	1	16
*Mentha pulegium*	1	3	2	3	4	1	1	3	3	3	3	3	2	2	2	.	15
*Ranunculus trilobus*	+	+	+	+	2	.	.	.	.	.	.	.	.	.	.	.	5
*Gaudinia fragilis*	.	+	1	+	.	.	.	.	1	.	.	.	.	.	.	.	4
*Poa infirma*	.	.	.	.	.	.	.	.	.	.	+	1	+	.	.	+	4
*Lythrum hyssopifolia*	.	.	.	.	.	.	.	.	.	.	.	+	.	.	.	.	1
*Pulicaria vulgaris* var. *vulgaris*	.	.	.	.	.	.	.	.	.	.	.	.	.	.	.	1	1
**Other species**																	
*Convolvulus arvensis*	1	1	2	2	2	1	2	2	3	1	1	1	1	2	2	1	16
*Plantago lanceolata*	.	.	.	.	+	+	+	+	+	.	.	+	.	1	+	1	9
*Potentilla reptans*	+	2	1	+	1	.	.	.	+	.	.	.	.	.	+	1	8
*Rumex conglomerartus*	+	+	1	1	+	.	.	.	.	.	.	.	.	.	.	.	5
*Polygonum aviculare*	.	.	.	.	.	.	.	.	.	+	+	+	+	.	.	2	5
*Elymus repens*	.	+	.	.	.	.	.	.	.	2	2	1	.	1	.	.	5
*Xanthium italicum*	.	.	.	.	.	.	.	.	.	.	2	2	2	.	.	1	4
*Cichorium intybus*	.	.	.	.	.	.	1	1	+	1	.	.	.	.	.	.	4
*Helminthotheca echioides*	.	.	.	.	.	.	.	.	.	+	+	+	+	.	.	.	4
*Galium debile*	.	.	.	.	.	1	.	1	.	.	.	.	.	2	.	.	3
*Xanthium spinosum*	.	.	.	.	.	.	.	.	.	+	2	.	+	.	.	.	3
*Galium elongatum*	.	+	+	+	.	.	.	.	.	.	.	.	.	.	.	.	3
*Anagallis arvensis*	+	.	1	+	.	.	.	.	.	.	.	.	.	.	.	.	3
*Rostraria hispida*	.	1	.	.	.	1	.	.	.	.	.	.	.	.	.	.	2
*Beta maritima*	+	.	.	+	.	.	.	.	.	.	.	.	.	.	.	.	2
*Cirsium italicum*	.	.	.	.	.	.	.	.	.	1	.	.	+	.	.	.	2
*Echium pustulatum*	.	.	.	+	.	.	.	.	.	.	.	.	.	.	.	.	1
*Plantago major*	.	.	.	.	.	.	.	.	1	.	.	.	.	.	.	.	1

**Localities and dates of relevés.** Rel. 1–5: Gurrida lake (Etna)—4 July 2008; rel. 6–15: Gurrida lake (Etna), Minissale & Spampinato [87]—Table 8; rel. 16: Castiglione di Sicilia—11 June 1986.

**Table A29:** Heleochloo schoenidis-Chenopodietum botryoidis Brullo & Sciandrello 2006.

Relevé number	1	2	3	4	5	6	7	8	9	10	Presences
Altitude (m)	5	5	5	5	5	5	5	5	5	5	
Surface (m^2^)	50	50	10	20	20	100	100	100	50	50	
Coverage (%)	70	60	70	25	70	40	50	70	70	80	
**Char. Association**											
*Oxybasis chenopodioides*	4	3	3	1	2	3	3	4	2	2	10
**Char. All. (VERBENION SUPINAE ) and Ord. (NANOCYPERETALIA)**											
*Sporobolus schoenoides*	1	1	3	+	1	1	2	2	3	4	10
*Cyperus fuscus*	.	.	.	2	1	+	+	1	+	.	6
*Sporobolus aculeatus*	.	.	.	.	.	1	+	+	.	.	3
**Char. Class (ISOËTO-NANOJUNCETEA)**											
*Juncus hybridus*	.	.	.	.	2	.	.	1	1	+	4
**Other species**											
*Atriplex prostrata*	1	1	1	+	+	1	+	1	2	+	10
*Tamarix africana*	3	2	+	2	2	2	3	3	2	3	10
*Symphyotrichum squamatum*	+	+	+	.	+	1	+	1	2	+	9
*Sonchus asper*	1	+	+	.	+	1	1	2	2	+	9
*Persicaria lapathifolia*	2	2	2	+	+	.	1	+	.	+	8
*Juncus maritimus*	1	+	.	.	3	.	+	+	+	+	7
*Phragmites australis*	+	+	.	.	.	+	.	+	+	1	6
*Chenopodium album*	+	+	.	.	.	+	1	1	+	.	6
*Polypogon monspeliensis*	.	.	.	.	.	+	+	1	2	1	5
*Typha latifolia*	.	.	.	.	.	.	1	+	1	+	4
*Bolboschoenus maritimus*	.	.	.	.	.	1	1	1	+	.	4
*Medicago polymorpha*	.	.	.	.	.	+	+	.	+	+	4
*Dysphania ambrosioides*	.	.	.	.	.	+	+	1	+	.	4
*Conyza bonariensis*	+	.	.	.	.	.	+	+	+	.	4
*Phyla nodiflora*	.	.	.	.	.	1	+	1	.	.	3
*Cynodon dactylon*	.	.	.	.	.	.	1	+	+	.	3
*Polypogon viridis*	.	.	.	.	.	.	+	1	+	.	3
*Senecio vulgaris*	.	.	.	.	.	.	+	+	+	.	3
*Lythrum junceum*	.	.	.	.	.	.	+	1	+	.	3
*Dittrichia viscosa*	+	.	.	.	.	.	.	.	+	.	2
*Xanthium italicum*	.	.	.	.	.	.	.	1	+	.	2
*Helosciadium nodiflorum*	.	.	.	.	.	.	+	+	.	.	2
*Schoenoplectus tabernaemontani*	.	.	.	.	.	.	1	+	.	.	2
*Rumex conglomeratus*	.	.	.	.	.	.	+	+	.	.	2
*Cyperus distachyos*	.	.	.	.	.	.	+	+	.	.	2
*Convolvulus arvensis*	.	.	.	.	.	.	.	+	+	.	2
*Tussilago farfara*	+	+	.	.	.	.	.	.	.	.	2
*Convolvulus silvaticus*	.	.	.	.	.	.	.	+	.	.	1
*Scirpoides holoschoenus*	.	.	.	.	.	+	.	.	.	.	1
*Samolus valerandi*	.	.	.	.	.	.	+	.	.	.	1
*Lotus preslii*	+	.	.	.	.	.	.	.	.	.	1
*Persicaria maculosa*	.	.	.	.	.	.	.	+	.	.	1
*Trigonella sulcata*	.	.	.	.	.	.	.	+	.	.	1
*Hypochoeris achyrophorus*	.	.	.	.	.	.	.	+	.	.	1
*Coronilla repanda*	.	.	.	.	.	.	.	+	.	.	1
*Spergularia marina*	.	.	.	.	.	.	.	+	.	.	1

**Localities and dates of relevés.** Rel. 1–10: Biviere di Gela, Brullo & Sciandrello [89]—Table 2.

**Table A30:** Coronopo squamati-Corrigioletum litoralis Brullo & C. Brullo ass. nov.

Relevé number	1	2	3	4	5	Presences
Altitude (dam)	120	120	120	120	120	
Surface (m^2^)	2	1	3	2	2	
Coverage (%)	60	60	50	30	30	
**Char. Association**						
*Corrigiola litoralis*	2	3	2	1	1	5
**Char. All. (VERBENION SUPINAE ) and Ord. (NANOCYPERETALIA)**						
*Coronopus squamatus*	+	+	1	1	+	5
*Spergularia rubra*	2	+	1	1	+	5
*Laphangium luteoalbum*	.	.	+	+	.	2
**Char. Class (ISOËTO-NANOJUNCETEA)**						
*Mentha pulegium*	2	2	3	2	1	5
*Juncus bufonius*	1	2	+	1	+	5
*Lythrum hyssopifolia*	+	.	+	+	1	4
*Polypogon subspathaceus*	+	+	.	+	+	4
**Other species**						
*Polygonum aviculare*	1	1	2	+	1	5
*Polypogon viridis*	2	1	1	.	+	4
*Lolium perenne*	1	.	1	+	+	4
*Plantago major*	+	1	.	.	+	3

**Localities and dates of relevés.** Rel. 1–5: Argimusco (Bosco di Malabotta)—29 July 1992.

**Table A31:** *Damasonio bourgaei-Crypsietum aculeatae* Rivas-Martínez & Costa in Rivas-Martínez et al. 1980.

Relevé number	1	2	3	4	5	6	7	8	9	10	11	12	13	14	15	16	17	18	19	20	21	22	23	24	25	26	27	28	29	30	31	Presences
Altitude (m)	9	9	9	9	9	9	9	9	9	9	9	9	9	9	9	9	9	9	9	9	9	9	9	10	10	10	6	6	6	8	8	
Surface (m^2^)	40	50	50	30	50	40	20	30	20	20	10	20	30	30	3	5	5	20	5	10	10	5	10	30	20	10	10	10	10	10	10	
Coverage (%)	70	90	50	90	60	70	90	70	40	90	90	70	60	40	80	80	90	60	70	80	90	70	90	100	100	100	100	100	100	90	100	
**Char. Association**																																
*Cressa cretica*	2	1	+	2	3	2	3	1	1	1	2	1	1	+	2	2	2	3	3	1	1	1	2	2	3	3	+	+	+	1	+	31
*Damasonium bourgaei*	1	3	1	2	1	1	+	+	+	4	3	2	3	3	+	+	1	2	3	+	4	.	4	3	1	2	4	5	4	1	+	31
**Char. All. (LYTHRION TRIBRACTEATI ) and Ord. (NANOCYPERETALIA)**																																
*Pulicaria sicula*	+	.	.	.	.	.	.	1	+	.	+	.	.	1	+	2	2	2	3	2	2	2	3	1	1	1	+	+	+	2	1	22
*Sporobolus aculeatus*	3	4	2	4	1	2	2	1	2	2	3	2	+	.	+	1	.	2	1	1	.	+	.	.	.	.	.	.	.	.	.	19
*Hordeum marinum*	.	.	.	.	.	+	+	.	1	.	.	.	.	+	+	1	+	1	+	+	+	1	+	.	.	.	.	+	.	+	+	16
*Heliotropium supinium*	3	+	3	+	2	1	+	1	1	1	2	3	2	.	.	.	.	.	.	.	.	.	1	.	.	.	.	.	+	.	.	15
*Lythrum tribracteatum*	.	.	.	.	.	.	.	.	.	.	.	.	.	.	.	.	.	1	+	3	4	3	5	5	5	5	5	3	3	4	5	14
*Schenkia spicata*	.	.	.	.	.	.	.	.	+	.	.	.	.	+	1	+	.	1	+	2	+	1	+	.	.	.	.	+	+	1	1	12
*Paspalum distichum*	2	+	+	2	+	1	2	2	.	.	.	.	.	.	.	.	.	.	.	.	.	.	.	.	.	.	.	.	.	.	.	8
*Euphorbia chamaesyce*	+	.	+	.	+	.	.	.	.	1	+	+	.	+	.	.	.	.	.	.	.	.	.	.	.	.	.	.	.	.	.	7
*Coronopus squamatus*	.	.	.	.	.	.	.	.	.	.	.	.	.	.	+	1	.	.	.	.	.	.	.	.	.	.	.	.	.	.	.	2
**Char. Class (ISOËTO-NANOJUNCETEA)**																																
*Mentha pulegium*	+	+	.	.	.	+	+	1	.	1	1	+	+	1	.	.	.	.	.	.	3	.	3	.	.	.	2	1	1	.	.	15
*Lythrum hyssopifolia*	.	.	.	.	.	.	.	.	.	3	3	1	3	.	.	+	.	.	.	.	2	3	.	.	.	.	.	.	.	1	2	9
*Juncus hybridus*	.	.	.	.	.	.	.	.	.	.	.	.	.	.	.	.	.	1	+	1	+	1	1	.	.	.	.	.	.	.	.	6
*Gaudinia fragilis*	.	.	.	.	.	.	.	.	.	.	.	.	.	.	.	.	.	.	.	.	+	+	+	.	.	.	.	.	.	.	.	3
*Ranunculus trilobus*	.	.	.	.	.	.	.	.	.	.	.	.	.	.	.	.	.	.	.	1	.	.	.	.	.	.	.	+	+	.	.	3
*Chamaemelum fuscatum*	.	.	.	.	.	.	.	.	.	.	.	.	.	.	.	.	.	+	.	+	.	+	.	.	.	.	.	.	.	.	.	3
*Juncus bufonius*	.	.	.	.	.	.	.	+	.	.	.	.	.	+	.	.	.	.	.	.	.	.	.	.	.	.	.	.	.	.	.	2
**Other species**																																
*Polypogon monspeliensis*	+	+	+	.	.	+	1	+	+	.	.	.	.	+	+	1	+	+	.	+	.	+	+	1	1	1	+	1	+	+	+	23
*Atriplex patula*	.	.	.	.	.	.	.	.	.	.	.	.	.	.	.	1	1	1	+	+	1	+	1	+	+	+	.	.	.	1	2	13
*Ecballium elaterium*	.	.	.	.	.	+	+	.	.	1	1	1	1	.	.	.	.	+	+	+	.	.	.	.	.	.	+	+	1	.	+	13
*Symphyotrichum squamatum*	.	.	.	.	.	.	.	.	.	.	.	.	.	.	.	.	+	+	+	+	+	+	+	.	.	.	.	+	+	+	+	11
*Suaeda spicata*	+	.	+	.	+	+	+	+	.	+	.	+	+	+	.	.	.	.	.	.	.	.	.	.	.	.	.	.	.	.	.	10
*Spergularia marina*	+	.	.	.	.	+	1	+	1	.	.	.	.	.	.	.	.	.	.	.	.	.	.	.	.	.	.	.	.	.	.	5
*Polypogon maritimus*	.	.	.	.	.	.	.	.	.	.	.	.	.	.	.	.	.	1	+	+	+	1	.	.	.	.	.	.	.	.	.	5
*Kikxia spuria* subsp. *integrifolia*	.	.	.	.	.	.	.	.	.	.	.	.	.	.	.	.	.	.	.	.	.	.	.	.	.	.	+	+	.	1	1	4
*Heliotropium europaeum*	.	.	.	.	.	.	.	.	.	.	.	.	.	.	.	.	.	+	+	+	+	.	.	.	.	.	.	.	.	.	.	4
*Juncus subulatus*	.	.	.	.	.	.	.	.	.	.	.	.	.	.	.	.	.	+	+	+	.	+	.	.	.	.	.	.	.	.	.	4
*Anagallis arvensis*	.	.	.	.	.	.	.	.	.	.	.	.	.	.	.	.	.	.	.	+	+	.	+	.	.	.	.	.	.	+	.	4
*Beta vulgaris maritima*	.	.	.	.	.	.	.	.	.	.	.	.	.	.	.	.	.	+	.	.	.	.	.	.	.	.	+	1	+	.	.	4
*Trigonella sicula*	.	.	.	.	.	.	.	.	.	.	.	.	.	.	.	.	.	.	.	+	+	+	.	.	.	.	.	.	.	.	.	3
*Geranium dissectum*	.	.	.	.	.	.	.	.	.	.	.	.	.	.	.	.	.	.	.	+	+	.	.	.	.	.	.	.	.	.	.	2

**Localities and dates of relevés.** Rel. 1–9: Piana del Signore (Gela)—8 September 1973; rel. 10–14: Piana del Signore (Gela)—20 August 1978; rel. 15: Piana del Signore (Gela)—1 August 2003; rel. 16–17: Piana del Signore (Gela), 19 August 2003; rel. 18–21: Piana del Signore (Gela)—12 June 2005; rel. 22: Piana del Signore (Gela)—19 June 2005; rel. 23—Piana del Signore (Gela)—6 August 2005; rel. 24–26: Contrada Margi (Gela)—1 August 2005; rel. 27–29: Macchitella (Gela)—20 August 2005; rel. 30–31: Contrada Bucrazzi (Gela)—10 September 2005.

**Table A32:** *Ranunculo trilobi-Lythretum tribracteati* Brullo & Sciandrello ass. nov.

Relevé number	1	2	3	4	5	6	Presences
Altitude (m)	9	9	9	9	9	9	
Surface (m^2^)	3	5	5	5	10	20	
Coverage (%)	60	80	90	90	100	100	
**Char. Association**							
*Ranunculus trilobus*	+	2	1	+	1	1	6
**Char. All. (LYTHRION TRIBRACTEATI)**							
*Lythrum tribracteatum*	2	2	3	4	5	5	6
**Char. Ord. (NANOCYPERETALIA)**							
*Pulicaria sicula*	1	+	1	2	3	1	6
*Hordeum marinum*	.	+	+	1	1	+	5
*Schenkia spicata*	.	1	1	2	+	+	5
*Coronopus squamatus*	.	1	1	.	.	.	2
**Char. Class (ISOËTO-NANOJUNCETEA)**							
*Mentha pulegium*	2	4	5	1	1	2	6
*Polypogon subspathaceus*	3	+	1	+	1	+	6
*Lythrum hyssopifolia*	3	3	2				3
*Juncus hybridus*	3	+	1	.	.	.	3
*Damosonium bourgaei*	+	.	.	+	+	.	3
*Gaudinia fragilis*	.	1	+	.	.	.	2
*Juncus bufonius*	1	.	.	.	.	.	1
**Other species**							
*Trifolium resupitanum*	2	1	1	.	.	.	3
*Centaurium tenuiflorum*	+	+	+	.	.	.	3
*Anagallis arvensis*	+	1	1	.	.	.	3
*Trigonella sicula*	.	+	.	+	.	+	3
*Chamaemelum fuscatum*	2	+	+	.	.	.	3
*Bromus hordeaceus*	1	+	+	.	.	.	3
*Atriplex patula*	.	.	1	+	+	.	3
*Symphyotrichum squamatum*	.	.	+	+	.	+	3
*Anagallis arvensis*	.	1	1	.	.	.	2
*Polygonum aviculare*	.	+	+	.	.	.	2
*Plantago coronopus*	.	+	1	.	.	.	2
*Podospermum canum*	.	+	+	.	.	.	2
*Cressa cretica*	.	+	1	.	.	.	2
*Medicago ciliaris*	1	+	.	.	.	.	2
*Visnaga daucoides*	.	.	+				1
*Phalaris minor*	.	.	+	.	.	.	1
*Carduus argyroa*	.	.	+	.	.	.	1
*Scorzoneroides muelleri*	1	.	.	.	.	.	1

**Localities and dates of relevés.** Rel. 1: Piana del Signore (Gela)—15 May 2003; rel. 2: Piana del Signore (Gela)—30 May 2004; rel. 3: Piana del Signore (Gela)—20 June 2004; rel. 4–6: Contrada S. Oliva (Gela)—3 September 2005.

**Table A33:** *Pulicario graecae-Damasonietum bourgaei* Minissale, Santo & Sciandrello 2011.

Relevé number	1	2	3	4	5	6	Presences
Altitude (m)	18	18	18	18	18	18	
Surface (m^2^)	5	5	10	1	20	30	
Coverage (%)	70	70	70	50	70	60	
**Char. Association**							
*Pulicaria vulgaris* var. *graeca*	3	3	1	2	3	+	6
**Char. All. (LYTHRION TRIBRACTEATI ) and Ord. (NANOCYPERETALIA)**							
*Heliotropium supinum*	+	+	+	.	.	+	4
*Lythrum tribracteatum*	.	2	.	1	.	.	2
*Pulicaria sicula*	.	.	+	.	+	.	2
*Coronopus squamatus*	.	.	.	+	+	.	2
*Hordeum marinum*	.	.	+	.	.	.	1
*Schenkia spicata*	.	.	.	.	1	.	1
**Char. Class (ISOËTO-NANOJUNCETEA)**							
*Damasonium bourgaei*	4	3	4	3	1	3	6
*Polypogon subspathaceus*	+	+	+	.	1	.	4
**Other species**							
*Bolboschoenus maritimus*	2	1	2	+	2	3	6
*Limonium narbonense*	1	+	+	+	2	.	5
*Polypogon maritimus*	1	1	+	.	+	.	4
*Trifolium resupinatum*	.	.	+	+	.	.	2
*Juncus subulatus*	+	.	.	.	.	.	1
*Dittrichia viscosa*	.	.	.	.	+	.	1

**Localities and dates of relevés.** Rel. 1–6: Capo Murro di Porco, Maddalena Peninsula (Syracuse), Minissale et al. [92]—Table 3.
